# Bioactive Polymeric Scaffolds: Multivalent Functionalization by Thermal Azide–Alkyne Cycloaddition with Alkynyl Dicarbamates

**DOI:** 10.1021/acs.biomac.5c00038

**Published:** 2025-03-26

**Authors:** Maun H. Tawara, Juan Correa, Emma Leire, Bruno Delgado Gonzalez, Samuel Parcero-Bouzas, Flonja Liko, Eduardo Fernandez-Megia

**Affiliations:** Centro Singular de Investigación en Química Biolóxica e Materiais Moleculares (CiQUS), Departamento de Química Orgánica, 16780 Universidade de Santiago de Compostela, Jenaro de la Fuente s/n, 15782 Santiago de Compostela, Spain

## Abstract

Multivalency enables interactions with higher affinities and specificities than monovalent interactions. The strategy exploited by nature to modulate biorecognition has inspired the design of multivalent conjugates with therapeutic properties. However, chemical functionalization often requires coupling agents, additives, or metal catalysts that complicate isolation and purification. Herein, azide–alkyne cycloaddition (AAC) with alkynyl dicarbamates (Alk-R) is presented as a flexible, reliable, atom-economical, and user-friendly strategy for the multivalent functionalization of polymeric scaffolds. Alk-R functionalized with biologically relevant ligands have been prepared and used for the multivalent AAC functionalization of azide-bearing dendrimers and block copolymers. The resulting polymers with double multivalency reveal a platform for the development of bioinspired functional systems with promising applications in drug delivery: block copolymer micelles and multifunctional nanocarriers with synergistically integrated probes-ligands-drugs. The extension of this strategy to other ligands and scaffolds is expected to open up a wide range of therapeutic and diagnostic opportunities.

## Graphical abstract

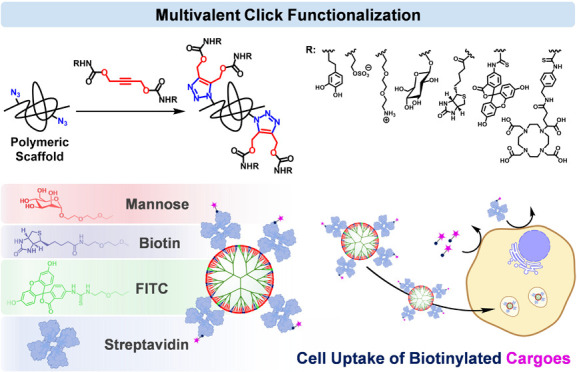

## Introduction

Multivalency plays a central role in nature.^1^ The multivalent presentation of ligands and receptors on biological surfaces enables interactions with higher affinities and specificities than monovalent interactions. This strategy exploited by nature to modulate biorecognition has inspired the design of multivalent synthetic conjugates with therapeutic properties such as vaccines, immunomodulators, cell signaling effectors, and polymers for drug and gene delivery applications.^2,3^

Myriad biological processes are regulated by multivalent interactions between glycans and carbohydrate-binding proteins (lectins), including pathogen infection, self-recognition, and the immune response.^4–6^ The efficiency of the synthesis of multivalent glycoconjugates has made it possible to create synthetic equivalents that resemble the diversity and complexity found in nature. These systems have applications in tissue engineering, vaccines, sensing, therapeutic and diagnostic tools,^7–9^ and drug delivery to cells that overexpress lectin receptors.^10^ Similarly, the multivalent nature of cationic polymers makes them ideal biomaterial platforms for gene therapy due to their ability to condense and deliver nucleic acid payloads to target cells and tissues.^11–13^ Multivalency is also being considered as a strategy to improve binding and detection sensitivity in biosensors.^14–16^ In another example, multivalent protein constructs have been designed to increase the efficiency of protein-drug conjugates^17^ and small affinity proteins.^18^ For instance, several reports have described the enhanced binding affinity of multimeric antibody mimics targeting the trimeric spike protein of SARS-CoV-2.^19^

Unfortunately, chemical functionalizations involved in the synthesis of multivalent systems typically require coupling agents, additives, or metal catalysts that complicate the isolation and purification of the final conjugates.^20^ In addition, these processes rarely increase multivalency.^21^ Therefore, innovations in multivalent functionalization are still awaited, with the ultimate goal of providing more user-friendly and greener strategies to nonspecialists.

Among the synthetic multivalent nanoplatforms, dendrimers stand out for their unprecedented ability to tune size and multivalency.^22^ Dendrimers are composed of repetitive layers of branching units prepared in a controlled iterative fashion through generations (G) with discrete properties. Their quantized nature allows a degree of control over properties/applications coined as the “dendritic effect.”^23^ We have recently described an accelerated synthesis of dendrimers^24^ and PEG-dendritic block copolymers^25^ [PEG is poly­(ethylene glycol)] based on the Huisgen thermal azide–alkyne cycloaddition (AAC)^26^ with acetylenedicarboxylates, a click reaction that proceeds with complete atom economy. The strategy is amenable to high chemical diversity by tuning the alkyne substituents. In this way, the newly formed triazole branches not only double the multivalency of the system but also emerge as a key structural element for adjusting dendritic properties.

Here, we describe our efforts to adapt this technology to the multivalent functionalization of polymeric scaffolds. To ensure hydrolytic stability and broad functional group compatibility, we have focused on internal alkynes with a but-2-yne-1,4-diyl dicarbamate structure (Alk-R, Figure 1). Alk-R functionalized with alcohols, cationic and anionic groups of interest for drug and gene delivery applications, biologically relevant ligands, fluorescent probes, and metal-chelating agents for imaging and radiotherapy have easily been prepared in two steps and their utility for multivalent AAC functionalization assessed with azide-bearing dendrimers (several G), linear-dendritic, and linear-linear block copolymers. This has resulted in a collection of functionalized dendritic and dendronized polymers, some of which have been evaluated in the development of several drug delivery applications.

**Figure 1:**
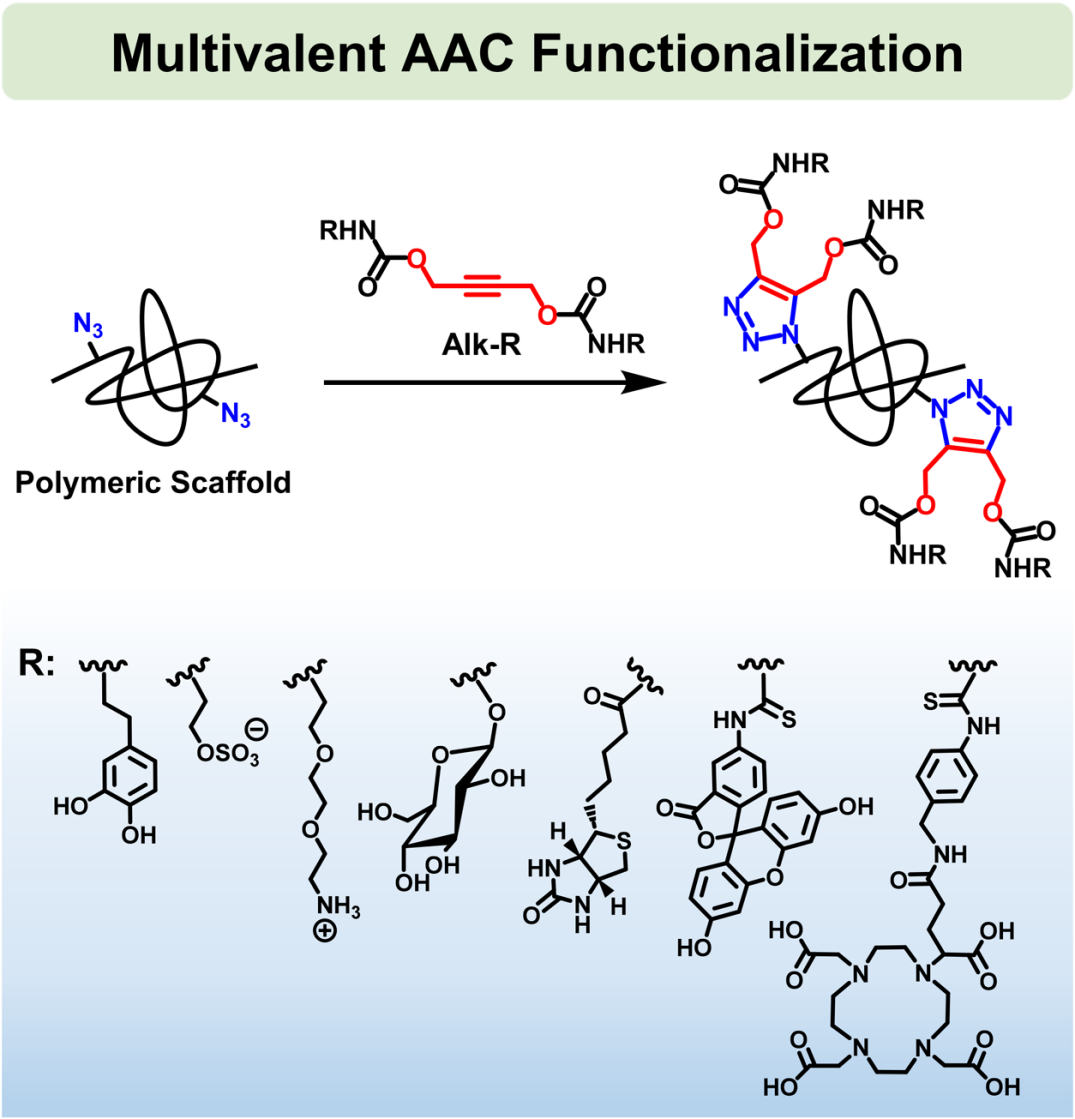
Multivalent AAC functionalization of polymeric scaffolds.

## Materials and Methods

### Materials

p-NCS-Bz-DOTA-GA (2,2′,2″-(10-(1-carboxy-4-((4-isothiocyanatobenzyl)­amino)-4-oxobutyl)-1,4,7,10-tetraazacyclododecane-1,4,7-triyl)­triacetic acid) was purchased from CheMatech. Sulfo-Cyanine5-PEG_3_-biotin (Bio-Cy5) was purchased from Lumiprobe. All other chemicals were purchased from Merck or Acros and used without further purification. All solvents were of HPLC grade, purchased from Scharlab, Merck, or Fisher Scientific and used without further purification. *t*-BuOH was of reagent grade and was used without further purification. DMSO, Et_3_N, and pyridine were dried under 4 Å molecular sieves. MeOH was distilled from CaH_2_. CH_3_CN, DMF, THF, and CH_2_Cl_2_ were dried using an SPS800 solvent purification system from MBRAUN. H_2_O of Milli-Q grade was obtained using a Millipore water purification system. PEG–PGA-N_3_ (PEG_5k_, DP_PGA_ 23 by ^1^H NMR),^27^ PEG-[G2]-N_3_ (PEG_5k_ by MALDI-TOF),^28^ azido functionalized GATG-dendrimers (3­[G1]-N_3_, 2­[G2]-N_3_, 3­[G3]-N_3_, and 3­[G4]-N_3_),^29^ 2-[2-(2-azidoethoxy)­ethoxy]­ethanol,^30^ 2-[2-(2-aminoethoxy)­ethoxy]-ethanol,^31^ *tert*-butyl (2-(2-(2-aminoethoxy)­ethoxy)­ethyl)­carbamate,^32^ and 2,3,4,6-tetra-*O*-acetyl-α-d-mannopyranosyl trichloroacetimidate and 2,3,4,6-tetra-*O*-acetyl-β-d-glucopyranosyl trichloroacetimidate^33,34^ were prepared following known procedures.

### Column Chromatography

Automated column chromatography was performed on an MPLC Teledyne ISCO CombiFlash RF 200 psi. Samples were adsorbed onto silica gel (40–63 μm from VWR Chemicals) or neutral alumina (70–230 mesh from Merck) and loaded into solid cartridges. RediSep RF columns refilled with silica (40–63 μm, VWR Chemicals) or neutral alumina (70–230 mesh, Merck) were used.

### Ultrafiltration

Purifications by ultrafiltration were performed on Millipore Amicon stirred cells with Amicon YM1, YM3, or YM5 regenerated cellulose membranes under 5 psi of N_2_ pressure.

### NMR Spectroscopy

NMR spectra were recorded on Varian Mercury 300 MHz, Bruker DRX 500 MHz, and Bruker NEO 750 MHz spectrometers. Chemical shifts were reported in ppm relative to the residual solvent peak (7.26 ppm for CDCl_3_, 3.31 ppm for CD_3_OD, 4.79 ppm for D_2_O, 2.5 ppm for DMSO*-d*_6_, and 1.94 ppm for CD_3_CN). ^1^H-diffusion filter experiments were done using a Stimulated-Echo-LED pulse sequence with bipolar PFG gradients, relaxation delay (d1) was set to 1.5 s, and diffusion delay (Δ) was set to 50 or 100 ms. MestReNova 14.2 software (Mestrelab Research) was used for spectral processing.

### Infrared Spectroscopy

FT-IR spectra were recorded on a Bruker IFS-66v or a PerkinElmer Spectrum Two spectrophotometer using KBr pellets or neat samples (CsI window).

### Mass Spectrometry

MALDI analysis was performed in a 4800 MALDI-TOF/TOF analyzer (Applied Biosystems, Foster City, CA). Spectra were acquired in linear mode (20 kV source) with a Nd:YAG (355 nm) laser and averaging 1000 laser shots. The mass of functionalized dendrimers was determined by reference to a peptide Standard I (Bruker-Daltonics), composed of insulin (*m*/*z* 5734.51), ubiquitin I (*m*/*z* 8565.76), cytochrome C (*m*/*z* 12360.97), myoglobin (*m*/*z* 16952.30), cytochrome C (*m/2z* 6180.99), and myoglobin (*m*/2*z* 8476.65). The dried-droplet method was used to deposit 1 μL of a dendrimer and matrix mixture onto a 384 Opti-TOF MALDI plate (Applied Biosystems, Foster City, CA). Lyophilized 3­[G2]–OH was dissolved in H_2_O-MeOH 1:1 (5 mg/mL). Then, 1 μL of this solution was mixed with 30 μL of a 2-(4-hydroxyphenylazo)­benzoic acid (HABA, ref 54793, Merck) solution (0.05 M in dioxane) and analyzed by MALDI-TOF. Lyophilized 2­[G3]–OH was dissolved in H_2_O-MeOH 1:1 (5 mg/mL). Then, 1 μL of this solution was mixed with 20 μL of a sinapic acid (ref 85429, Merck) solution (10 mg/mL in MeOH) and analyzed by MALDI-TOF.

Electrospray ionization (ESI) flow injection analysis (FIA) time-of-flight (TOF) mass spectrometry was carried out on a Tandem HPLC instrument (Agilent 1100)-MS (Bruker Microtof). The ESI source was equipped with a gas flow countercurrent of 1 μL/min under a temperature control.

### Ultraviolet–Visible (UV–Vis) Spectroscopy

UV–vis measurements were recorded on a Jasco V-630 spectrophotometer.

### Elemental Analysis

Samples were analyzed on a Thermo Finnigan Flash 1112 elemental analyzer.

### Gel Permeation Chromatography (GPC)

GPC experiments were performed on an Agilent 1100 series separation module using a polyanionic and neutral column type composed of a PSS SDV precolumn (5 μm, 8 mm × 50 mm) and a Suprema Lux 100 Å connected to an Agilent 1100 series UV detector (254 nm). A 10 mM PB pH 7.4, 150 mmol of LiCl solution was used as eluent at 1 mL/min. Samples at 1 mg/mL were filtered through 0.45 μm nylon filters before injection.

### Dynamic Light Scattering (DLS)

DLS measurements were carried out on a Malvern Nano ZS (Malvern Instruments, U.K.) operating at 633 nm with a 173° scattering angle at 25 °C. Hydrodynamic diameters were measured in 10 mM PB pH 7.4, 150 mM LiCl at a concentration of 1.0 mg/mL and 25 °C. Mean diameters were obtained from the volume particle size distribution provided by the Malvern Zetasizer Software.

### Transmission Electron Microscopy (TEM)

TEM measurements were performed on a JEOL JEM-1011 operated at 100 kV electron microscope equipped with a camera S5MegaView G2. A drop of a solution of PEG-[G3]-Cat micelles (1 mg/mL in H_2_O) was settled on a TEM carbon type-B film copper grid (Ted Pella, Inc.) and allowed to dry at room temperature for 5 min. The size of micelles was determined with ImageJ software (version 1.51j8), measuring the line intensity profile across the assemble. An average diameter of 21 ± 2 nm was obtained by measuring the size of 40 micelles.

## Results and Discussion

### Synthesis of Functionalized Internal Alkynes (Alk-R)

The preparation of Alk-R was easily accomplished by the reaction of commercially available 2-butyne-1,4-diol with *N*,*N*′-disuccinimidyl carbonate (DSC, py, MeCN, 92%), followed by treatment of Alk-NHS with a variety of amino-functionalized linkers, incorporating terminal alcohols or a Boc-protected amine. Alk-R shown in Figure 2A were obtained in excellent yields (≥90%). While Alk-OH and Alk-TEG-OH display typical solubilizing groups aimed at improving the biocompatibility of polymeric scaffolds, Alk-PhOH and Alk-Cat are amphiphiles envisaged for self-assembly processes. Moreover, the catechol groups of Alk-Cat can serve as chemical handles for dynamic covalent chemistry endeavors with boronic acids.^13^ As will be shown below, the deprotection of Alk-TEG-NHBoc releases a primary amine available for further functionalizations.

**Figure 2:**
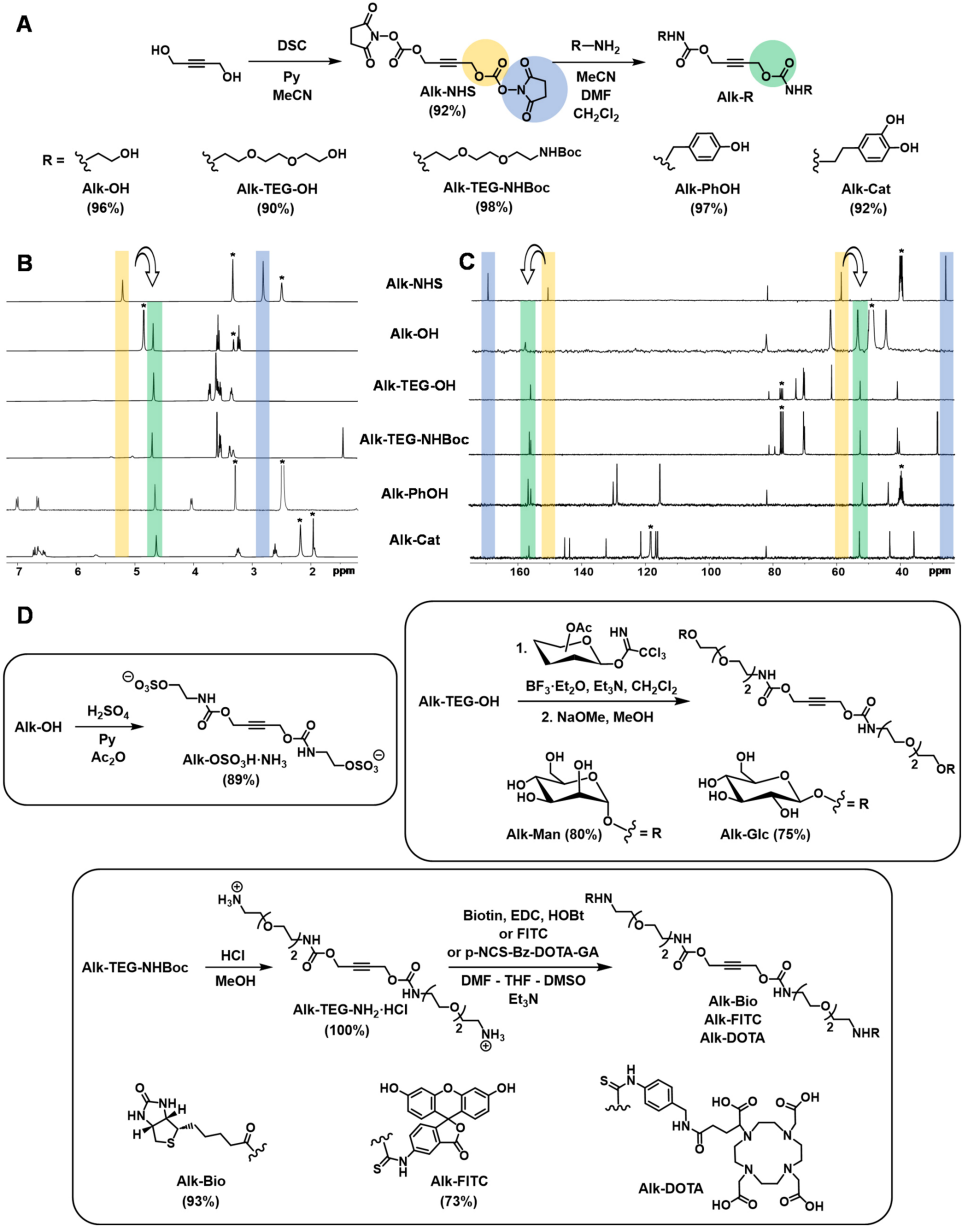
Synthesis of functionalized internal alkynes Alk-R (A) and monitoring of their preparation by ^1^H (300 MHz, B) and ^13^C NMR (75 MHz, C); * indicates the residual solvent signal. Functionalization of Alk-R with ligands of biomedical interest (D).

Completion of the synthesis of Alk-R was easily monitored by ^1^H NMR by the disappearance of a proton signal of NHS at around 2.80 ppm (Figure 2B). In addition, the propargyl protons of Alk-NHS at around 5.10 ppm are shifted to 4.65–4.75 ppm in the functionalized alkynes. ^13^C NMR was also useful to confirm the completion of the reactions and the purity of Alk-R, thanks to the disappearance of two NHS signals at about 170.0 ppm (carbonyl) and 25.5 ppm (methylene), as well as the shift of the carbonate signal from ca. 151.0 ppm to 155.0–157.0 ppm (carbamate) and the propargyl signal from ca. 58.5 to 52.0–53.0 ppm (Figure 2C).

The incorporation of structurally complex ligands of biomedical interest into Alk-R is shown in Figure 2D. Anionic and cationic groups, sugars, biotin, a fluorescent probe (fluorescein), and the metal-chelating agent 1,4,7,10-tetraazacyclododecane-1,4,7,10-tetraacetic acid (DOTA) have been successfully incorporated into Alk-R. For example, sulfation of Alk–OH (H_2_SO_4_, Ac_2_O, py)^35^ afforded anionic Alk-OSO_3_H·NH_3_ in 89% yield, while deprotection of Alk-TEG-NHBoc (HCl, MeOH) yielded cationic Alk-TEG-NH_2_·HCl quantitatively. Multivalent AAC functionalization of polymers with Alk-OSO_3_H·NH_3_ and Alk-TEG-NH_2_·HCl is envisaged to provide polyelectrolytes of interest for drug and gene delivery applications.^11,36^

Carbohydrate–lectin interaction is by far the most studied multivalent interaction because of its relevance in cell–cell recognition, fertilization, pathogen invasion, and toxin and hormone mediation.^4,6,14^ With the aim of preparing multivalent synthetic glycoconjugates with the ability to promote or inhibit multivalent carbohydrate–lectin interactions,^7,9^ d-mannose- and d-glucose-functionalized alkynes were synthesized from Alk-TEG-OH using acetylated mannosyl and glucosyl trichloroacetimidates (BF_3_·Et_2_O, Et_3_N, CH_2_Cl_2_). After complete deacetylation under Zemplén conditions (cat NaOMe, MeOH),^37^ Alk-Man and Alk-Glc were obtained in 80 and 75% overall yields (Figure 2D).

Alk-TEG-NH_2_·HCl was used for the incorporation of biotin, fluorescein, and DOTA into Alk-R (Figure 2D). Biotin is a ligand of great interest because of its highly specific noncovalent interaction with the tetrameric proteins avidin and streptavidin, resulting in powerful assay, detection, and targeting systems.^38^ Fluorescein was selected as a typical fluorescent probe for cell trafficking studies, while DOTA is a classical metal-chelating agent for in vivo imaging^39^ and radiotherapy.^40^ Treatment of Alk-TEG-NH_2_·HCl with biotin (EDC, HOBt), fluorescein isothiocyanate (FITC), and p-NCS-Bz-DOTA-GA afforded the desired functionalized alkynes in very good to excellent yields.

### Multivalent AAC Functionalization of Polymeric Scaffolds

With an extensive Alk-R library available, the efficacy of AAC for multivalent functionalization was assessed with Alk–OH and 3­[G1]-N_3_, a small dendrimer of the gallic acid-triethylene glycol (GATG)^28–30^ family, bearing 9 terminal azides. Under optimized reaction conditions (1 M of azide, 2 equiv of Alk–OH per azide, *t*-BuOH/H_2_O 5:1, 8 h, 120 °C), 3­[G2]–OH with 18 peripheral alcohols was obtained in 93% yield (Figure 3 and Table 1). When identical reaction conditions were applied to more structurally complex alkynes, including Alk-TEG-OH, Alk-Man, and Alk-Glc, the corresponding functionalized dendrimers 3­[G2]-TEG–OH, 3­[G2]-Man, and 3­[G2]-Glc were also obtained in excellent yields (90–92%, Figure 3 and Table 1). Notably, simple ultrafiltration enabled the purification of these dendrimers and the recovery of the excess alkynes.

**Figure 3:**
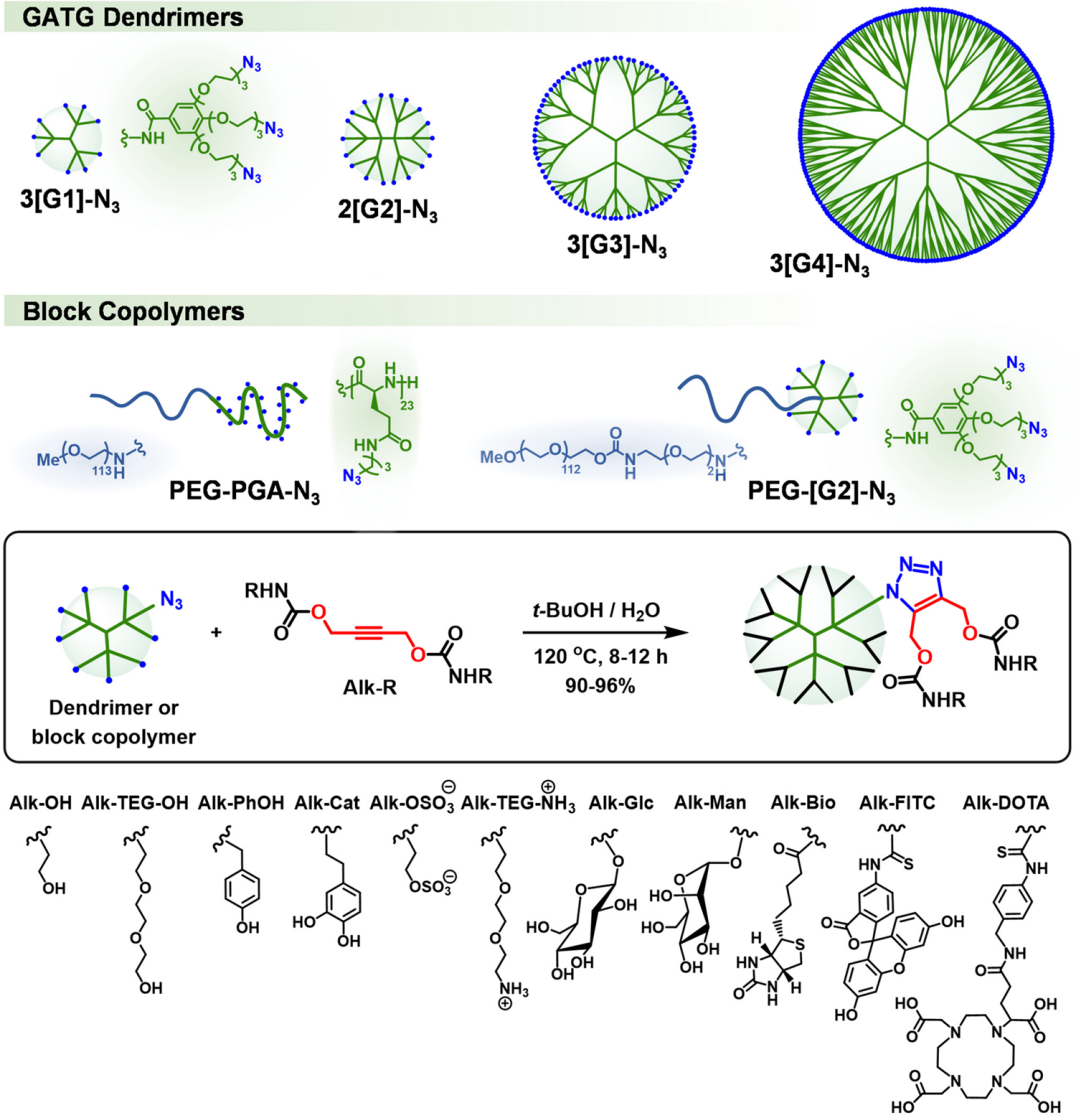
Multivalent AAC functionalization of polymeric scaffolds with biologically relevant probes and ligands.

**Table 1:** Multivalent AAC Functionalization of Polymeric Scaffolds with Alk-R

polymeric scaffold	Alk-R	functionalized scaffold	yield (%)	*D*_h_ (nm)^a^	multivalency
3[G1]-N_3_	Alk–OH	3[G2]–OH	93	2.6	18
	Alk-TEG–OH	3[G2]-TEG–OH	90	3.1	
	Alk-Man	3[G2]-Man	92	3.6	
	Alk-Glc	3[G2]-Glc	91	3.7	
2[G2]-N_3_	Alk–OH	2[G3]–OH	94	3.4	36
	Alk-TEG-NH_2_·HCl	2[G3]-TEG-NH_2_·HCl	93	4.3	
3[G3]-N_3_	Alk–OH	3[G4]–OH	91	6.1	162
	Alk-TEG–OH	3[G4]-TEG–OH	92	8.2	
	Alk-PhOH	3[G4]-PhOH	91	9.1^b^	
	Alk-OSO_3_H·NH_3_	3[G4]-OSO_3_Na	91	7.0 (7.1)^c^	
	Alk-Man	3[G4]-Man	93	8.7	
	Alk-Glc	3[G4]-Glc	92	8.6	
	Alk-TEG-NH_2_·HCl	3[G4]-TEG-NH_2_·HCl	92	8.2	
	Alk-DOTA	3[G4]-DOTA	90^d^	12.7	
	Alk-Man Alk-Bio Alk-FITC	3[G4]-Man_120_/Bio_32_/FITC_10_	96	9.4	
3[G4]-N_3_	Alk-Man	3[G5]-Man	94	12.0	486
PEG-[G2]-N_3_	Alk-Cat	PEG-[G3]-Cat	92		18
PEG–PGA-N_3_	Alk–OH	PEG–PGA-[G1]–OH	92	5.7	46

a Hydrodynamic diameter determined by DLS (1 mg/mL in 10 mM PB pH 7.4, 150 mM LiCl, 25 °C).

b Determined by DLS (1 mg/mL in THF, 25 °C).

c Determined by DOSY (0.5 mg/mL in D_2_O, 750 MHz).

d Prepared following a one-pot procedure (synthesis of Alk-R and multivalent AAC functionalization).

Next, the robustness and fidelity of the multivalent AAC functionalization were challenged with larger dendrimers, such as 2­[G2]-N_3_ and 3­[G3]-N_3_ (18 and 81 terminal azides), and Alk-R carrying biologically relevant probes and ligands (Figure 3). As shown in Table 1, all AAC provided the expected dendrimers functionalized in yields higher than 90%, regardless of the dendrimer and Alk-R. Even a giant dendrimer, such as 3­[G4]-N_3_ with 243 terminal azides, was efficiently decorated with Alk-Man to give 3­[G5]-Man in 94% yield; a glycodendrimer with a theoretical functionalization of 486 mannoses and a molecular weight greater than 257 kDa, exceeding that of most glycoproteins in nature. Interestingly, a one-pot procedure was implemented for the synthesis of Alk-R and in situ AAC scaffold functionalization, which is especially useful for Alk-R derivatives of difficult isolation/purification. Application of this protocol to Alk-DOTA and 3­[G3]-N_3_ produced 3­[G4]-DOTA in a 90% yield (Table 1). The scope of the multivalent AAC functionalization was also investigated with linear polymers (Figure 3). For example, a block copolymer composed of PEG and poly-l-glutamic acid with pendant azides (PEG–PGA-N_3_; PEG_5k_, DP_PGA_ 23)^27^ was efficiently functionalized with Alk–OH to give dendronized PEG–PGA-[G1]–OH in excellent yield (92%, Table 1).

The progress of the multivalent AAC functionalization was readily monitored by ^1^H and ^13^C NMR and IR as shown in Figure 4 for the mannosylated dendrimers as a representative example. IR was extremely useful in this respect by following the disappearance of the characteristic intense azide band at ca. 2100 cm^–1^ (Figure 4C). The completion of AAC was also demonstrated by ^1^H NMR (Figure 4A) by the disappearance of a signal at ca. 3.40 ppm due to the methylene protons adjacent to the azide group being shifted to 4.40–4.80 ppm in the conjugates. The appearance of a new set of two multiplets between 5.00 and 5.50 ppm, corresponding to the methylene protons in α to the triazole (original propargyl protons of Alk-R at ca. 4.65–4.75 ppm), also confirmed the structural integrity of the functionalized dendrimers. ^13^C NMR also helped in monitoring the completion of the AAC coupling (Figure 4B) due to the disappearance of the methylene carbon adjacent to the azide group at ca. 51.0 ppm (shifted to 48.0–49.0 ppm after functionalization) and the appearance of two new signals at ca. 132 and 142 ppm corresponding to the triazole carbons (original alkyne carbons of Alk-R at ca. 81–84 ppm). In addition, MALDI-TOF MS showed molecular weights in very good agreement with the calculated values. No additional peaks associated with incomplete AAC functionalizations were seen in the spectra, in agreement with the NMR and IR data. The purity and monodispersity of the functionalized scaffolds were further confirmed by dynamic light scattering (DLS; Figures 4D and S1 and Table 1) and gel permeation chromatography (GPC; Figures 4E and S2), which showed the expected increase in size after the AAC functionalization [e.g., hydrodynamic diameters of 3­[G2]-Man (3.6 nm), 3­[G4]-Man (8.7 nm), and 3­[G5]-Man (12.0 nm)].

**Figure 4:**
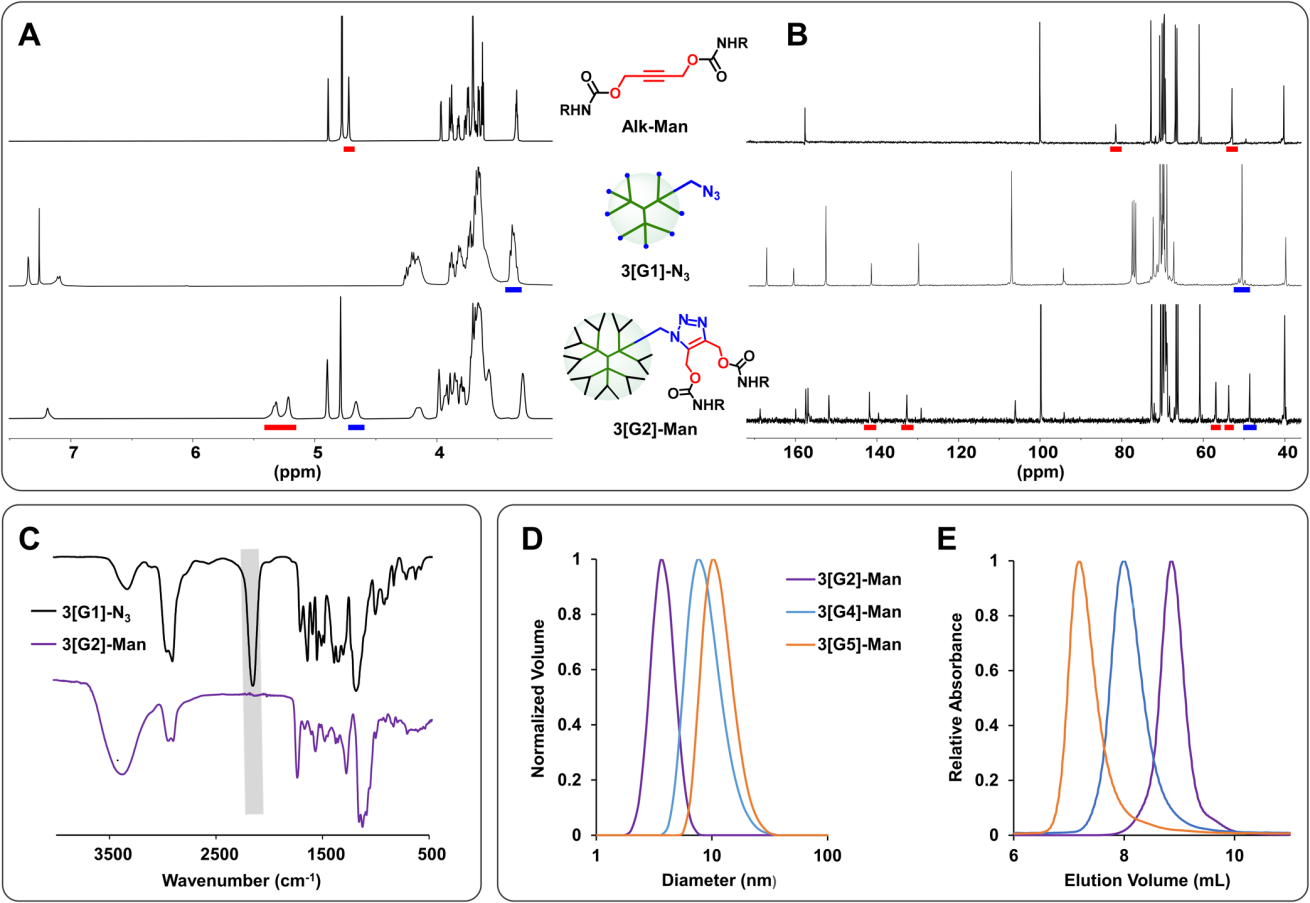
Characterization of functionalized scaffolds and monitoring of multivalent AAC functionalization: ^1^H (500 MHz), (A) and ^13^C NMR (125 MHz), (B) spectra of Alk-Man, 3­[G1]-N_3_, and 3­[G2]-Man. IR spectra of 3­[G1]-N_3_ and 3­[G2]-Man (C). DLS size distributions (D) and GPC elugrams (E) of 3­[G2]-Man, 3­[G4]-Man, and 3­[G5]-Man.

Having confirmed the efficiency of AAC for the multivalent functionalization of polymeric scaffolds, the following sections explore the application of this technology to the preparation of polymeric micelles and multifunctional nanocarriers for drug delivery applications.

### Amphiphilic Block Copolymer Micelles for Drug Delivery

Amphiphilic linear–dendritic block copolymers are interesting materials with the ability to self-assemble in solution due to differences in the solubility of the blocks.^41–43^ The resulting assemblies (micelles and vesicles) can encapsulate drugs, protect them from surrounding media after in vivo administration, and deliver them to specific cells and organs.^44,45^ With the aim of studying the potential of the multivalent AAC functionalization in the synthesis of amphiphilic block copolymers, PEG-[G2]-N_3_,^28^ a copolymer composed of a PEG_5k_ and a G2 dendron with 9 pendant azides (Figure 3) was reacted with Alk-Cat (120 °C, 12 h, *t*-BuOH/H_2_O 1:1, 92%), an amphiphilic alkyne designed to promote self-assembly (Figure 5A). In addition to the NMR signature mentioned above, PEG-[G3]-Cat was unambiguously characterized by the appearance in the ^1^H NMR spectrum of typical catechol signals between 6.31 and 6.66 ppm, integrating for 54 protons (18 catechol rings). Micelles from PEG-[G3]-Cat were prepared by simply dissolving the copolymer in 10 mM phosphate buffer (PB) pH 7.4, 150 mM NaCl (1 mg/mL). After 1 h at 25 °C, micelle formation was confirmed by DLS, which showed highly monodisperse assemblies with a mean diameter of 39 nm (Figure 5B). Analysis of these micelles by TEM revealed spherical particles with an average diameter of 21 nm (Figure 5B).

**Figure 5:**
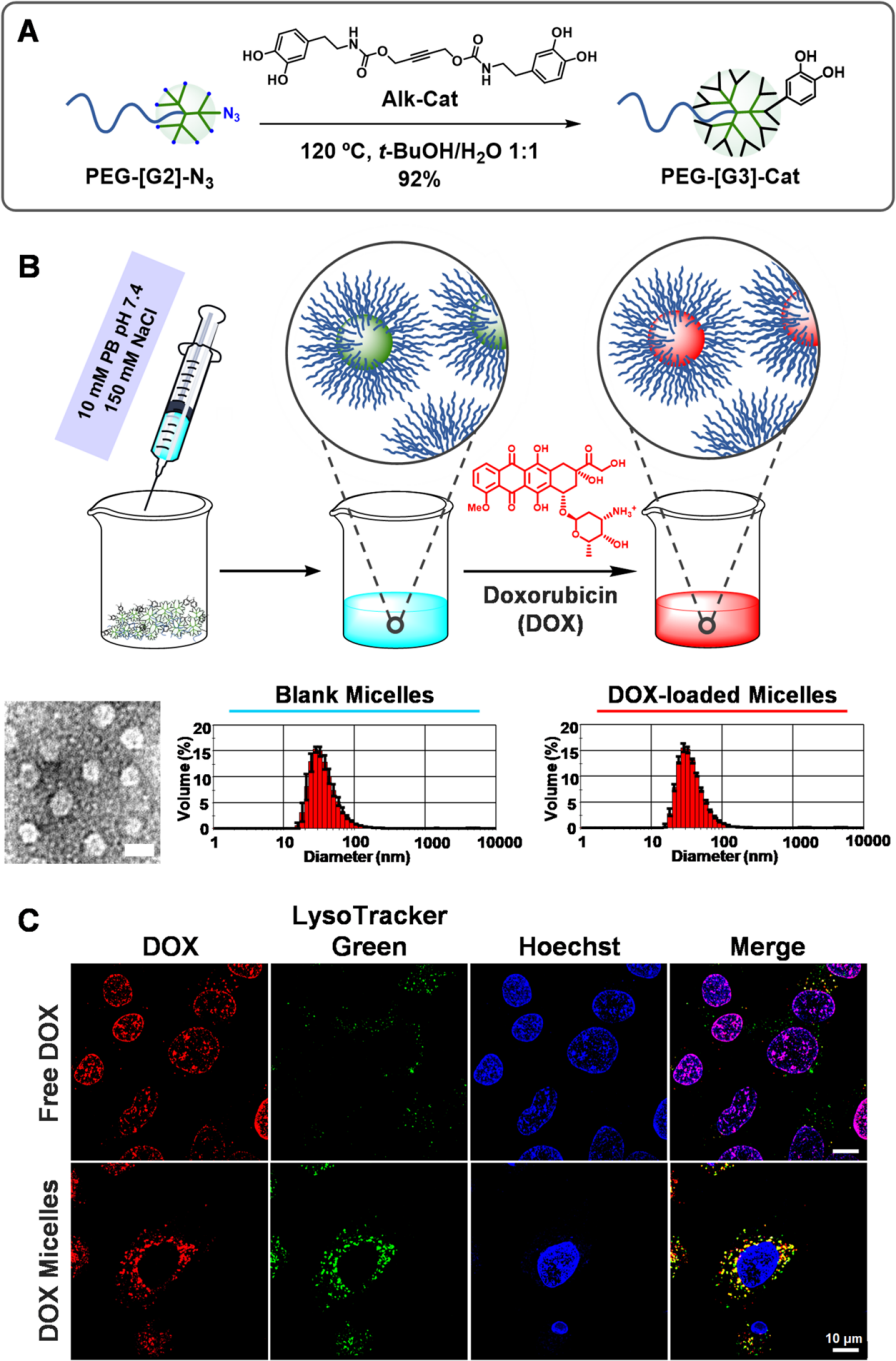
Synthesis of PEG-[G3]-Cat (A). Preparation of micelles from PEG-[G3]-Cat and encapsulation of doxorubicin (DOX). TEM image of blank micelles (scale bar: 25 nm). DLS histograms of blank and DOX-loaded micelles (10 mM PB pH 7.4, 150 mM NaCl) (B). Intracellular trafficking of free DOX and DOX-loaded micelles in A549 cells was assessed after 1.5 h of incubation by confocal microscopy (C).

The potential application of these micelles in drug delivery was assessed by analyzing their ability to encapsulate the anticancer drug doxorubicin (DOX) and promote its cell internalization. DOX is a frontline chemotherapy drug widely used to treat various cancers, lymphomas, and certain leukemias. Unfortunately, it has serious side effects, including life-threatening cardiotoxicity, which force a dose-limiting treatment and the search for efficient drug delivery systems.^46^ The encapsulation of DOX was achieved by adding an aqueous solution of the drug to freshly prepared PEG-[G3]-Cat micelles (50 mol % DOX relative to peripheral Cat groups), followed by dialysis against 10 mM PB pH 7.4, 150 mM NaCl to remove nonencapsulated DOX. As seen in Figure 5B, DOX-loaded micelles were indistinguishable from the blank micelles by DLS, providing an encapsulation efficiency (EE) of 87% and a drug loading (DL) of 29%. The ability of these micelles to tune the internalization and intracellular trafficking of DOX was studied in A549 cells by laser scanning confocal microscopy (LSCM, Figure 5C). After 1.5 h of incubation, free DOX (red) was selectively detected in the cell nuclei, as determined by its complete colocalization with Hoechst (blue). Because of its hydrophobic character, free DOX can cross cell membranes by diffusion and quickly migrate to the cell nuclei.^46,47^ In contrast, DOX in micelles colocalized exclusively with LysoTracker Green (green), a well-known endosome/lysosome marker. These findings confirm that PEG-[G3]-Cat can self-assemble into micelles that effectively encapsulate DOX and modify its cell internalization and trafficking pathway, highlighting the potential of this multivalent functionalization technology in the development of novel drug delivery systems.

### Multifunctional Dendritic Nanocarrier for Cell Internalization

After functionalizing various dendrimers (several G) and block copolymers by AAC with Alk-R of different nature, it became clear that neither the backbone architecture nor the ligand functionality significantly affects the kinetics of AAC. Encouraged by this reproducibility, the possibility of preparing multifunctional dendrimers by simultaneous AAC with different alkynes was investigated. Multifunctional nanocarriers with synergistically integrated probes, ligands, and drugs are gaining popularity because their cooperative properties can be exploited in therapy and diagnosis.^48,49^ Among these, the ability to precisely tune size and multivalency makes dendrimers particularly interesting.^50^

To this end, mannose and biotin were selected as recognition ligands of biomedical interest, and FITC was selected as a fluorescent probe. It was anticipated that the ratio between biotin, mannose, and FITC in a multifunctional dendrimer could easily be controlled by the feed ratio of alkynes in the reaction medium (Figure 6A). Thus, 3­[G3]-N_3_ (81 terminal azides) was reacted with Alk-Man, Alk-Bio, and Alk-FITC in a molar ratio of 120:32:10 (accounting for 2 equiv of alkyne per azide, 120 °C, *t*-BuOH/H_2_O 1:1). AAC was monitored by IR, which confirmed completion of the reaction after 14 h. Purification by ultrafiltration afforded 3­[G4]-Man_120_/Bio_32_/FITC_10_ in a 96% yield. As expected, an average incorporation of 10 FITC molecules per dendrimer was determined by absorbance at 494 nm, while degrees of substitution of 120 for mannose and 32 for biotin were determined by integration of characteristic signals in the ^1^H NMR spectrum (singlet at 4.90 ppm for mannose and multiplets at 2.64–2.93 and 2.12–2.30 ppm for biotin). The purity of the dendrimer conjugate was confirmed by ^1^H and ^13^C NMR and GPC. A hydrodynamic diameter of 9.4 nm was determined by DLS.

**Figure 6:**
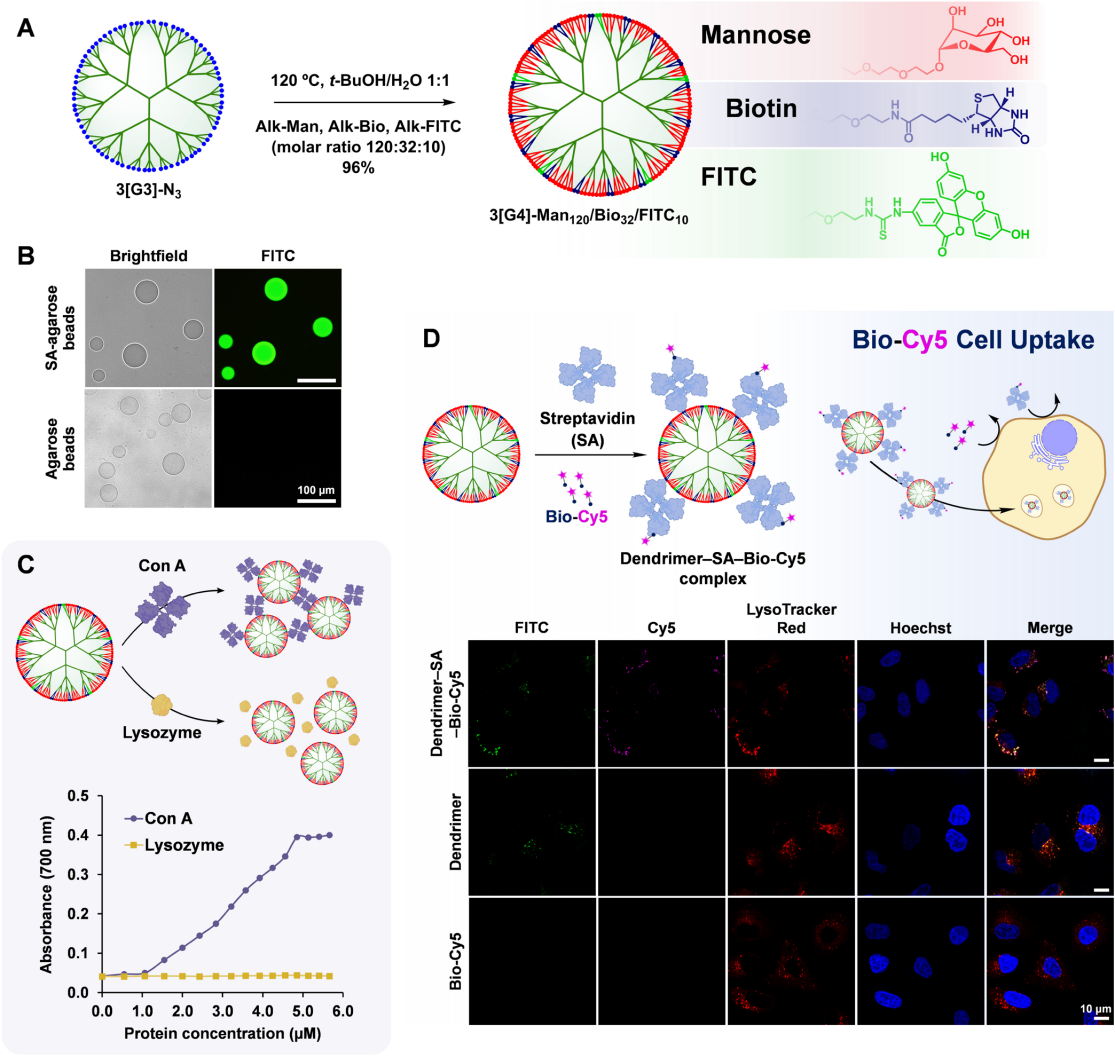
Synthesis of multifunctional dendrimer 3­[G4]-Man_120_/Bio_32_/FITC_10_ (A). Bioactivity of biotin: fluorescence microscopy image of SA–agarose beads selectively stained by the dendrimer (B). Bioactivity of mannose: aggregation of the dendrimer (300 μL, 1.67 mM mannose) with Con A (10 μL portions, 17 μM) in 20 mM Tris-HCl, 250 mM NaCl, 1 mM CaCl_2_, 1 mM MnCl_2_, pH 6.2 as determined by absorbance at 700 nm (C). Assessment of the dendrimer as a nanocarrier for cell internalization using Bio-Cy5 as a model cargo: preparation of the dendrimer–SA–Bio-Cy5 complex. LSCM images of A549 cells incubated with the dendrimer–SA–Bio-Cy5 complex for 2 h show colocalization of the dendrimer (FITC, green) and cargo (Cy5, magenta) with LysoTracker Red (red), confirming their successful combined internalization by endocytosis. Nuclei were stained in blue (Hoechst) (D).

The bioactivity of biotin on the surface of 3­[G4]-Man_120_/Bio_32_/FITC_10_ was verified by staining of agarose beads functionalized with streptavidin (SA–agarose). This recognition assay also exploits the fluorescent signal of FITC on the dendrimer. To this end, SA–agarose beads and blank beads taken as control (no SA) were separately incubated with 3­[G4]-Man_120_/Bio_32_/FITC_10_ for 2 h. After the beads were washed with buffer and recovered by centrifugation, both samples were analyzed by fluorescence microscopy (Figure 6B). While SA–agarose beads were fluorescently stained green (FITC) by the dendrimer, no fluorescence was detected in the control, confirming the ability of biotin on the surface of 3­[G4]-Man_120_/Bio_32_/FITC_10_ to selectively recognize SA.

The ability of mannose on 3­[G4]-Man_120_/Bio_32_/FITC_10_ to recognize its natural receptors was assessed using a turbidimetric assay (Figure 6C). Concanavalin A (Con A) is a lectin with high affinity for α-mannose which exhibits a dimer–tetramer equilibrium above pH 5.5.^51^ As both 3­[G4]-Man_120_/Bio_32_/FITC_10_ and Con A are multivalent, the mixing of the two species is expected to result in aggregation, confirming their selective recognition. Indeed, when a solution of the dendrimer (1.67 mM of mannose) was titrated with increasing concentrations of Con A and the interaction was monitored by absorbance at 700 nm, steady increases in absorbance were observed until the concentration of Con A reached a value of 4.86 μM. At this point, the concentration of mannose was 1.19 mM. Further additions of Con A did not produce variations in absorbance. In accordance with the reversible nature of the interaction, the subsequent addition of a saturated solution of α-methyl-d-mannopyranoside completely removed turbidity, with the mixture recovering the original absorbance value, indicating complete disaggregation. Interestingly, when the experiment was performed under identical conditions with lysozyme as a protein control, no aggregation was observed, consistent with the lack of mannose recognition.

Encouraged by these results confirming that mannose and biotin on the surface of 3­[G4]-Man_120_/Bio_32_/FITC_10_ are selectively recognized by specific protein receptors and that the interaction can be monitored by the fluorescence of FITC, an in vitro experiment in A549 cells was planned to evaluate the ability of the dendrimer to act as a nanocarrier for cell internalization. While carbohydrate-coated dendrimers are recognized to efficiently internalize cells,^52,53^ peripheral biotins could be exploited as chemical handles to complex biotinylated cargoes using SA as a linker. A fluorescently labeled model cargo was chosen to evaluate the selective dendrimer-mediated cell internalization by colocalization of the cargo and dendrimer (FITC) fluorescent signals (Figure 6D).

The effective cell internalization of multifunctional 3­[G4]-Man_120_/Bio_32_/FITC_10_ by endocytosis in A549 cells was confirmed by LSCM experiments. After 2 h of incubation, colocalization of the dendrimer (FITC, green) was observed with LysoTracker Red (red), a well-known endosome–lysosome marker (Figure 6D). Then, experiments were done to test the ability of the dendrimer to internalize biotinylated cargoes. For this purpose, sulfo-Cy5-PEG_3_-biotin (Bio-Cy5), a Cy5 fluorescently labeled biotin unable to internalize A549 cells on its own, was chosen (Figure 6D). Notably, when a complex of 3­[G4]-Man_120_/Bio_32_/FITC_10_, SA, and Bio-Cy5 (dendrimer–SA–Bio-Cy5 complex, prepared in a 1:4:4 molar ratio for 1 h in phosphate-buffered saline) was incubated with A549 cells for 2 h, not only the distribution of green fluorescence (FITC of the dendrimer) was identical to that observed with the dendrimer alone but also colocalization of FITC (green) with Bio-Cy5 (magenta) and LysoTracker Red (red) confirmed the successful intracellular delivery of Bio-Cy5 mediated by the dendrimer (Figure 6D). Interestingly, when internalization experiments were performed under identical conditions with a mixture of the dendrimer and Bio-Cy5 (1:4 molar ratio) or an SA–Bio-Cy5 complex (1:1 molar ratio), no intracellular Cy5 fluorescence was observed (Figure S3), confirming that cell internalization of Bio-Cy5 exclusively proceeds mediated by the dendrimer via complexation through tetrameric SA. Overall, 3­[G4]-Man_120_/Bio_32_/FITC_10_ is revealed as an efficient nanocarrier for cell internalization of biotinylated cargoes. Application of this strategy to ligands other than mannose, biotin, and FITC is expected to provide multifunctional dendrimers with a wide range of applications in therapy and diagnosis.

## Conclusions

The azide–alkyne cycloaddition (AAC) with alkynyl dicarbamates (Alk-R) derived from 2-butyne-1,4-diol is revealed as a flexible, reliable, atom-economical, and user-friendly strategy for the multivalent functionalization of polymeric scaffolds. Alk-R functionalized with alcohols, cationic and anionic groups, biologically relevant ligands, fluorescent probes, and metal-chelating agents have readily been prepared in excellent yields, and their utility demonstrated for the efficient multivalent AAC functionalization of azide-bearing dendrimers (several generations) and block copolymers. As a result, a collection of polymers with variable functionality and double multivalency has been obtained as a platform for the development of nanostructures with promising applications in drug delivery. Examples include amphiphilic block copolymer micelles that efficiently encapsulate the anticancer drug doxorubicin (DOX) and modify its cell internalization and trafficking pathway and the development of a multifunctional (mannose, biotin, FITC) dendritic nanocarrier for selective cell internalization of biotinylated cargoes. The extension of this strategy to other ligands and polymeric scaffolds is expected to provide bioinspired functional supramolecular systems with a wide range of therapeutic and diagnostic opportunities.

## Supplementary Material



## Notes

Materials, instrumentation, experimental procedures, and characterization (PDF)
