# Supplementary material for: Bioactive Polymeric Scaffolds: Multivalent Functionalization by Thermal Azide–Alkyne Cycloaddition with Alkynyl Dicarbamates
Source: Biomacromolecules. 2025 Mar 26;26(4):2553–64. doi: 10.1021/acs.biomac.5c00038 (PMC12124718; doi:10.1021/acs.biomac.5c00038)
Supplement: Supplementary file 1 [file bm5c00038_si_001.pdf]

Supporting Information for:

Bioactive Polymeric Scaffolds: Multivalent Functionalization by Thermal Azide-Alkyne Cycloaddition with Alkynyl Dicarbamates

*Maun H. Tawara, Juan Correa, Emma Leire, Bruno Delgado Gonzalez, Samuel Parcero-Bouzas, Flonja Liko, and Eduardo Fernandez-Megia**

Centro Singular de Investigación en Química Biolóxica e Materiais Moleculares (CiQUS), Departamento de Química Orgánica, Universidade de Santiago de Compostela, Jenaro de la Fuente s/n, 15782 Santiago de Compostela, Spain.

Table of Contents

1. Synthesis of Functionalized Internal Alkynes (Alk-R) S3

2. Multivalent AAC Functionalization of Polymeric Scaffolds S24

3. DLS Size Distributions S53

4. GPC Elugrams S54

5. Preparation of PEG-[G3]-Cat Micelles and Encapsulation of DOX S55

6. Determination of the Bioactivity of Biotin on 3[G4]-Man_{120}/Bio_{32}/$FITC_{10}$ by Staining of Agarose Beads Functionalized with Streptavidin (SA) S55

7. Determination of the Bioactivity of Mannose on 3[G4]-Man_{120}/Bio_{32}/$FITC_{10}$ by Aggregation with Concanavalin A (Con A) S56

8. Cell Cultures and Cell Studies S57

9. References S60

1. Synthesis of Functionalized Internal Alkynes (Alk-R)

Alk-NHS. 2-Butyne-1,4-diol (1.0 g, 11.6 mmol) was dissolved in dry CH_3CN (36 mL). DSC (7.52 g, 29 mmol) and dry pyridine (2.4 mL, 29 mmol) were added, and the reaction was stirred under Ar at rt overnight. After evaporation under reduced pressure, the crude product was dissolved in EtOAc (90 mL) and washed with 1 M HCl (2 x 60 mL), H_2O (3 x 60 mL), and brine (50 mL). The organic phase was dried (MgSO4) and concentrated to afford Alk-NHS as a white solid (3.92 g, 92%). ^1H NMR (300 MHz, DMSO-d_6) δ: 5.22 (s, 4H), 2.81 (s, 8H). ^{13}C NMR (75 MHz, DMSO-d_6) δ: 169.8, 150.9, 81.6, 58.4, 25.4. ESI-HRMS *m/z*: 391.0492. Calcd. for $[M+Na]^+$ $C_{14}H_{12}N_2O_{10}Na$: 391.0384. IR (KBr): 1793, 1788, 1730, 1197, 1092 cm^{-1}.

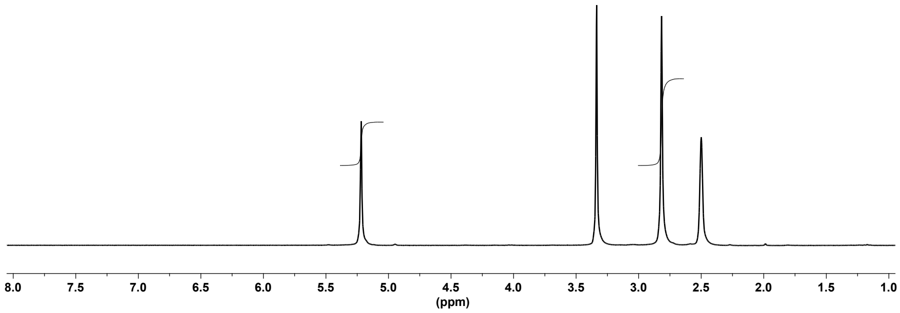

^1H NMR spectrum of Alk-NHS

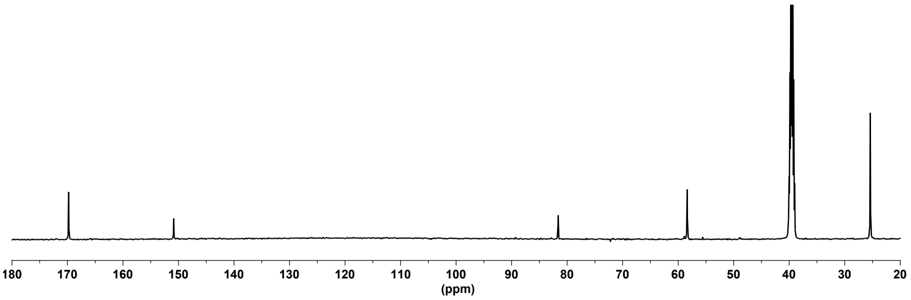

^{13}C NMR spectrum of Alk-NHS

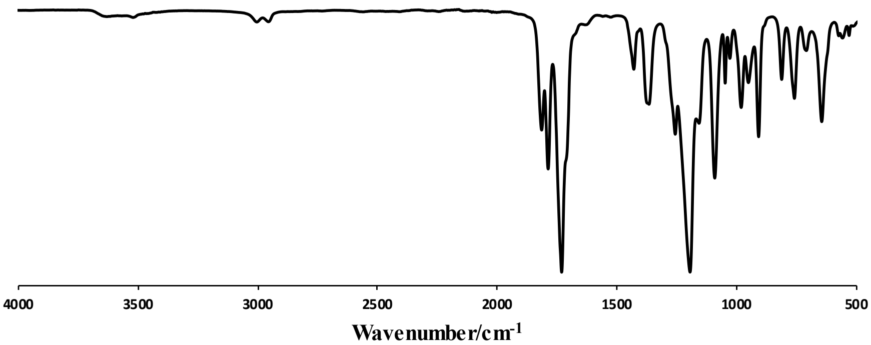

IR spectrum of Alk-NHS

Alk-OH. Ethanolamine (329 μL, 5.44 mmol) was added to a solution of Alk-NHS (500 mg, 1.36 mmol) in dry CH_3CN (6.2 mL) at 0 °C under Ar. The reaction was stirred at rt for 4 h. The solvent was evaporated under reduced pressure and the crude product was purified by automated MPLC (gradient from CH_2Cl_2 to 10% MeOH/CH_2Cl_2, silica gel, 20 min) to afford Alk-OH as a white solid (340 mg, 96%). ^{1}H NMR (300 MHz, CD_3OD) δ: 4.71 (s, 4H), 3.60 (t, J = 5.8 Hz, 4H), 3.24 (t, J = 5.8 Hz, 4H). ^{13}C NMR (75 MHz, CD_3OD) δ: 158.2, 82.1, 62.0, 53.2, 44.3. Elem. Anal. Found: C, 45.87; H, 6.03; N, 10.92, Calcd. for $C_{10}H_{16}N_2O_6$: C, 46.15; H, 6.20; N, 10.76. IR (KBr): 3330, 2933, 1694, 1517, 1244, 1096 cm^{-1}.

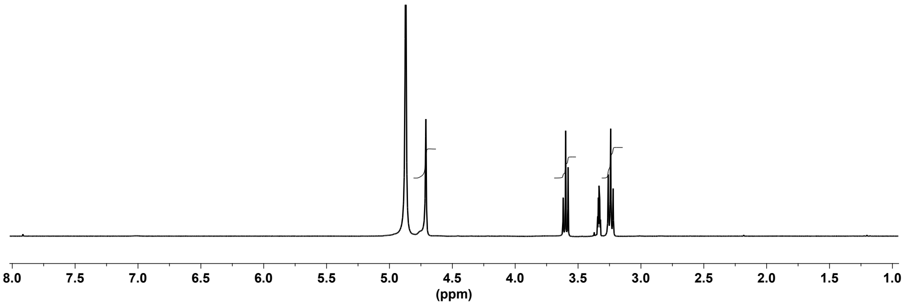

^{1}H NMR spectrum of Alk-OH

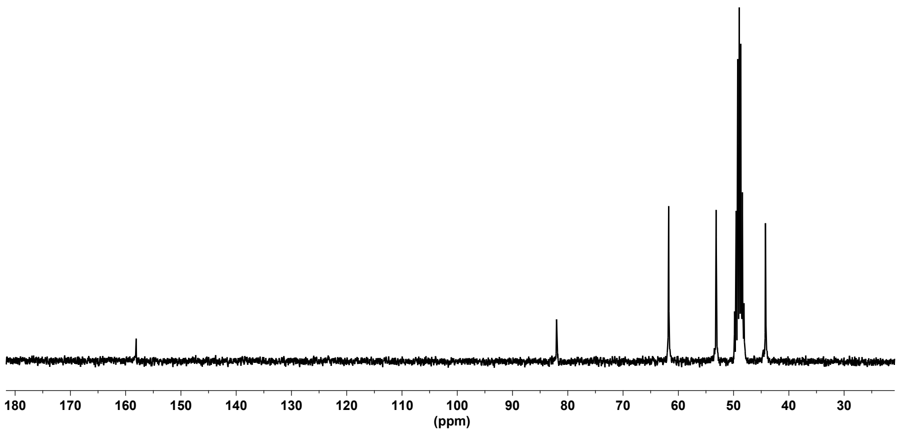

^{13}C NMR spectrum of Alk-OH

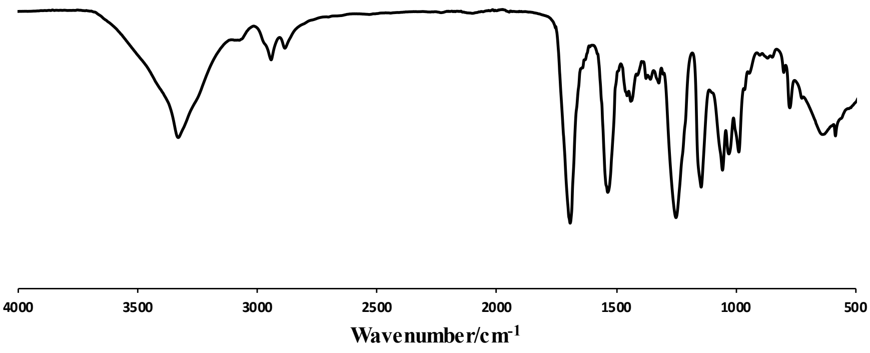

IR spectrum of Alk-OH

Alk-TEG-OH. Alk-NHS (420 mg, 1.14 mmol) was added to a solution of 2-(2-(2-aminoethoxy)ethoxy)ethanol (1.0 g, 6.83 mmol) in dry CH_2Cl_2 (5.7 mL) under Ar. The reaction was stirred at rt overnight. The solvent was evaporated under reduced pressure and the crude product was purified by automated MPLC (gradient from CH_2Cl_2 to 10% MeOH/CH_2Cl_2, silica gel, 10 min) to afford Alk-TEG-OH as a colorless oil (450 mg, 90%). ^1H NMR (300 MHz, $CDCl_3$) δ: 5.70 (br s, 2H), 4.70 (s, 4H), 3.76-3.73 (m, 4H), 3.66-3.55 (m, 16H), 3.37 (t, J = 4.9 Hz, 4H). ^{13}C NMR (75 MHz, $CDCl_3$) δ: 155.7, 81.1, 72.6, 70.7, 70.2, 69.9, 61.4, 52.5, 40.8. ESI-HRMS *m/z*: 459.1950. Calcd. for $[M+Na]^+$ $C_{18}H_{32}N_2NaO_{10}Na$: 459.1949. IR (neat, ATR): 3331, 2875, 1703, 1536, 1252, 1098 cm^{-1}.

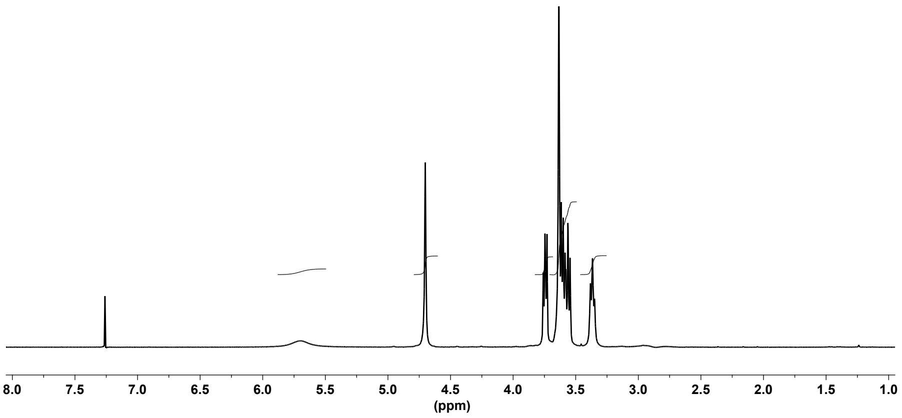

^{1}H NMR spectrum of Alk-TEG-OH

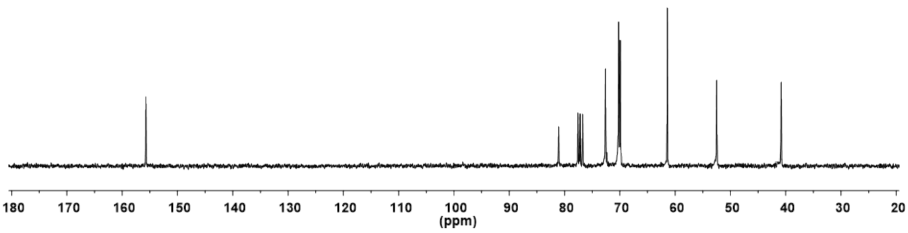

^{13}C NMR spectrum of Alk-TEG-OH

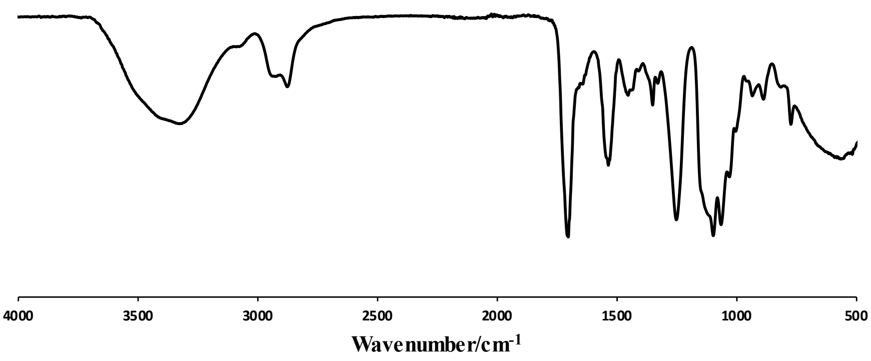

IR spectrum of Alk-TEG-OH

Alk-TEG-NHBoc. *Tert*-butyl (2-(2-(2-aminoethoxy)ethoxy)ethyl)carbamate (1.18 g, 4.75 mmol) and Et_3N (662 µL, 4.75 mmol) were added to a solution of Alk-NHS (0.50 g, 1.36 mmol) in dry CH_3CN (3.4 mL). The reaction was stirred at rt for 32 h under Ar. After evaporation under reduced pressure, the crude product was dissolved in EtOAc (50 mL) and washed with 1 M HCl (2 x 50 mL), H_2O (2 x 50 mL), and brine (50 mL). The organic phase was dried ($MgSO_4$) and concentrated to afford Alk-TEG-NHBoc as a yellow oil (848 mg, 98%). ^{1}H NMR (300 MHz, $CDCl_3$) δ: 5.41 (br s, 1H), 5.06 (br s, 1H), 4.73 (s, 4H), 3.62-3.53 (m, 16H), 3.39 (dd, J = 10.5, 5.2 Hz, 4H), 3.34-3.31 (m, 4H), 1.44 (s, 18H). ^{13}C NMR (75 MHz, $CDCl_3$) δ: 156.1, 155.6, 81.2, 79.3, 70.4, 70.2, 70.0, 52.6, 41.0, 40.4, 28.5. Elem. Anal. Found: C, 52.68; H, 8.12; N, 8.99, Calcd. for $C_{28}H_{50}N_4O_{12}$: C, 52.98; H, 7.94; N, 8.83. IR (neat, ATR): 3332, 1689, 1671, 1265, 1051 cm^{-1}.

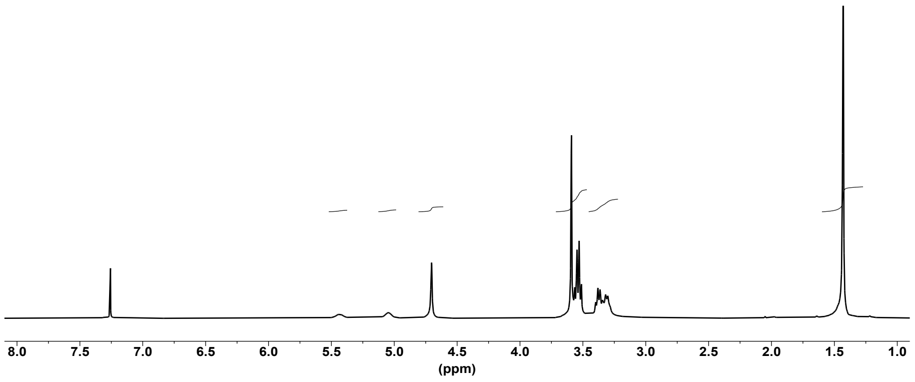

^{1}H NMR spectrum of Alk-TEG-NHBoc

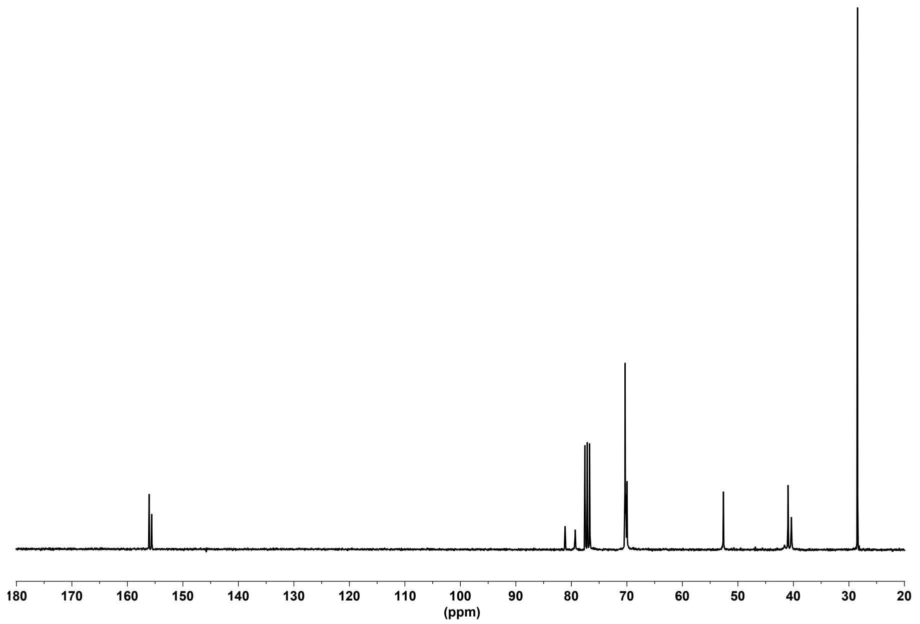

^{13}C NMR spectrum of Alk-TEG-NHBoc

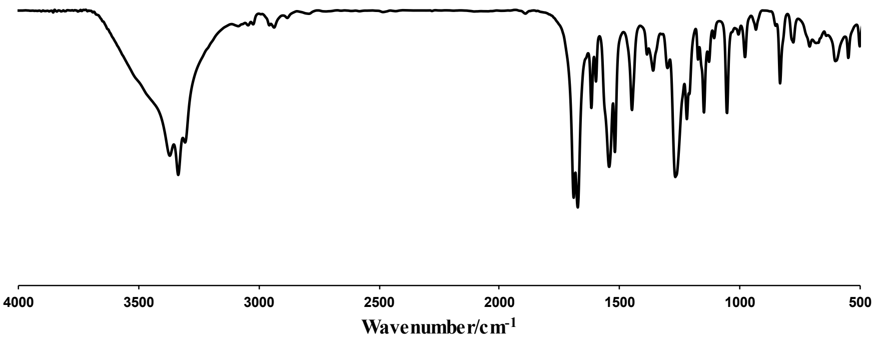

IR spectrum of Alk-TEG-NHBoc

Alk-PhOH. 4-Hydroxybenzylamine (293 mg, 2.38 mmol) was added to a solution of Alk-NHS (250 mg, 0.68 mmol) and Et_3N (331 mL, 2.38 mmol) in dry DMF (3.4 mL). The reaction was stirred at rt for 32 h under Ar. Then, it was diluted with EtOAc (50 mL) and washed with 0.5 M HCl (3 x 30 mL), H_2O (5 x 30 mL), and brine (30 mL). The organic phase was dried ($MgSO_4$) and concentrated to afford Alk-PhOH as a white solid (514 mg, 97%). ^{1}H NMR (300 MHz, DMSO-d_6) δ: 9.26 (br s, 2H), 7.77 (br s, 2H), 7.04 (d, J = 7.4 Hz, 4H), 6.69 (d, J = 7.4 Hz, 4H), 4.70 (s, 4H), 4.08 (s, 4H). ^{13}C NMR (75 MHz, DMSO-d_6) δ: 156.4, 155.5, 129.7, 128.5, 115.1, 81.6, 51.7, 43.6. Elem. Anal. Found: C, 62.11; H, 5.61; N, 6.99, Calcd. for $C_{20}H_{20}N_2O_6$: C, 62.49; H, 5.24; N, 7.29. IR (KBr): 3332, 1673, 1538, 1265, 1051 cm^{-1}.

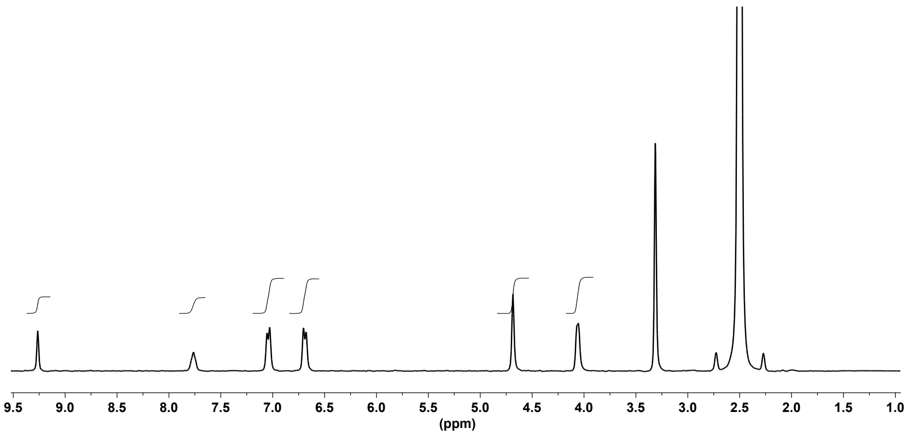

^{1}H NMR spectrum of Alk-PhOH

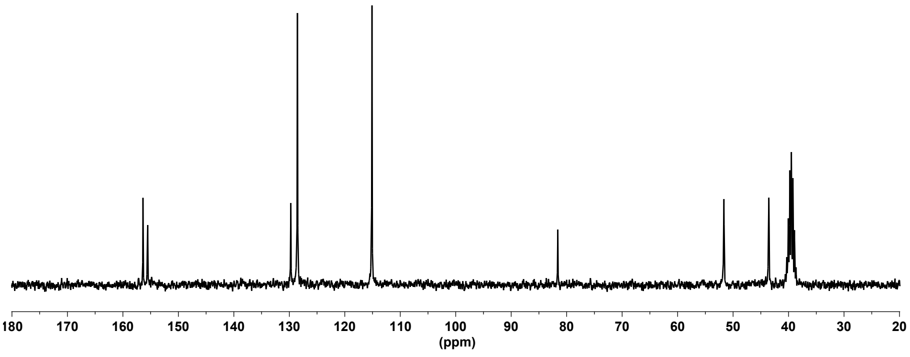

^{13}C NMR spectrum of Alk-PhOH

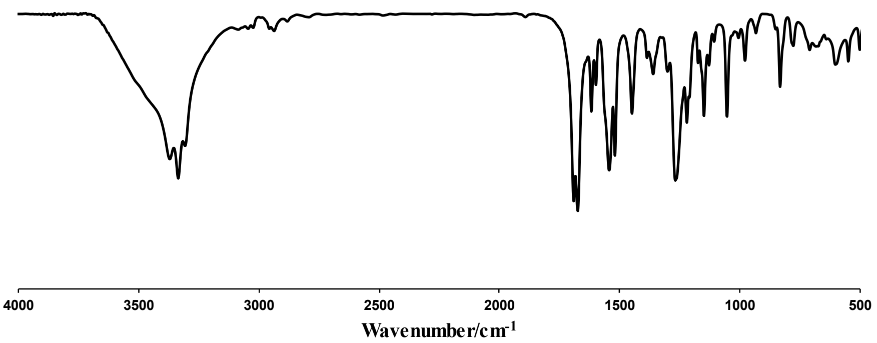

IR spectrum of Alk-PhOH

Alk-Cat. Alk-NHS (150 mg, 0.41 mmol) was added to a solution of dopamine hydrochloride (232 mg, 1.22 mmol) and Et_3N (230 µL, 1.63 mmol) in dry DMF (2.75 mL) under Ar. The reaction was stirred at rt for 24 h. After evaporation under reduced pressure, the crude product was dissolved in EtOAc (50 mL) and washed with sat $NaHCO_3$ (3 x 50 mL) and H_2O (2 x 50 mL). The organic phase was dried ($MgSO_4$) and concentrated to give a crude product that was purified by automated MPLC (gradient from hexane to EtOAc, silica gel, 30 min) to afford Alk-Cat as a yellow oil (167 mg, 92%). ^1H NMR (300 MHz, CD_3CN) δ: 6.74-6.47 (m, 6H), 5.65 (br s, 2H), 4.62 (s, 4H), 3.22 (dd, J = 13.2, 6.7 Hz, 4H), 2.59 (t, J = 7.0 Hz, 4H). ^{13}C NMR (75 MHz, CD_3CN) δ: 156.7, 145.3, 143.8, 132.4, 121.5, 116.8, 116.2, 82.1, 52.9, 43.3, 35.8. Elem. Anal. Found: C, 59.81; H, 5.23; N, 5.97, Calcd. for $C_{22}H_{24}N_2O_8$: C, 59.46; H, 5.44; N, 6.30. IR (neat, ATR): 3344, 1694, 1517, 1242 cm^{-1}.

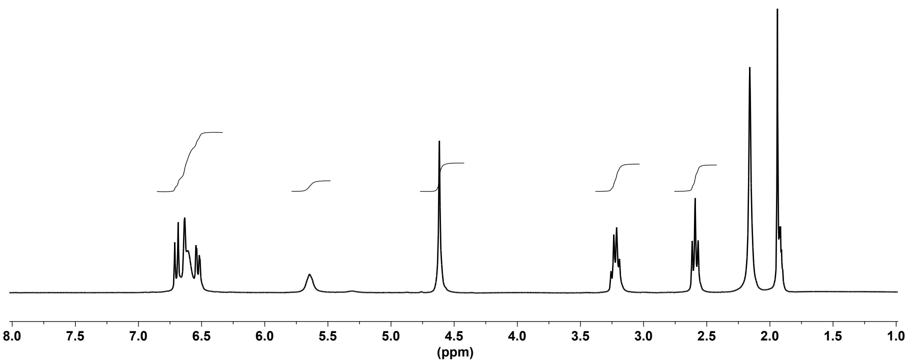

^{1}H NMR spectrum of Alk-Cat

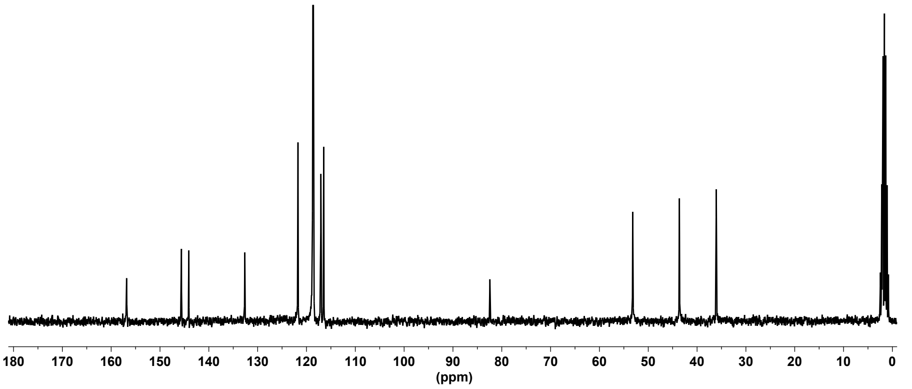

^{13}C NMR spectrum of Alk-Cat

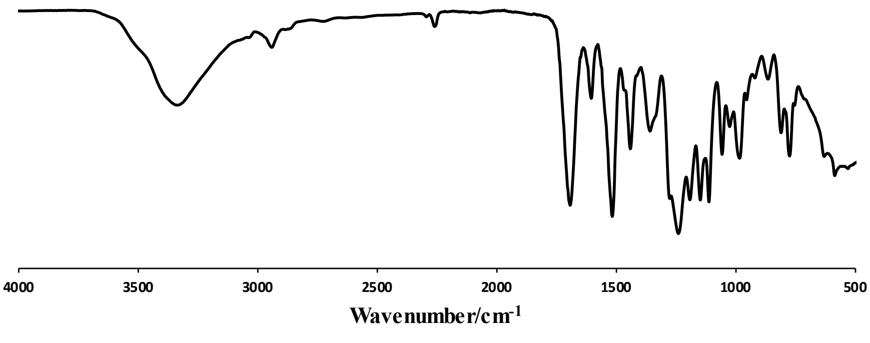

IR spectrum of Alk-Cat

Alk-OSO3H·NH3. H_2SO_4 (0.614 mL, 11.54 mmol) and Ac_2O (1.08 mL, 11.54 mmol) were added to dry pyridine (9.6 mL). After 5 min of stirring at 55 ºC, Alk-OH (250 mg, 0.961 mmol) was added. The mixture was stirred at 55 ºC for 16 h and then cooled down to 0 ºC. Ammonium hydroxide (3.5 mL, 25%) was added and after 15 min of stirring at 0 ºC, the reaction mixture was concentrated under reduced pressure and lyophilized. Sublimation of ammonium acetate (40 ºC, 0.1 mmHg) afforded Alk-OSO3H·NH3 as a white solid (359 mg, 89%). ^{1}H NMR (300 MHz, CD_3OD) δ: 4.62 (s, 4H), 3.95 (t, J = 5.5 Hz, 4H), 3.31 (t, J = 5.5 Hz, 4H). ^{13}C NMR (75 MHz, CD_3OD) δ: 157.8, 81.8, 69.5, 52.9, 44.0. Elem. Anal. Found: C, 26.06; H, 5.27; N, 12.05, Calcd. for $C_{10}H_{22}N_4O_{12}S_2$: C, 26.43; H, 4.88; N, 12.33. IR (KBr): 3341, 2979, 1701, 1364, 1067 cm^{-1}.

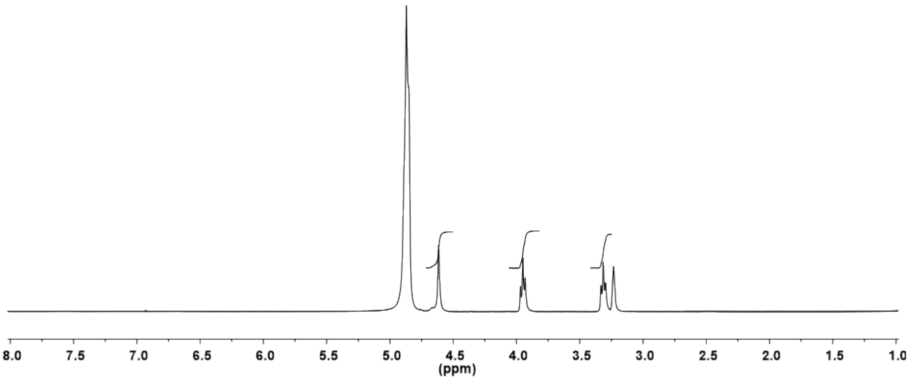

^{1}H NMR spectrum of Alk-OSO3H·NH3

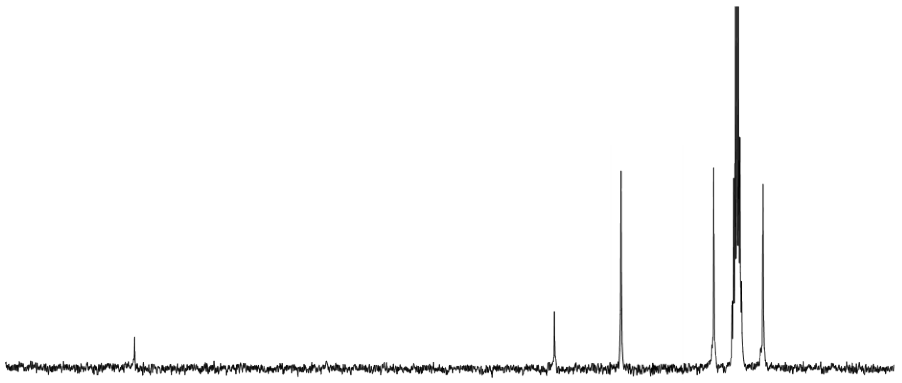

^{13}C NMR spectrum of Alk-OSO3H·NH3

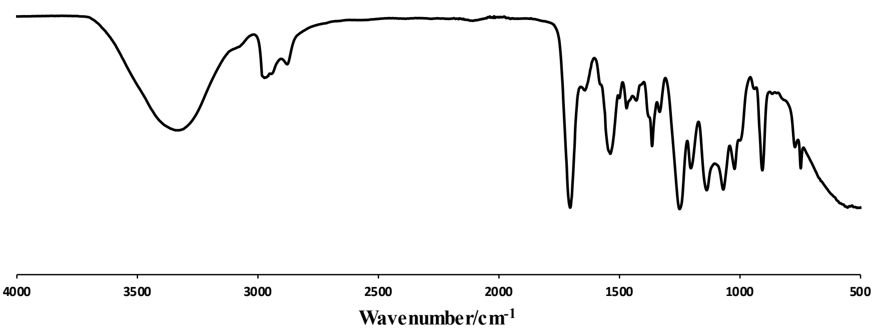

IR spectrum of Alk-$OSO_3H{\cdot}NH_3$

Alk-Man-OAc. Alk-TEG-OH (330 mg, 0.76 mmol) and 2,3,4,6-tetra-*O*-acetyl-α-D-mannopyranosyl trichloroacetimidate (1.5 g, 3.04 mmol) were dissolved in dry CH_2Cl_2 (30 mL) and cooled down to 0 °C under Ar. Dry 4Å molecular sieves were added and the mixture was stirred at 0 °C for 20 min. Then, $BF_3{\cdot}Et_2O$ (380 µL, 0.31 mmol) was added dropwise and the reaction was stirred at 0 °C for 1 h and at rt for 4 h. After addition of Et_3N (150 µL), the solvent was evaporated under reduced pressure and the crude product was purified by automated MPLC (gradient from hexane to 60% EtOAc/hexane, neutral alumina, 30 min) to give Alk-Man-OAc as an off-white solid (683 mg, 82%). ^1H NMR (750 MHz, $CDCl_3$) δ: 5.43 (t, *J* = 5.1 Hz, 2H), 5.31 (dd, *J* = 10, 3.5 Hz, 2H), 5.26 (d, *J* = 9.9 Hz, 2H), 5.22 (dd, *J* = 3.4, 1.7 Hz, 2H), 4.85 (d, *J* = 1.7 Hz, 2H), 4.67 (s, 4H), 4.24 (dd, *J* = 12.2, 5.0 Hz, 2H), 4.07 (dd, *J* = 12.2, 2.4 Hz, 2H), 4.04-4.01 (m, 2H), 3.80 (d, *J* = 2.9 Hz, 2H), 3.78 (d, *J* = 1.9 Hz, 2H), 3.67-3.57 (m, 12H), 3.52 (t, *J* = 5.2 Hz, 4H), 3.35 (dd, *J* = 10.2, 4.9 Hz, 4H), 2.12 (s, 6H), 2.07 (s, 6H), 2.01 (s, 6H), 1.96 (s, 6H). ^{13}C NMR (125 MHz, $CDCl_3$) δ: 170.7, 170.0, 169.9, 169.7, 155.5, 97.7, 81.1, 70.6, 70.3, 70.0, 69.9, 69.6, 69.0, 68.4, 67.4, 66.1, 62.4, 52.5, 40.9, 20.9, 20.73, 20.67, 20.66. ESI-HRMS *m/z*: 1097.4035. Calcd. for $[M+H]^+$ $C_{46}H_{69}N_2O_{28}$: 1097.4037. IR (MeOH, CsI): 1739, 1368, 1217, 1043 cm^{-1}.

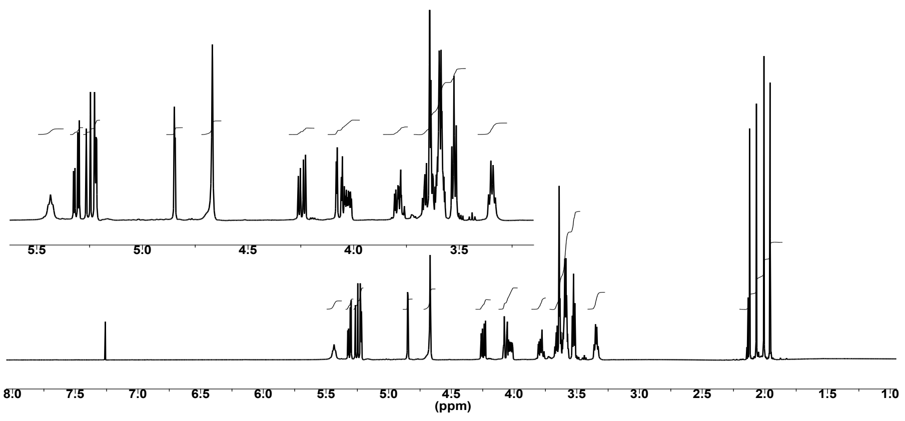

^{1}H NMR spectrum of Alk-Man-OAc

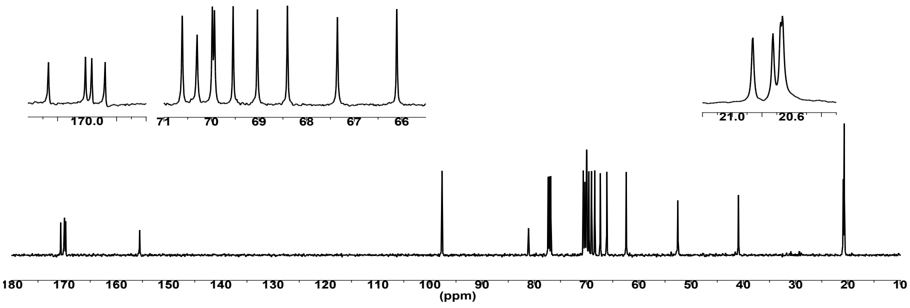

^{13}C NMR spectrum of Alk-Man-OAc

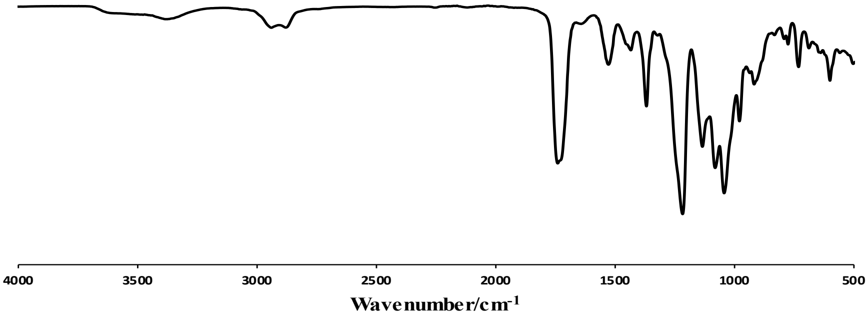

IR spectrum of Alk-Man-OAc

Alk-Man. Alk-Man-OAc (432 mg, 0.4 mmol) was dissolved in dry MeOH (60 mL). A solution of NaOMe (1 M in dry MeOH) was added until pH 9 and the reaction was stirred at rt for 1 h. Then, Amberlite IR-120 was added until neutral pH. The mixture was filtered, and the solvent was evaporated. Alk-Man was obtained as a white foam after lyophilization (301 mg, 97 %). ^{1}H NMR (750 MHz, D_2O) δ: 4.9 (d, *J* = 1.7 Hz, 2H), 4.73 (s, 4H), 3.98 (dd, *J* = 3.4, 1.7 Hz, 2H), 3.91-3.87 (m, 4H), 3.86-3.81 (m, 2H), 3.78 (dd, *J* = 4.3, 1.3 Hz, 2H), 3.77-3.74 (m, 4H), 3.74-3.68 (m, 12H), 3.67 (dd, *J* = 4.2, 2.1 Hz, 2H), 3.64 (t, *J* = 5.4 Hz, 4H), 3.36 (t, *J* = 4.7 Hz, 4H). ^{13}C NMR (125 MHz, D_2O) δ: 157.5, 99.9, 81.3, 72.7, 70.5, 70.0, 69.6, 69.5, 69.4, 69.2, 66.7, 66.3, 60.9, 52.8, 40.1. ESI-HRMS *m/z*: 783.3009. Calcd. for $[M+Na]^+$ $C_{30}H_{52}N_2NaO_{20}$: 783.3011. IR (MeOH, CsI): 3342, 2928, 1706, 1255, 1057 cm^{-1}.

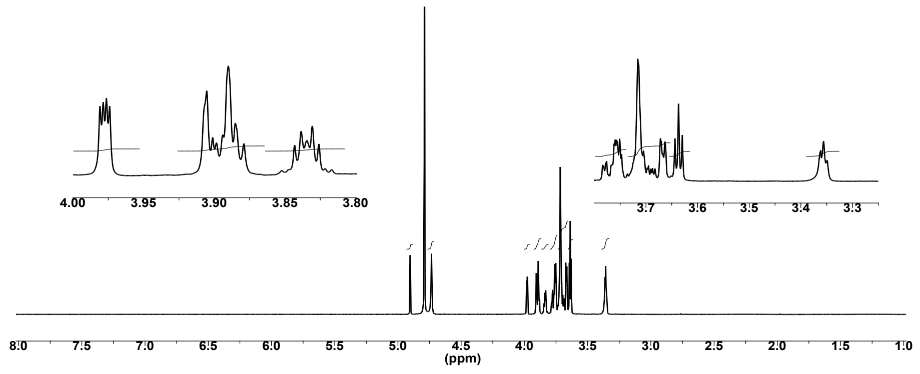

^{1}H NMR spectrum of Alk-Man

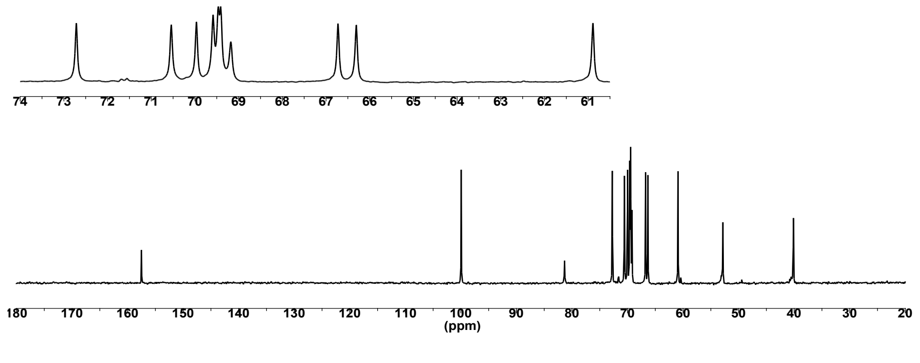

^{13}C NMR spectrum of Alk-Man

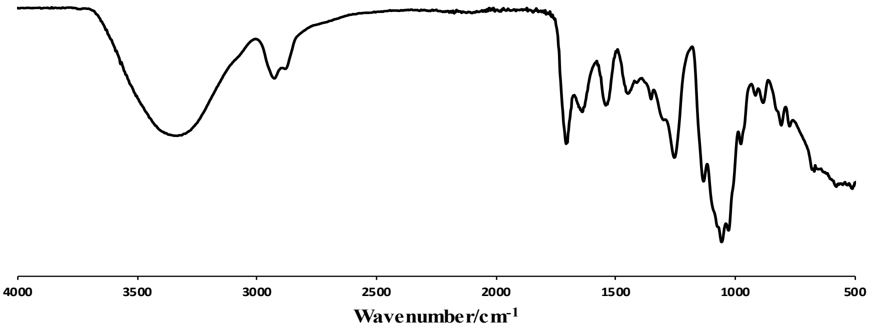

IR spectrum of Alk-Man

Alk-Glc-OAc. Alk-TEG-OH (110 mg, 0.26 mmol) and 2,3,4,6-tetra-*O*-acetyl-β-D-glucopyranosyl trichloroacetimidate (0.51 g, 1.04 mmol) were dissolved in dry CH_2Cl_2 (10 mL) and cooled down to 0 °C under Ar. Dry 4Å molecular sieves were added and the mixture was stirred at 0 °C for 20 min. Then, $BF_3{\cdot}Et_2O$ (130 µL, 0.10 mmol) was added dropwise and the reaction was stirred at 0 °C for 1 h and at rt for 4 h. After addition of Et_3N (150 µL), the solvent was evaporated under reduced pressure and the crude product was purified by automated MPLC (gradient from hexane to 60% EtOAc/hexane, neutral alumina, 30 min) to give Alk-Glc-OAc as an off-white solid (210 mg, 76%). ^{1}H NMR (500 MHz, $CDCl_3$) δ: 5.42 (br s, 2H), 5.17 (t, J = 9.5 Hz, 2H), 5.05 (t, J = 9.7 Hz, 2H), 4.96 (dd, J = 9.6, 8.0 Hz, 2H), 4.69 (s, 4H), 4.59 (d, J = 8.0 Hz, 2H), 4.23 (dd, J = 12.3, 4.7 Hz, 2H), 4.11 (dd, J = 12.3, 2.4 Hz, 2H), 3.96–3.89 (m, 2H), 3.76–3.66 (m, 4H), 3.65–3.54 (m, 10H), 3.52 (t, J = 5.2 Hz, 4H), 3.35 (dd, J = 10.6, 5.4 Hz, 4H), 2.06 (s, 6H), 2.02 (s, 6H), 1.99 (s, 6H), 1.97 (s, 6H). ^{13}C NMR (125 MHz, $CDCl_3$) δ: 170.6, 170.2, 169.4, 169.3, 155.5, 100.7, 81.1, 72.8, 71.7, 71.2, 70.6, 70.2, 69.8, 69.0, 68.4, 61.9, 52.5, 40.9, 20.66, 20.59, 20.53, 20.51. ESI-HRMS *m/z*: 1119.3913. Calcd. for $[M+Na]^+$ $C_{46}H_{68}N_2O_{28}Na$: 1119.3856. IR (MeOH, CsI): 1739, 1368, 1217, 1043 cm^{-1}.

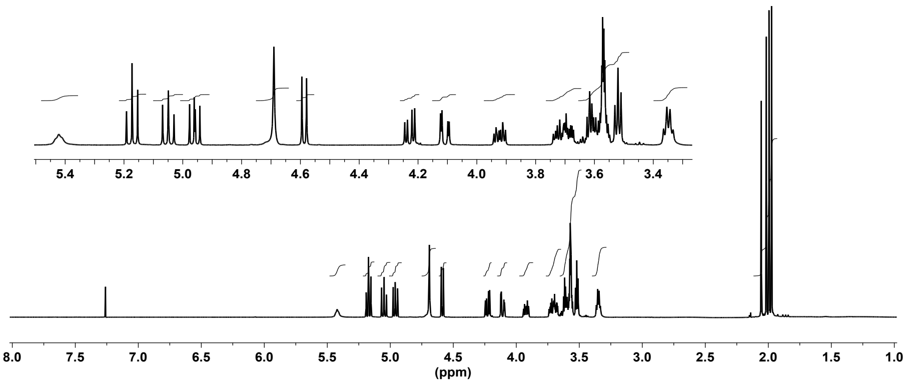

^{1}H NMR spectrum of Alk-Glc-OAc

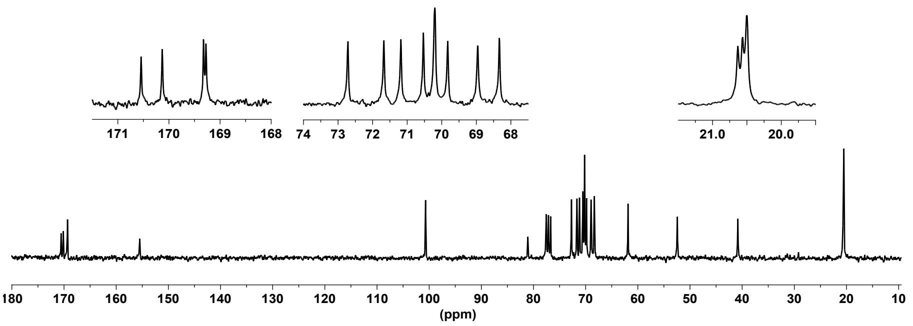

^{13}C NMR spectrum of Alk-Glc-OAc

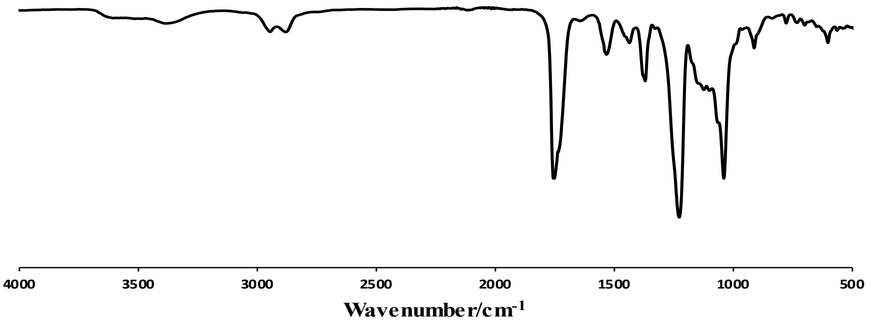

IR spectrum of Alk-Glc-OAc

Alk-Glc. Alk-Glc-OAc (410 mg, 3.7 mmol) was dissolved in dry MeOH (56 mL). A solution of NaOMe (1 M in dry MeOH) was added until pH 9 and the reaction was stirred at rt for 1 h. Then, Amberlite IR-120 was added until neutral pH. The mixture was filtered, and the solvent was evaporated. Alk-Glc was obtained as a white foam after lyophilization (281 mg, 99%). ^{1}H NMR (500 MHz, D_2O) δ: 4.73 (s, 4H), 4.50 (d, J = 7.9 Hz, 2H), 4.10-4.04 (m, 2H), 3.93 (dd, J = 12.3, 2.2 Hz, 2H), 3.89-3.81 (m, 2H), 3.79-3.66 (m, 14H), 3.63 (t, J = 5.4 Hz, 4H), 3.51 (dd, J = 9.2, 8.0 Hz, 2H), 3.48-3.44 (m, 2H), 3.40 (dd, J = 9.4, 8.0 Hz, 2H), 3.35 (t, J = 5.2 Hz, 4H), 3.31 (dd, J = 9.4, 8.0 Hz, 2H). ^{13}C NMR (75 MHz, D_2O) δ: 157.6, 102.2, 81.4, 75.9, 75.7, 73.1, 69.81, 69.79, 69.6, 69.4, 69.2, 69.0, 68.7, 60.8, 52.9, 40.1. ESI-HRMS m/z: 783.2988. Calcd. for $[M+Na]^+$ $C_{30}H_{52}N_2NaO_{20}$: 783.3006. IR (MeOH, CsI): 3369, 2921, 1708, 1257, 1038 cm^{-1}.

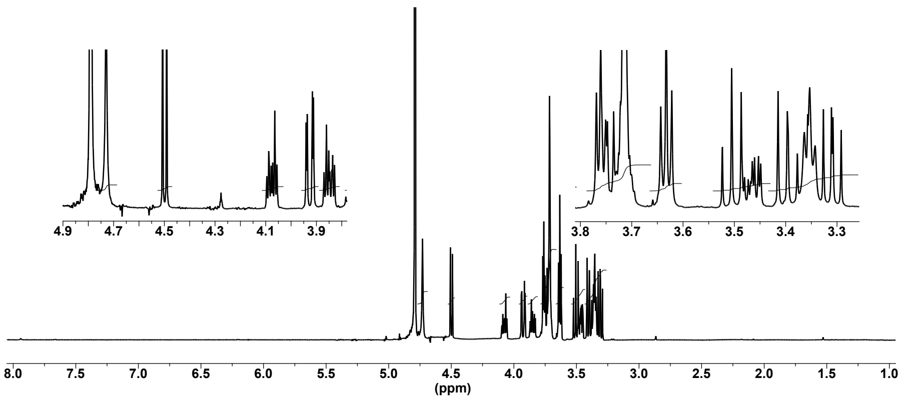

^{1}H NMR spectrum of Alk-Glc

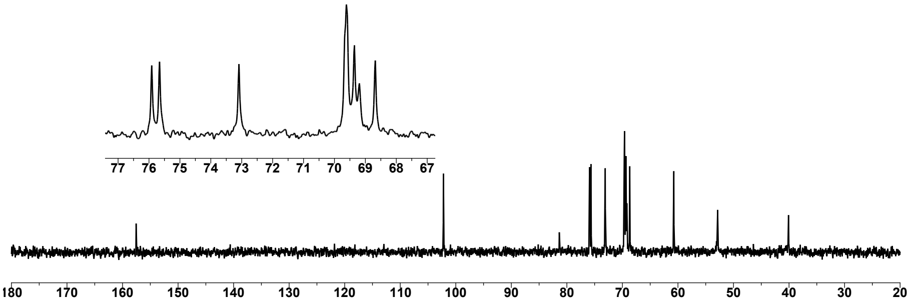

^{13}C NMR spectrum of Alk-Glc

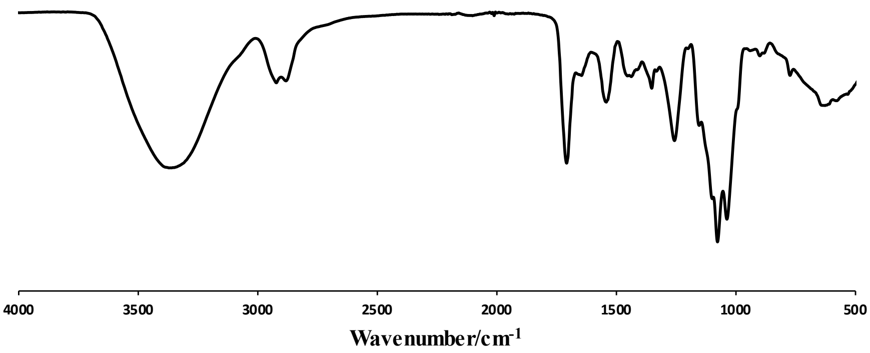

IR spectrum of Alk-Glc

Alk-TEG-NH$_2$·HCl. HCl (2.8 mL, 8.4 mmol, 3 M in MeOH) was added to a solution of Alk-TEG-NHBoc (0.553 g, 0.836 mmol) in MeOH (15 mL). The mixture was stirred for 30 min and then the solvent was evaporated. Alk-TEG-NH$_2$·HCl was obtained as a white foam after lyophilization (423 mg, 100%). ^{1}H NMR (300 MHz, D$_2$O) δ: 4.70 (s, 4H), 3.75 (t, J = 5.3 Hz, 4H), 3.70 (s, 8H), 3.62 (t, J = 5.3 Hz, 4H), 3.34 (t, J = 5.3 Hz, 4H), 3.20 (t, J = 5.3 Hz, 4H). ^{13}C NMR (75 MHz, D$_2$O) δ: 160.1, 83.9, 72.1, 72.0, 71.8, 68.9, 55.4, 42.6, 41.7. Elem. Anal. Found: C, 42.83; H, 7.41; N, 10.88, Calcd. for $C_{18}H_{36}Cl_2N_4O_8$: C, 42.61; H, 7.15; N, 11.04. IR (KBr): 2878, 1703, 1525, 1251, 1089 cm^{-1}.

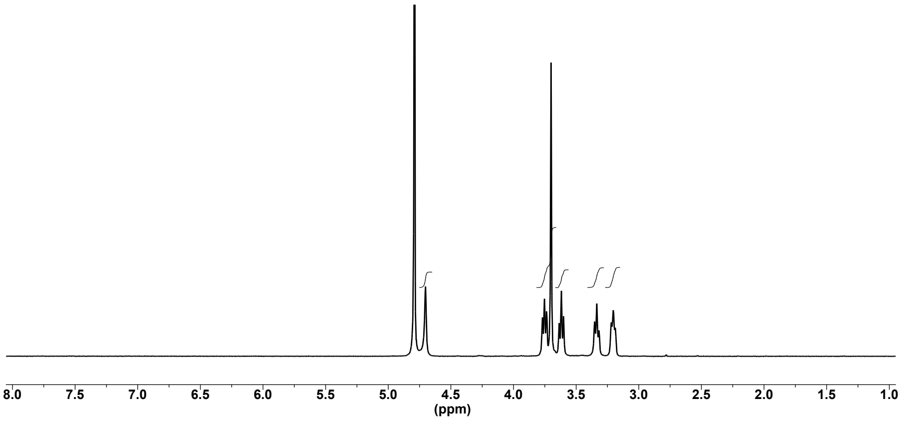

^{1}H NMR spectrum of Alk-TEG-NH$_2$·HCl

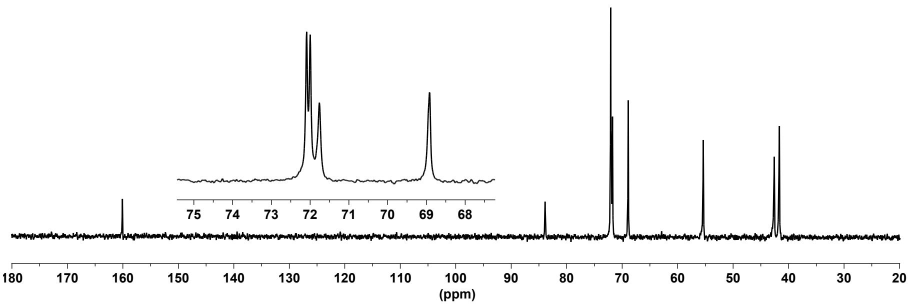

^{13}C NMR spectrum of Alk-TEG-NH_2·HCl

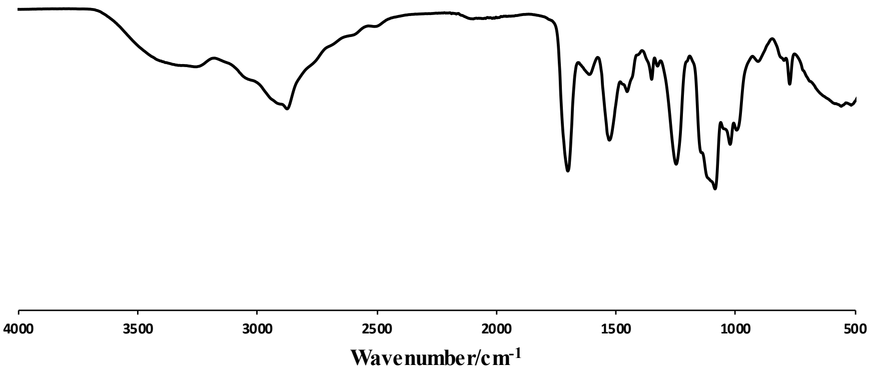

IR spectrum of Alk-TEG-NH_2·HCl

Alk-Bio. EDC·HCl (105 mg, 0.550 mmol) was added to a solution of biotin (134 mg, 0.550 mmol), Alk-TEG-NH_2·HCl (93 mg, 0.183 mmol), HOBt (74 mg, 0.550 mmol), and Et_3N (102 μL, 0.734 mmol) in dry DMF (4.6 mL) at 0 °C under Ar. The reaction mixture was stirred at 0 °C for 1.5 h and then allowed to reach rt. After 24 h of additional stirring, the solvent was evaporated, and the resulting yellow oil was purified by automated MPLC (gradient from $CHCl_3$ to 25% MeOH/$CHCl_3$, neutral alumina, 30 min) to afford Alk-Bio as a yellow oil (131 mg, 93%). ^{1}H NMR (500 MHz, diffusion filter 50 ms, CD_3OD) δ: 4.74 (s, 4H), 4.54 (dd, J = 6.4, 5.9 Hz, 2H), 4.36 (dd, J = 7.4, 4.1 Hz, 2H), 3.67 (s, 8H), 3.59 (dd, J = 5.1, 3.7 Hz, 8H), 3.42 (t, J = 5.3 Hz, 4H), 3.34 (t, J = 5.5 Hz, 4H), 3.29-3.22

(m, 2H), 2.98 (dd, J = 12.8, 4.1 Hz, 2H), 2.76 (d, J = 12.8 Hz, 2H), 2.28 (t, J = 7.2 Hz, 4H), 1.84-1.59 (m, 8H), 1.54-1.44 (m, 4H). ^{13}C NMR (125 MHz, CD_3OD) δ: 176.2, 166.0, 157.9, 82.2, 71.3, 71.2, 70.9, 70.6, 63.3, 61.6, 57.0, 53.3, 41.8, 41.1, 40.3, 36.8, 29.7, 29.5, 26.8. Elem. Anal. Found: C, 51.63; H, 6.78; N, 12.29, Calcd. for $C_{38}H_{62}N_8O_{12}S_2$: C, 51.45; H, 7.05; N, 12.63. IR (MeOH, CsI): 3281, 2934, 1693, 1645, 1257 cm^{-1}.

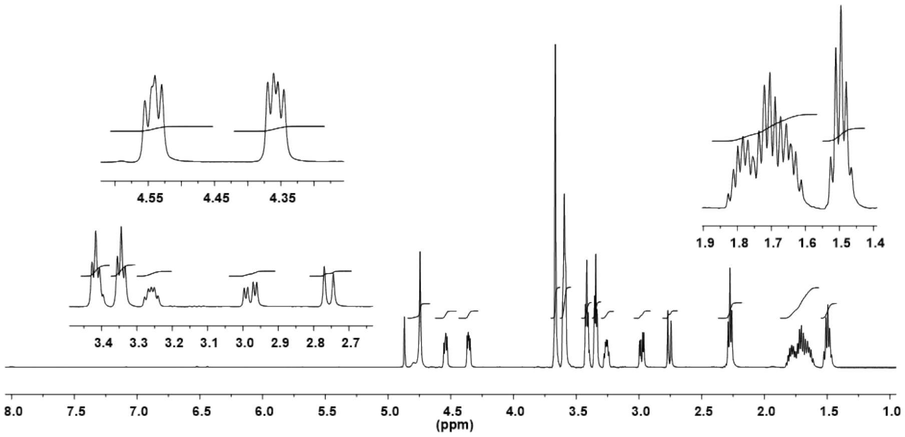

^{1}H NMR spectrum of Alk-Bio

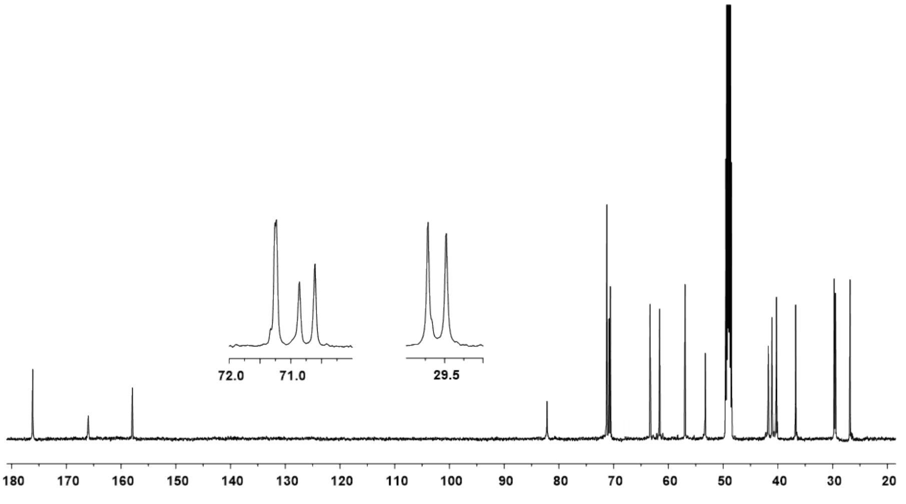

^{13}C NMR spectrum of Alk-Bio

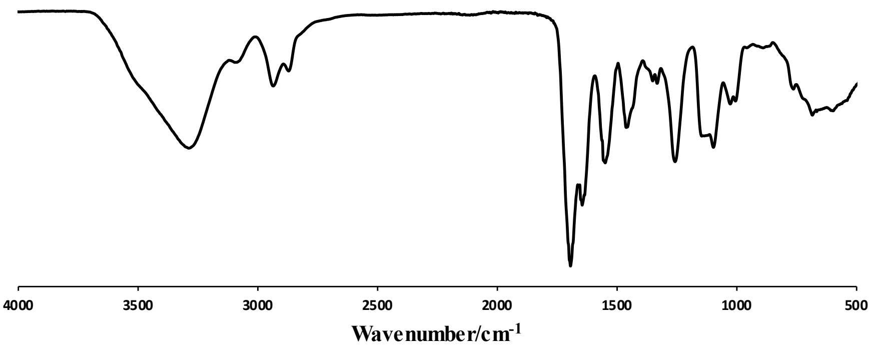

IR spectrum of Alk-Bio

Alk-FITC. Fluorescein 5-isothiocyanate (190 mg, 0.489 mmol; FITC) was added to a solution of Alk-TEG-NH_2·HCl (100 mg, 0.196 mmol) and Et_3N (109 μL, 0.783 mmol) in dry THF (3.9 mL) under Ar. After 16 h of stirring at rt in the dark, the solvent was evaporated and the resulting yellow oil was purified by automated MPLC (gradient from CH_2Cl_2 to 15% MeOH/CH_2Cl_2, neutral alumina, 30 min) to afford Alk-FITC as a yellow foam (173 mg, 73%). ^{1}H NMR (500 MHz, DMSO-d_6) δ: 10.11 (br s, 4H), 8.28 (s, 2H), 8.14 (br s, 2H), 7.72 (d, J = 11.9 Hz, 2H), 7.35 (t, J = 4.1 Hz, 2H), 7.16 (t, J = 6.3 Hz, 2H), 6.73-6.45 (m, 10H), 4.66 (s, 4H), 3.75-3.21 (m, 20H), 3.14 (dd, J = 10.6, 4.8 Hz, 4H). ^{13}C NMR (75 MHz, DMSO-d_6) δ: 181.0, 169.1, 159.8, 155.9, 152.3, 147.7, 141.7, 130.1, 129.4, 126.9, 124.4, 117.2, 113.1, 110.1, 102.7, 83.6, 81.8, 70.0, 69.9, 69.4, 68.7, 52.0, 46.3, 44.1. IR (MeOH, CsI): 3296, 2926, 1712, 1456, 1111 cm^{-1}.

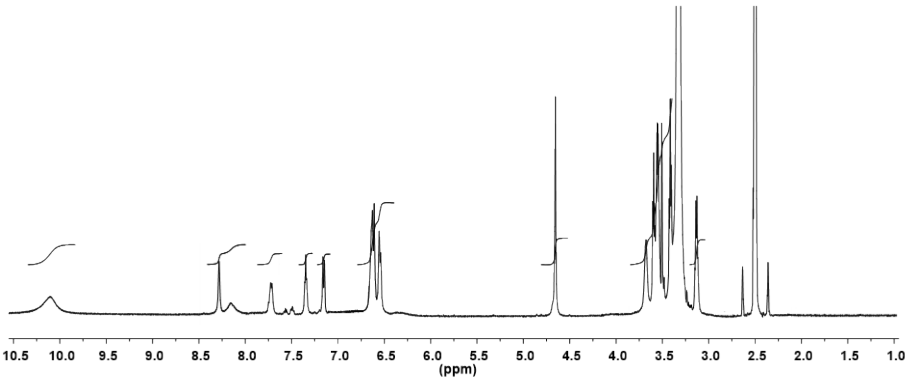

^{1}H NMR spectrum of Alk-FITC

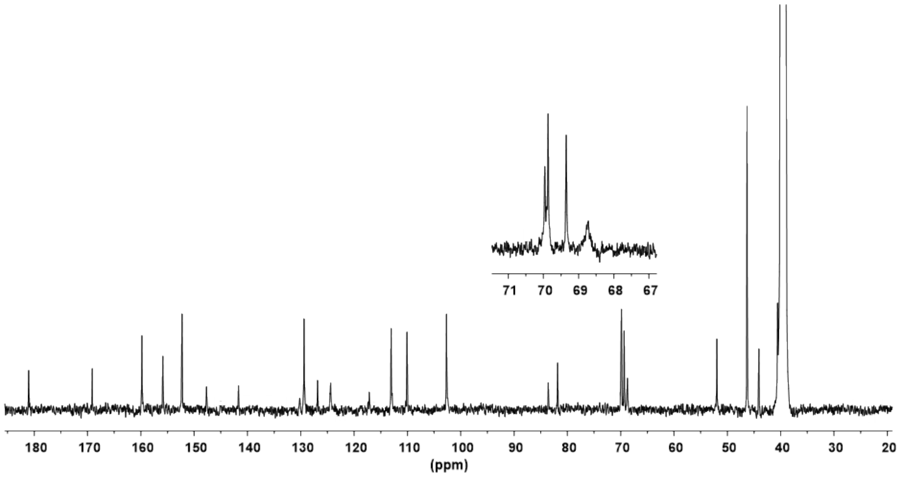

^{13}C NMR spectrum of Alk-FITC

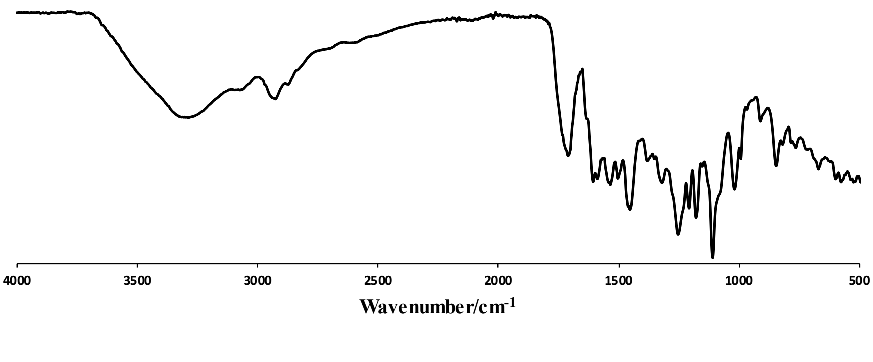

IR spectrum of Alk-FITC

2. Multivalent AAC Functionalization of Polymeric Scaffolds

Working with Azides – WARNING!

- For warnings on working with azides, readers are referred to ref 1.

- For organic azides to be manipulable or non-explosive, the "Smith's rules" must be followed: i) the number of nitrogen atoms (N_N) must not exceed that of carbon (N_C), and ii) $(N_C + N_O)/N_N \geq 3$. Similarly, the "rule of six" indicates that six carbons (or other atoms of about the same size) per azide provides sufficient dilution to render an organic azide relatively safe.[2,3] All organic azides in this report follow these rules and have proven to be stable in our hands.

3[G2]-OH. In a Schlenk flask, Alk-OH (98 mg, 377 µmol) was added to a solution of 3[G1]-N_3 (50 mg, 21 µmol) in *t*-BuOH/H_2O 5:1 (188 µL). The reaction was stirred at 120 °C for 8 h and then was purified by ultrafiltration (4 x 30 mL H_2O, Amicon YM1) and lyophilized to afford 3[G2]-OH as a white foam (92 mg, 93%). ^{1}H NMR (500 MHz, D_2O) δ: 7.10 (br s, 6H), 5.93 (br s, 3H), 5.37-5.07 (m, 36H), 4.67-4.52 (m, 18H), 4.20-4.00 (m, 24H), 3.98-3.39 (m, 138H), 3.28-3.07 (m, 36H). ^{13}C NMR (125 MHz, DMSO-d_6) δ: 165.0, 160.0, 156.1, 155.5, 151.9, 141.7, 139.9, 132.1, 129.2, 106.2, 94.9, 72.0, 70.0, 69.8, 69.5, 69.1, 68.9, 68.4, 59.9, 56.8, 52.7, 48.0, 43.1. IR (KBr): 3338, 2972, 1704, 1251, 1068 cm^{-1}. MALDI-TOF MS (HABA, linear mode, *m/z*): 4757.07. Calcd. for $[M+Na]^+$, $C_{189}H_{300}N_{48}O_{93}Na$: 4755.02.

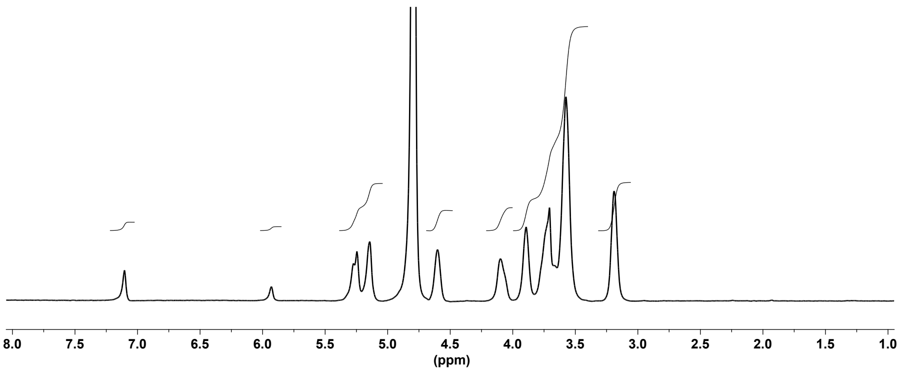

^{1}H NMR spectrum of 3[G2]-OH

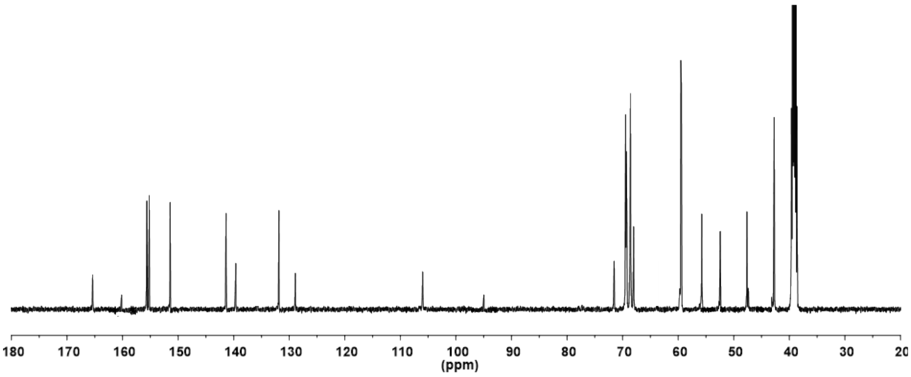

^{13}C NMR spectrum of 3[G2]-OH

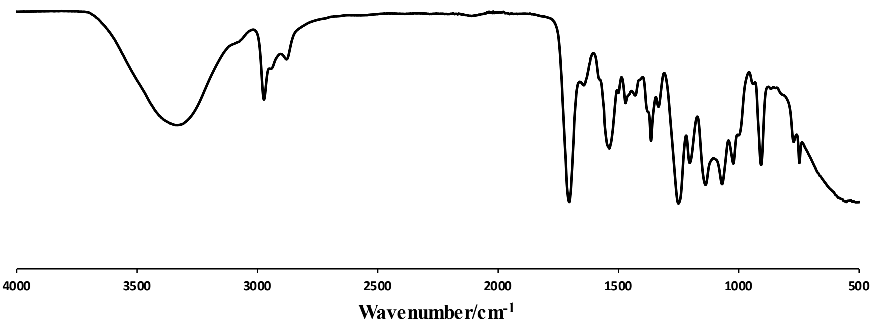

IR spectrum of 3[G2]-OH

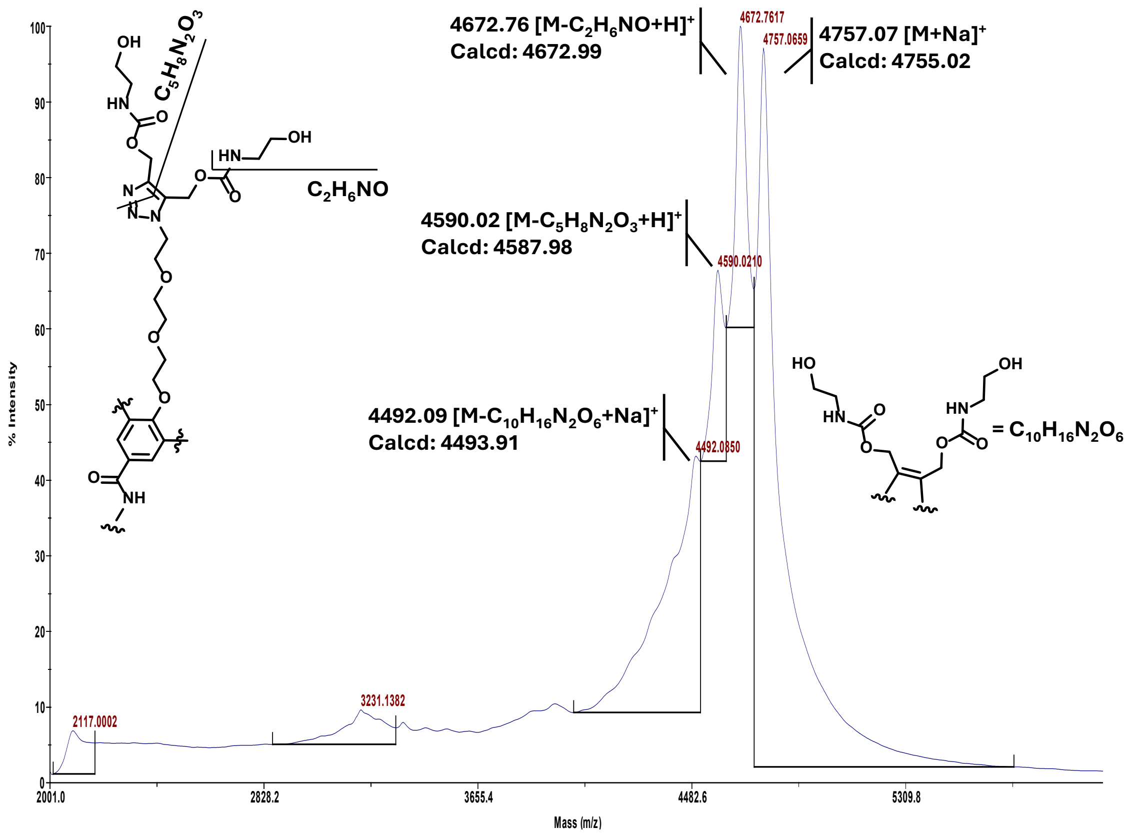

MALDI-TOF MS of 3[G2]-OH

3[G2]-TEG-OH. In a Schlenk flask, Alk-TEG-OH (165 mg, 377 µmol) was added to a solution of 3[G1]-N_3 (50 mg, 21 µmol) in *t*-BuOH/H_2O 5:1 (188 µL). The reaction was stirred at 120 °C for 8 h and then was purified by ultrafiltration (4 x 30 mL H_2O, Amicon YM1) and lyophilized to afford 3[G2]-TEG-OH as a yellow oil (119 mg, 90%). ^{1}H NMR (500 MHz, D_2O) δ: 7.14 (br s, 6H), 5.99 (br s, 3H), 5.29-5.16 (m, 36H), 4.62 (br s, 18H), 4.12-4.09 (m, 18H), 3.94-3.90 (m, 24H), 3.75-3.54 (m, 264H), 3.26 (br s, 36H). ^{13}C NMR (125 MHz, D_2O) δ: 168.6, 160.0, 157.5, 156.9, 151.9, 141.9, 139.7, 132.7, 129.2, 106.2, 94.2, 72.10, 71.7, 69.9, 69.8, 69.6, 69.4, 69.2, 69.1, 69.0, 68.9, 68.4, 67.2, 63.8, 60.3, 56.9, 53.8, 48.6, 40.1, 39.8. IR (MeOH, CsI): 3376, 2876, 1713, 1544, 1253, 1117 cm^{-1}.

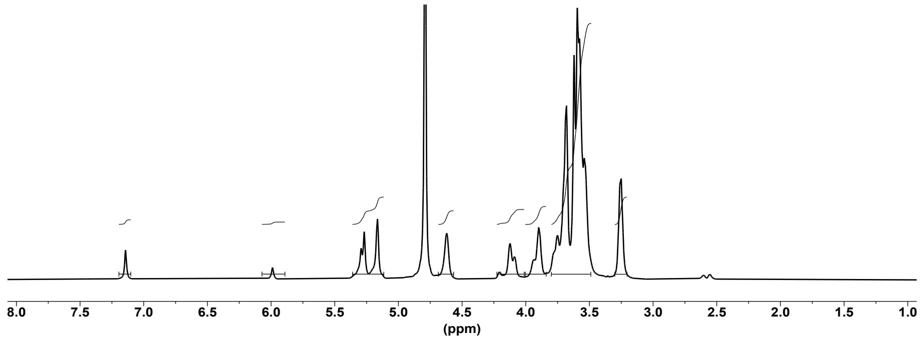

^{1}H NMR spectrum of 3[G2]-TEG-OH

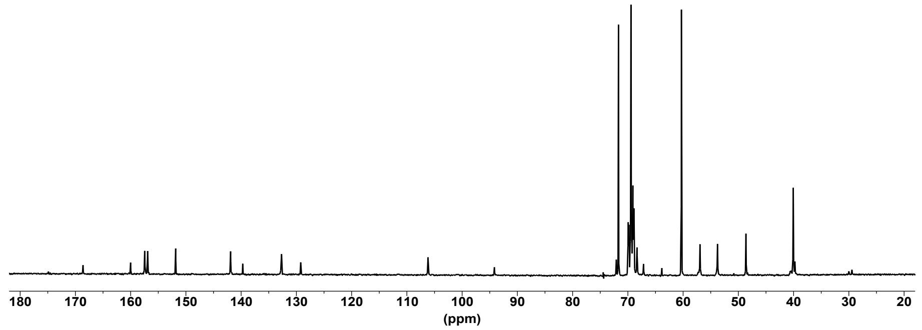

^{13}C NMR spectrum of 3[G2]-TEG-OH

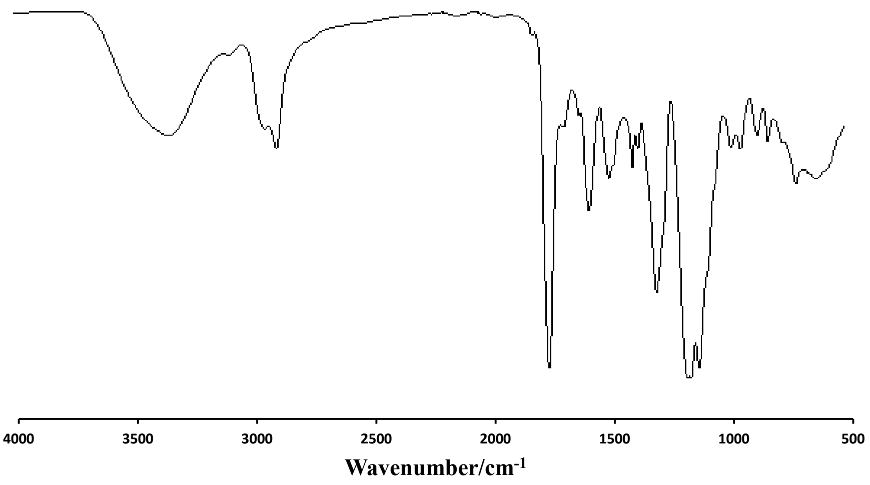

IR spectrum of 3[G2]-TEG-OH

3[G2]-Man. In a Schlenk flask, Alk-Man (131 mg, 172.8 μmol) was added to a solution of 3[G1]-N_3 (23 mg, 9.6 μmol) in *t*-BuOH/H_2O 5:1 (150 μL). The reaction was stirred at 120 ºC for 12 h and then was purified by ultrafiltration (4 x 30 mL H_2O, Amicon YM1) and lyophilized to afford 3[G2]-Man as an off-white solid (82 mg, 92%). ^{1}H NMR (500 MHz, D_2O) δ: 7.15 (br s, 6H), 6.00 (br s, 3H), 5.36-5.15 (m, 36H), 4.87 (br s, 18H), 4.69-4.57 (m, 18H), 4.19-4.04 (m, 24H), 3.96-3.54 (m, 376H), 3.33-3.18 (m, 36H). ^{13}C NMR (125 MHz, D_2O) δ: 168.7, 159.9, 157.4, 156.9, 151.8, 141.9, 139.6, 132.7, 129.2, 106.1, 99.8, 94.1, 72.7, 70.4, 69.9, 69.5, 69.4, 69.3,69.1, 69.0, 68.9, 68.6, 68.3, 67.1, 66.6, 66.2, 60.8, 56.9, 53.8, 48.6, 40.0, 39.7. IR (KBr): 3352, 2923, 1716, 1252, 1101 cm^{-1}.

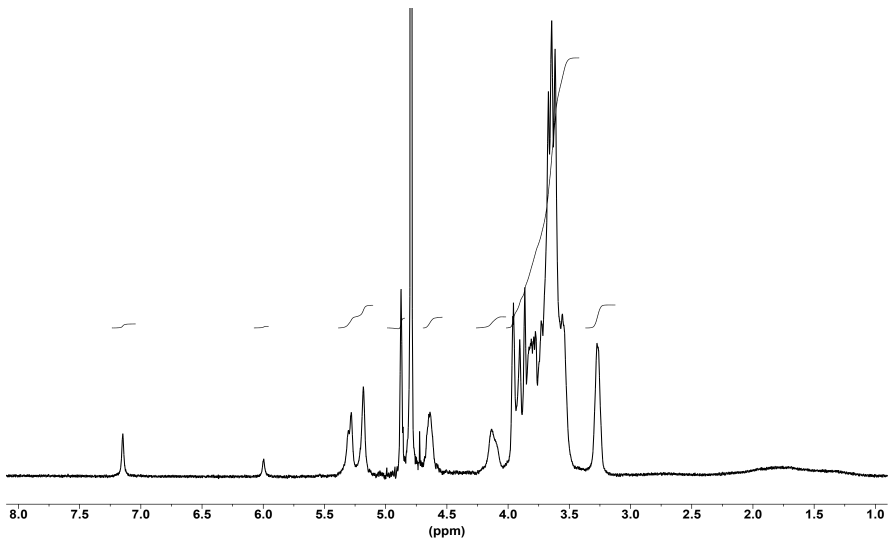

^{1}H NMR spectrum of 3[G2]-Man

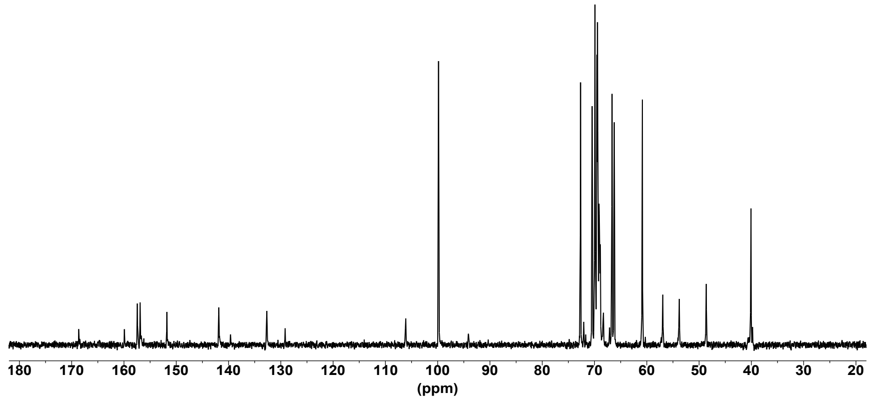

^{13}C NMR spectrum of 3[G2]-Man

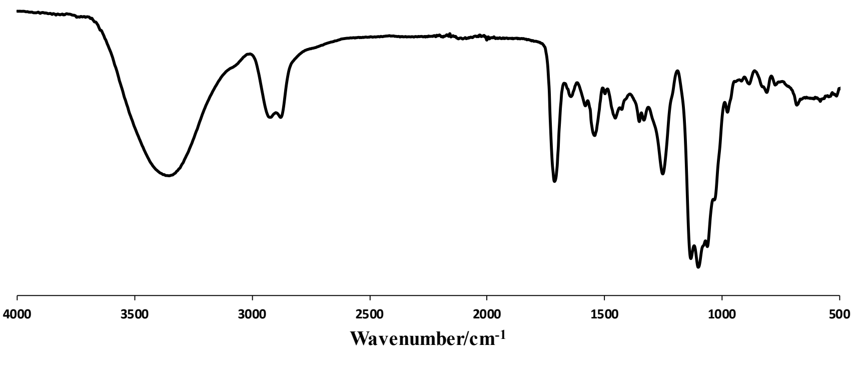

IR spectrum of 3[G2]-Man

3[G2]-Glc. In a Schlenk flask, Alk-Glc (114 mg, 151 μmol) was added to a solution of 3[G1]-N_3 (20 mg, 8.4 μmol) in *t*-BuOH/H_2O 5:1 (150 μL). The reaction was stirred at 120 ºC for 12 h and then was purified by ultrafiltration (4 x 30 mL H_2O, Amicon YM1) and lyophilized to afford 3[G2]-Glc as an off-white foam (70 mg, 91%). ^{1}H NMR (500 MHz, D_2O) δ: 7.13 (br s, 6H), 5.98 (br s, 3H), 5.37-5.07 (m, 36H), 4.68-4.56 (m, 18H), 4.54-4.39 (m, 18H), 4.20-3.98 (m, 24H), 3.97-3.33 (m, 390H), 3.32-3.12 (m, 36H). ^{13}C NMR (125 MHz, D_2O) δ: 168.7, 160.0, 157.5, 157.0, 151.9, 141.9, 139.7, 132.7, 129.2, 106.2, 102.2, 94.2, 75.9, 75.7, 73.1, 72.1, 71.7, 70.0, 69.9, 69.7, 69.4, 69.1, 68.9, 68.6, 68.4, 67.2, 60.8, 60.3, 57.0, 53.8, 48.7, 40.1. IR (KBr): 3348, 2874, 1705, 1252, 1074 cm^{-1}.

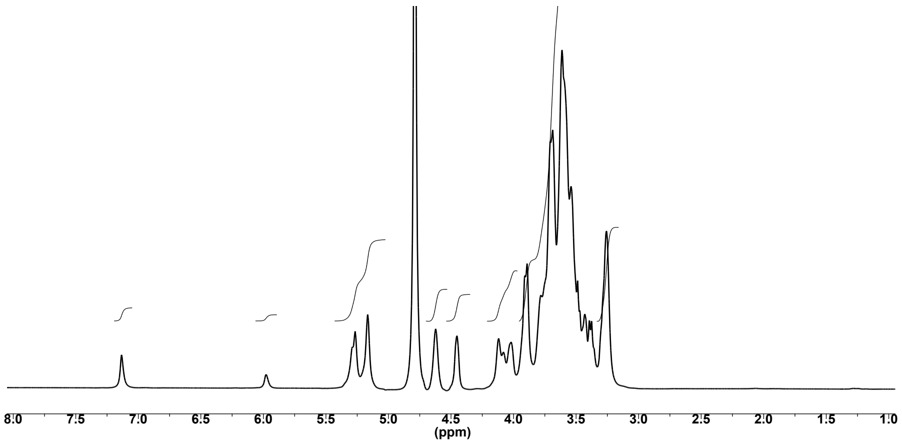

^{1}H NMR spectrum of 3[G2]-Glc

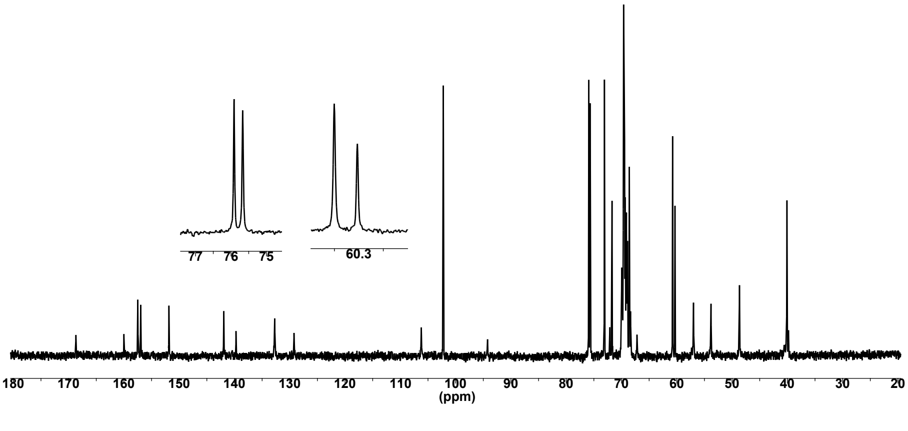

^{13}C NMR spectrum of 3[G2]-Glc

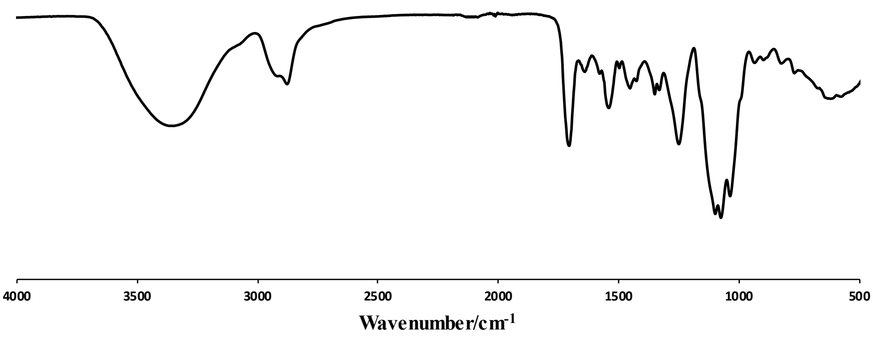

IR spectrum of 3[G2]-Glc

2[G3]-OH. In a Schlenk flask, Alk-OH (94 mg, 362 μmol) was added to a solution of 2[G2]-N_3 (50 mg, 10 μmol) in *t*-BuOH/H_2O 5:1 (181 μL). The reaction was stirred at 120 °C for 8 h and then was, purified by ultrafiltration (4 x 30 mL H_2O, Amicon YM1) and lyophilized to afford 2[G3]-OH as a white foam (91 mg, 94%). ^{1}H NMR (500 MHz, diffusion filter 50 ms, D_2O) δ: 7.21-7.01 (m, 16H), 5.39-5.09 (m, 72H), 4.68-4.56 (m, 36H), 4.19-3.96 (m, 48H), 3.95-3.47 (m, 288H), 3.27-3.12 (m, 72H). ^{13}C NMR (125 MHz, DMSO-*d₆*) δ: 165.5, 155.9, 155.5, 151.8, 141.7, 140.0, 132.2, 129.3, 106.4, 71.9, 69.8, 69.7, 69.5, 69.0, 68.8, 68.4, 66.8, 59.8, 56.1, 52.9, 48.1, 43.2. IR (KBr): 3330, 2972, 1705, 1538, 1250 cm^{-1}. MALDI-TOF MS (sinapic acid, linear mode, *m/z*): 9664.12. Calcd. for $[M+H]^+$, $C_{386}H_{612}N_{98}O_{190}$: 9665.15.

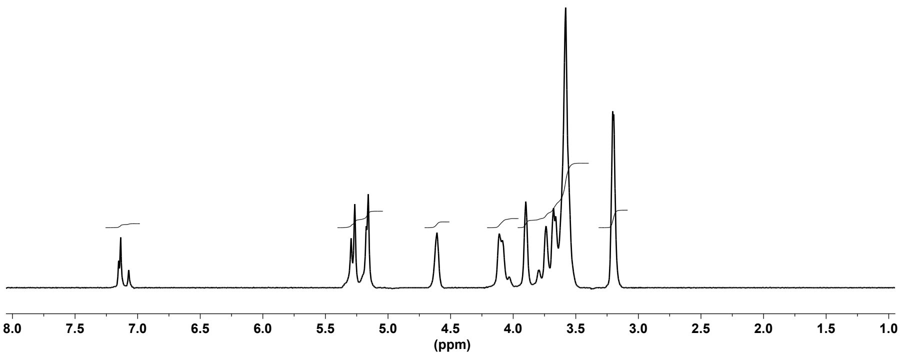

^{1}H NMR spectrum of 2[G3]-OH

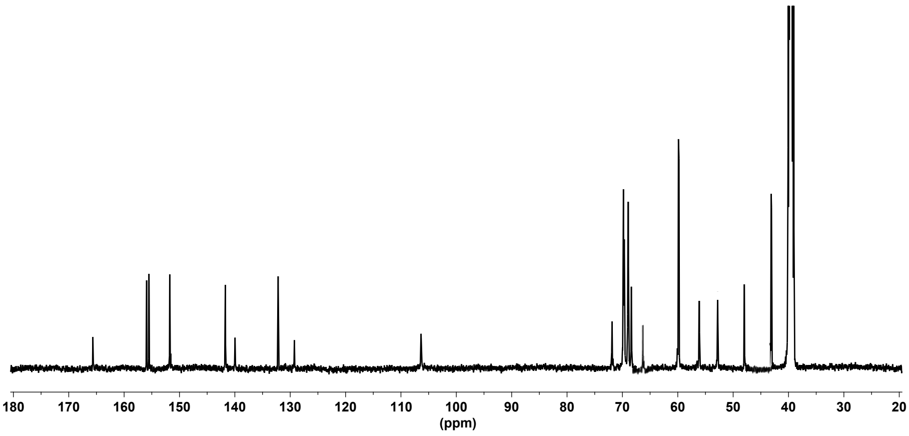

^{13}C NMR spectrum of 2[G3]-OH

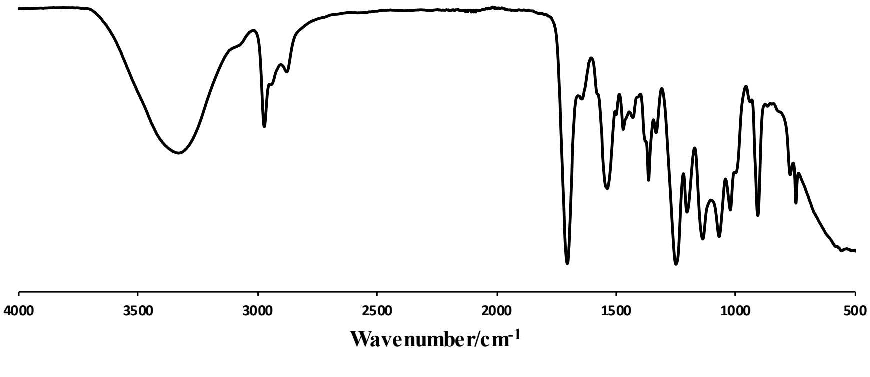

IR spectrum of 2[G3]-OH

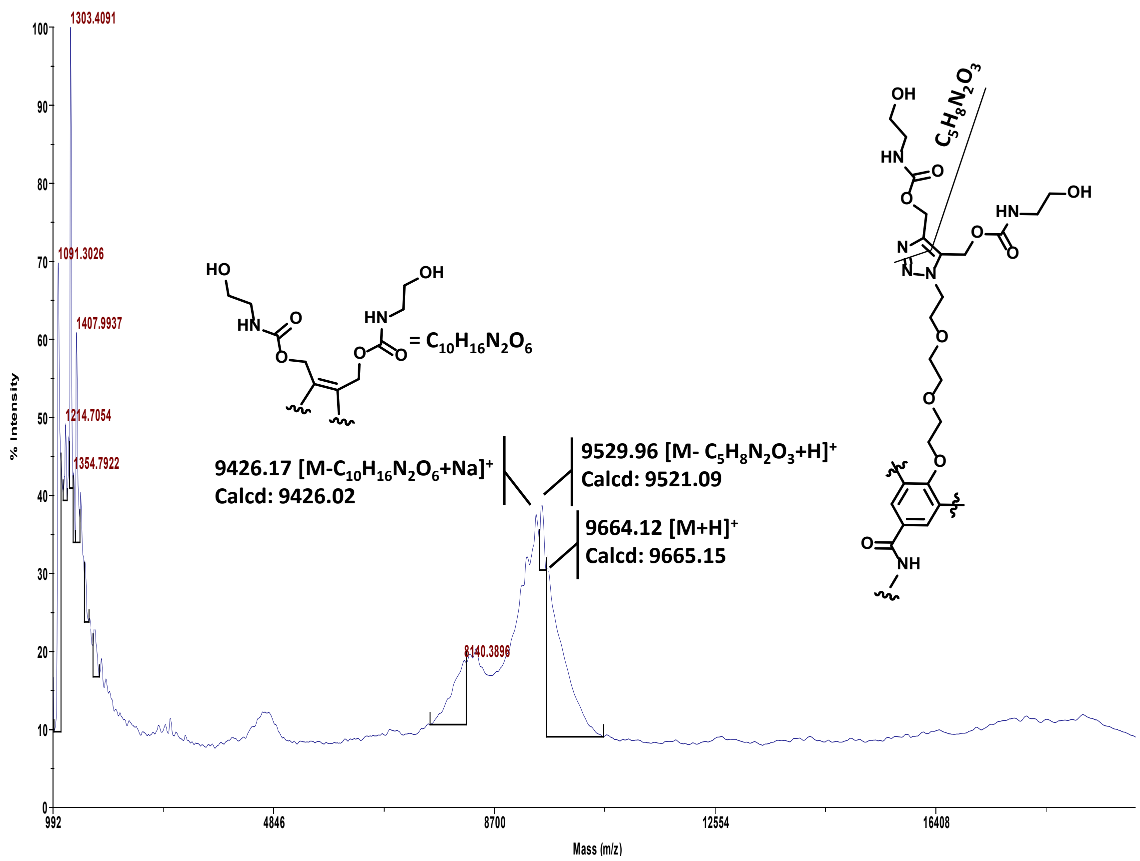

MALDI-TOF MS of 2[G3]-OH

2[G3]-TEG-NH$_2$·HCl. In a Schlenk flask, Alk-TEG-NH$_2$·HCl (129 mg, 253 μmol) was added to a solution of 2[G2]-N$_3$ (35 mg, 7 μmol) in *t*-BuOH/H$_2$O 5:1 (127 μL). The reaction was stirred at 120 °C for 8 h and then was purified by ultrafiltration (4 x 30 mL H$_2$O, Amicon YM1) and lyophilized to afford 2[G3]-TEG-NH$_2$·HCl as a white foam (92 mg, 93%). ^{1}H NMR (500 MHz, diffusion filter 50 ms, D$_2$O) δ: 7.16 (br s, 16H), 5.39-5.11 (m, 72H), 4.73-4.56 (m, 36H), 4.27-4.05 (m, 48H), 3.98-3.45 (m, 504H), 3.34-3.12 (m, 144H). ^{13}C NMR (125 MHz, D$_2$O) δ: 168.9, 157.7, 157.1, 151.9, 142.0, 139.8, 132.7, 129.3, 106.3, 72.1, 69.8, 69.7, 69.4, 69.1, 68.8, 68.4, 66.4, 57.0, 53.8, 48.5, 40.0, 39.0. IR (KBr): 3261, 2935, 1716, 1247, 1113 cm^{-1}.

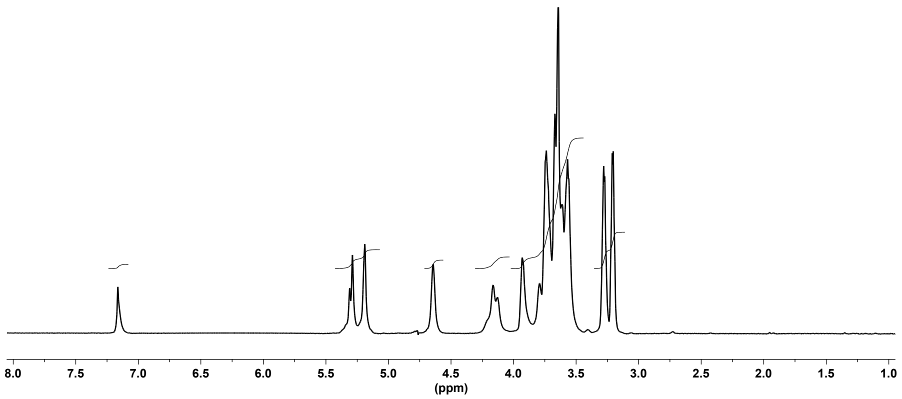

^{1}H NMR spectrum of 2[G3]-TEG-NH_2·HCl

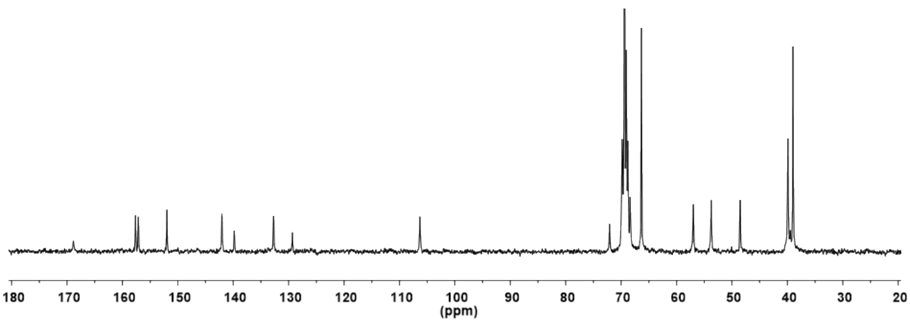

^{13}C NMR spectrum of 2[G3]-TEG-NH_2·HCl

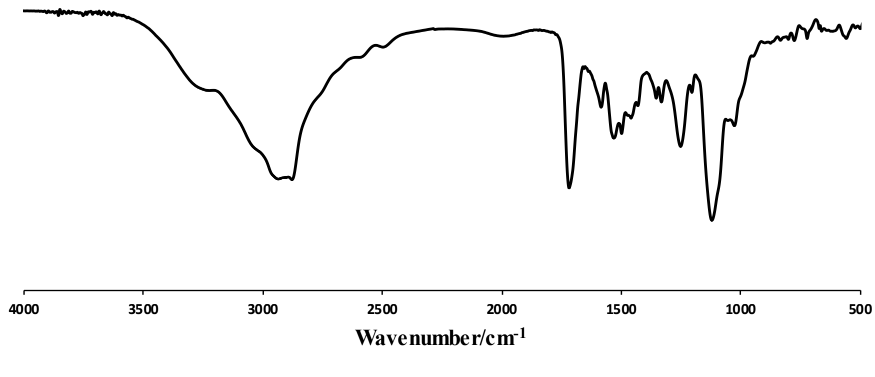

IR spectrum of 2[G3]-TEG-NH_2·HCl

3[G4]-OH. In a Schlenk flask, Alk-OH (90 mg, 346 μmol) was added to a solution of 3[G3]-N_3 (50 mg, 2.10 μmol) in *t*-BuOH/H_2O 5:1 (170 μL). The reaction was stirred at 120 °C for 8 h and then was purified by ultrafiltration (4 x 30 mL acetone/H_2O 1:1 and 2 x 30 mL H_2O, Amicon YM3) and lyophilized to afford 3[G4]-OH as a white foam (86 mg, 91%). ^{1}H NMR (500 MHz, D_2O) δ: 7.19-7.03 (m, 78H), 5.36-5.02 (m, 324H), 4.68-4.47 (m, 162H), 4.22-3.97 (m, 240H), 3.96-3.38 (m, 1602H), 3.26-3.04 (m, 324H). ^{13}C NMR (125 MHz, DMSO-*d6*) δ: 165.6, 155.9, 155.5, 151.8, 141.7, 140.0, 132.2, 129.3, 106.4, 71.8, 69.9, 69.8, 69.6, 69.0, 68.9, 68.4, 59.9, 56.1, 52.8, 48.0, 43.1. IR (KBr): 3351, 2931, 1643, 1251, 1114 cm^{-1}.

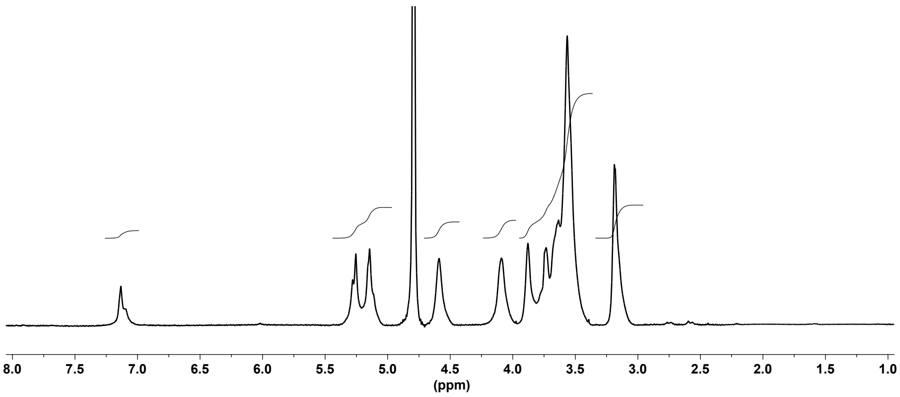

^{1}H NMR spectrum of 3[G4]-OH

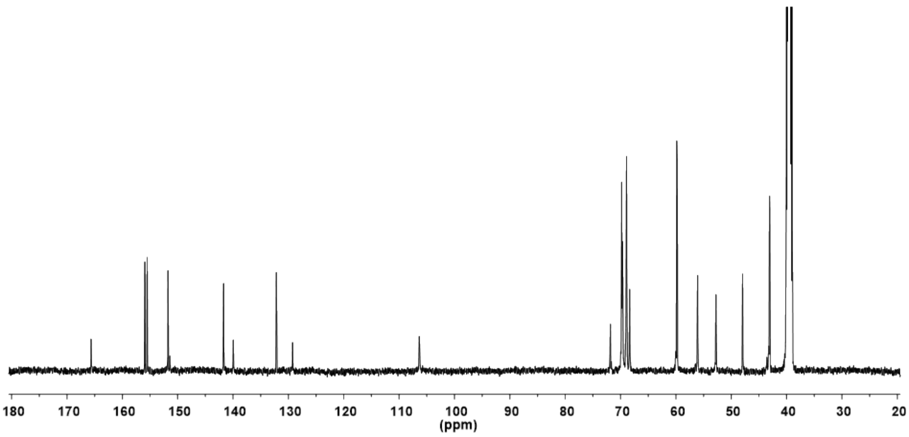

^{13}C NMR spectrum of 3[G4]-OH

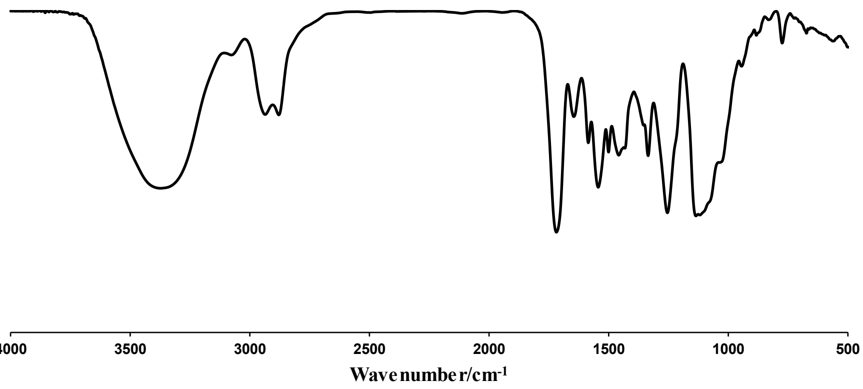

IR spectrum of 3[G4]-OH

3[G4]-TEG-OH. In a Schlenk flask, Alk-TEG-OH (148 mg, 309 μmol) was added to a solution of 3[G3]-N_3 (50 mg, 2.10 μmol) in *t*-BuOH/H_2O 2:1 (170 μL). The reaction was stirred at 120 °C for 8 h and then was purified by ultrafiltration (4 x 30 mL H_2O, Amicon YM3) and lyophilized to afford 3[G4]-TEG-OH as a pale yellow oil (114 mg, 92%). ^1H NMR (500 MHz, D_2O) δ: 7.24-7.10 (m, 78H), 5.40-5.13 (m, 324H), 4.74-4.58 (m, 162H), 4.26-4.01 (m, 240H), 3.99-3.40 (m, 2658H), 3.34-3.18 (m, 324H). ^{13}C NMR (125 MHz, D_2O) δ: 168.4, 157.6, 157.2, 152.1, 141.9, 139.8, 132.7, 129.2, 106.1, 72.1, 71.6, 69.8, 69.4, 69.2, 69.0, 68.8, 68.5, 68.3, 60.2, 56.8, 53.6, 48.5, 39.9. IR (KBr): 3332, 2876, 1708, 1246, 1100 cm^{-1}.

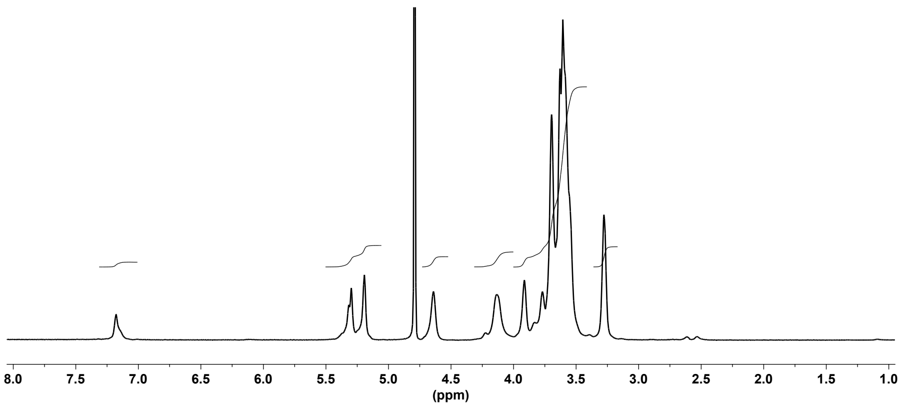

^{1}H NMR spectrum of 3[G4]-TEG-OH

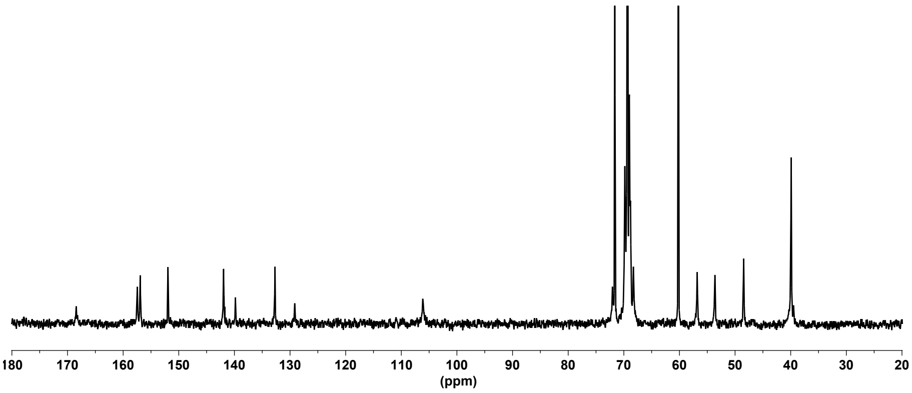

^{13}C NMR spectrum of 3[G4]-TEG-OH

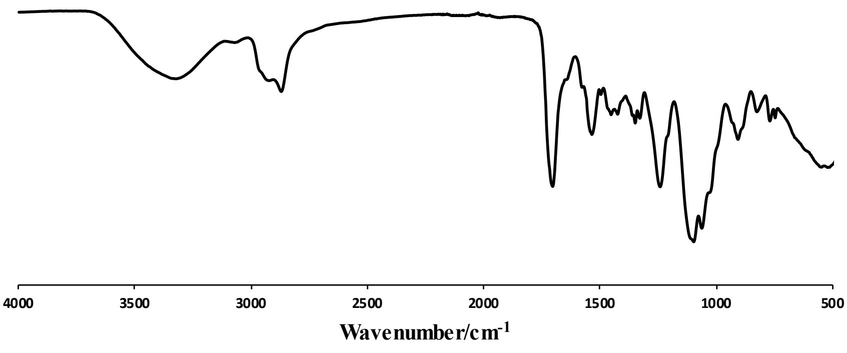

IR spectrum of 3[G4]-TEG-OH

3[G4]-PhOH. In a Schlenk flask, Alk-PhOH (130 mg, 339 μmol) was added to a solution of 3[G3]-N_3 (50 mg, 2.10 μmol) in MeOH/H_2O 5:1 (170 μL). The reaction was stirred at 120 ºC for 8 h and then was purified by ultrafiltration (4 x 30 mL acetone/H_2O 2:1 and 2 x 30 mL H_2O, Amicon YM3) and lyophilized to afford 3[G4]-PhOH as a white solid (105 mg, 91%). ^{1}H NMR (500 MHz, DMSO-d_6) δ: 9.25 (br s, 162H), 8.44 (br s, 36H), 7.67 (s, 81H), 7.55 (s, 81H), 7.17 (s, 78H), 6.99 (d, J = 7.4 Hz, 324H), 6.66 (d, J = 7.4 Hz, 324H), 5.28-5.16 (m, 162H), 5.14-5.00 (m, 162H), 4.59-4.44 (m, 162H), 4.19-3.91 (m, 564H), 3.82-3.41 (m, 1038H). ^{13}C NMR (125 MHz, DMSO-d_6) δ: 166.1, 156.7, 156.4, 156.0, 152.2, 142.1, 140.4, 132.6, 130.2, 130.0, 129.6, 128.8, 115.4, 106.7, 72.3, 70.2, 70.1, 69.3, 68.7, 56.6, 53.4, 48.4, 43.9. IR (KBr): 3336, 2923, 1708, 1517, 1241, 1122 cm^{-1}.

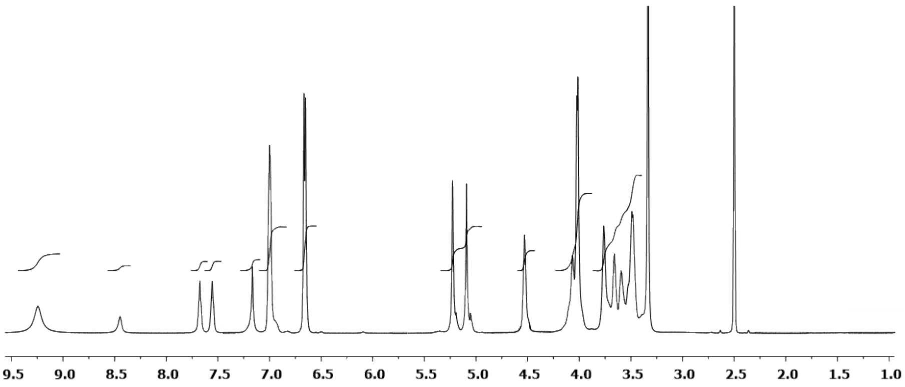

^{1}H NMR spectrum of 3[G4]-PhOH

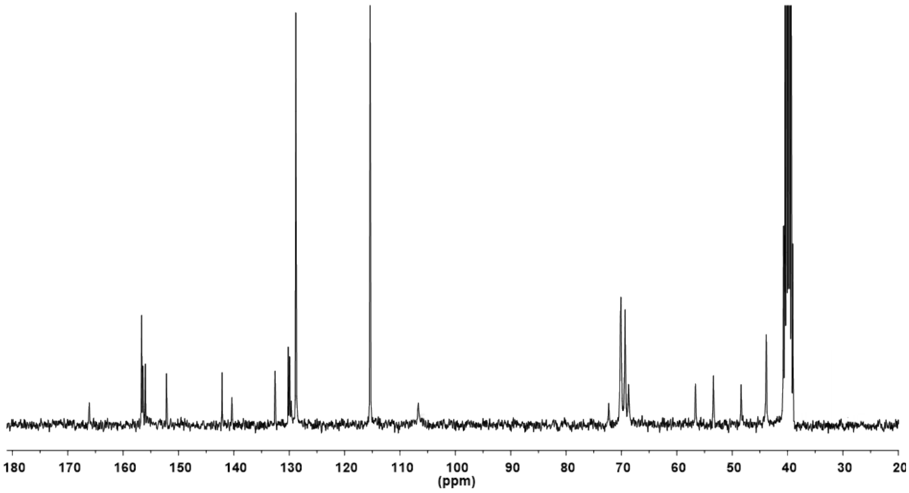

^{13}C NMR spectrum of 3[G4]-PhOH

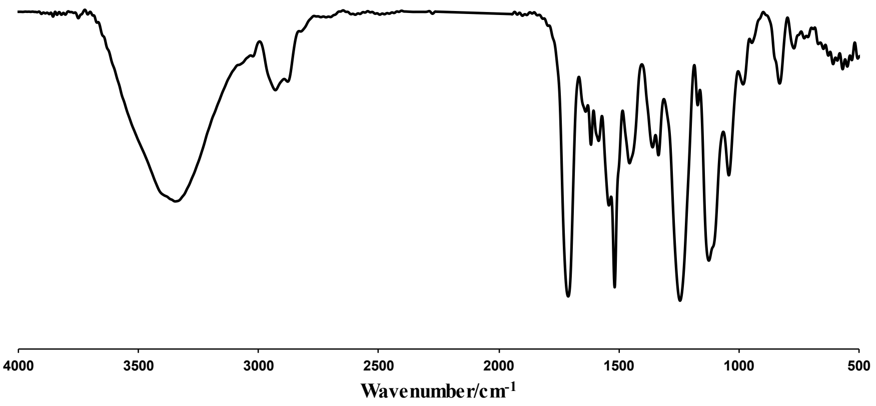

IR spectrum of 3[G4]-PhOH

3[G4]-OSO_3Na. In a Schlenk flask, Alk-$OSO_3H·NH_3$ (145 mg, 319 µmol) was added to a solution of 3[G3]-N_3 (47 mg, 1.97 µmol) in *t*-BuOH/H_2O 1:1 (160 µL). The reaction was stirred at 120 ºC for 8 h and then was purified by ultrafiltration (4 x 30 mL 0.1 M NaOH/acetone 1:1 and 2 x 30 mL H_2O, Amicon YM3) and lyophilized to afford 3[G4]-OSO_3Na as a white foam (108 mg, 91%). ^1H NMR (500 MHz, D_2O) δ: 7.12 (br s, 78H), 6.26 (br s, 3H), 5.43-5.16 (m, 324H), 4.71-4.58 (m, 162H), 4.29-4.01 (m, 564H), 4.00-3.51 (m, 1038H), 3.48-3.32 (m, 324H). ^{13}C NMR (125 MHz, D_2O) δ: 168.8, 157.5, 157.0, 151.8, 141.9, 139.6, 132.6, 129.3, 106.1, 72.1, 69.9, 69.5, 69.0, 68.8, 68.3, 67.2, 67.1, 57.1, 53.9, 48.6, 40.0. IR (KBr): 3446, 2935, 1718, 1542, 1255 cm^{-1}.

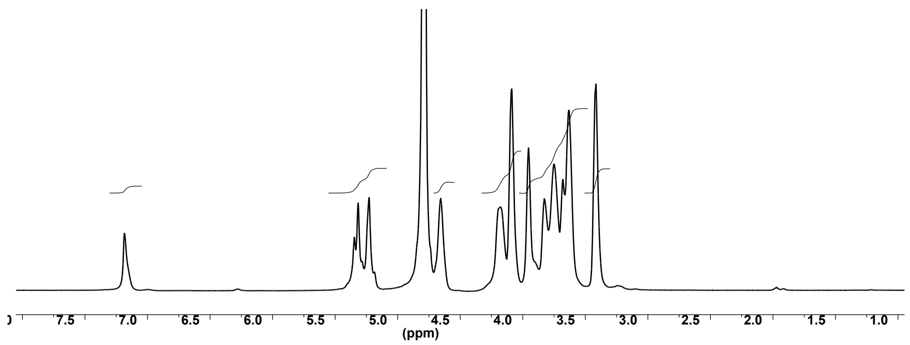

^{1}H NMR spectrum of 3[G4]-OSO_3Na

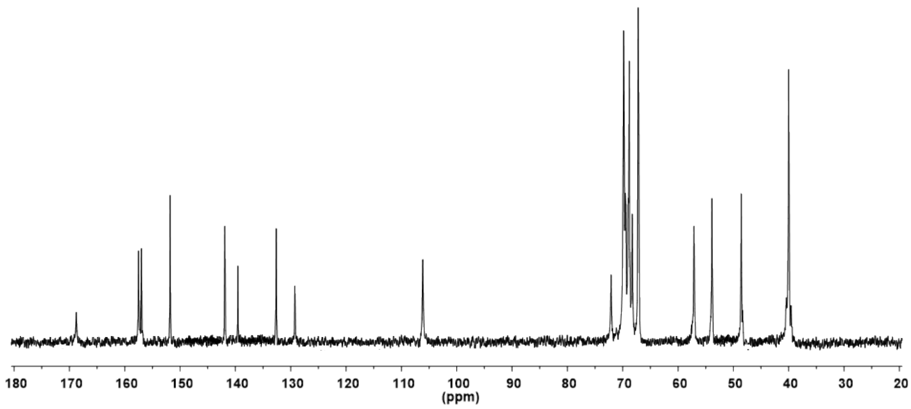

^{13}C NMR spectrum of 3[G4]-OSO_3Na

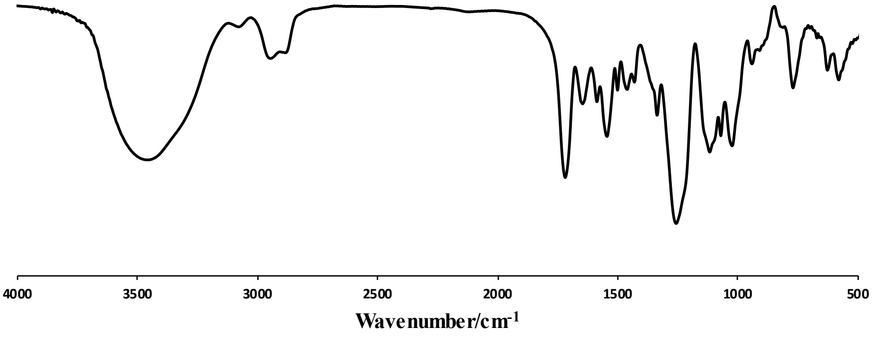

IR spectrum of 3[G4]-OSO_3Na

3[G4]-Man. In a Schlenk flask, Alk-Man (120 mg, 158 µmol) was added to a solution of 3[G3]-N_3 (24 mg, 0.974 µmol) in *t*-BuOH/H_2O 5:1 (160 µL). The reaction was stirred at 120 ºC for 12 h and then was purified by ultrafiltration (4 x 30 mL H_2O, Amicon YM3) and lyophilized to afford 3[G4]-Man as a beige foam (78 mg, 93%). ^{1}H NMR (500 MHz, D_2O) δ: 7.18 (br s, 78H), 6.23 (br s, 3H), 5.43-5.15 (m, 324H), 4.90 (s, 162H), 4.76-4.60 (m, 162H), 4.24-4.07 (m, 240H), 4.05-3.48 (m, 3306H), 3.37-3.21 (m, 324H). ^{13}C NMR (125 MHz, D_2O) δ: 168.9, 157.9, 157.4, 152.4, 142.4, 140.3, 133.2, 129.6, 106.7, 100.4, 73.2, 72.6, 71.0, 70.5, 70.3, 70.1, 70.0, 69.9, 69.7, 69.6, 69.4, 68.9, 67.2, 66.7, 61.4, 60.8, 57.4, 54.3, 49.1, 40.6. IR (KBr): 3315, 1638, 1248, 1061 cm^{-1}.

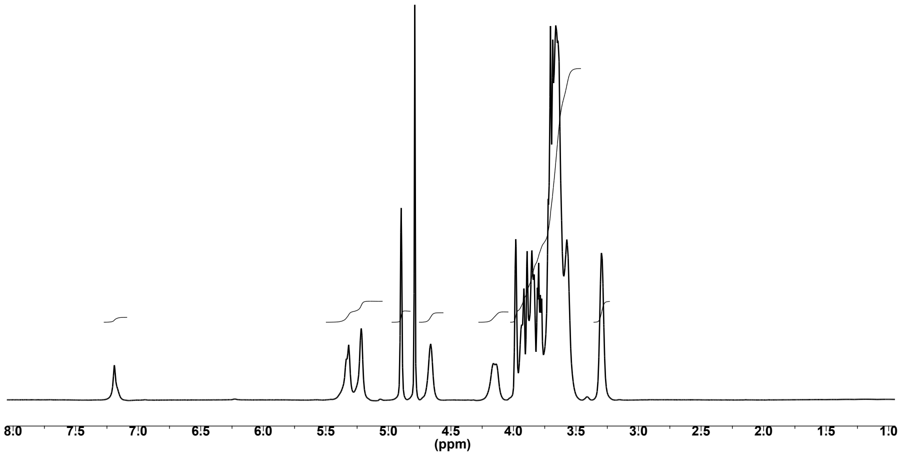

^{1}H NMR spectrum of 3[G4]-Man

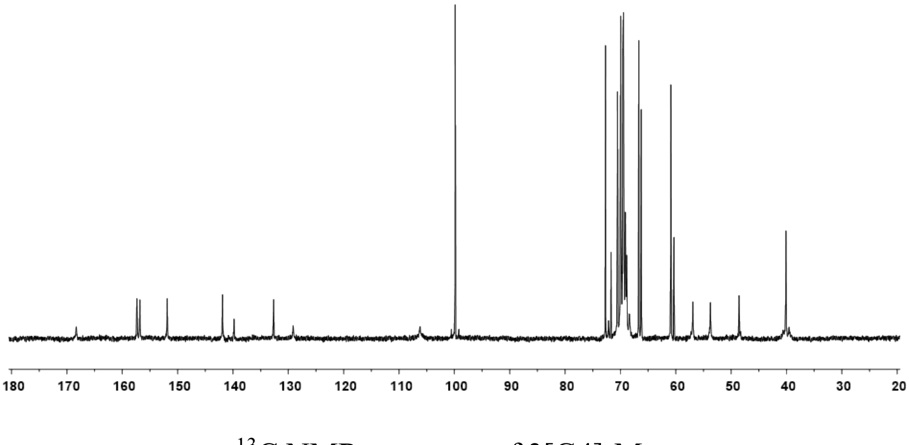

^{13}C NMR spectrum of 3[G4]-Man

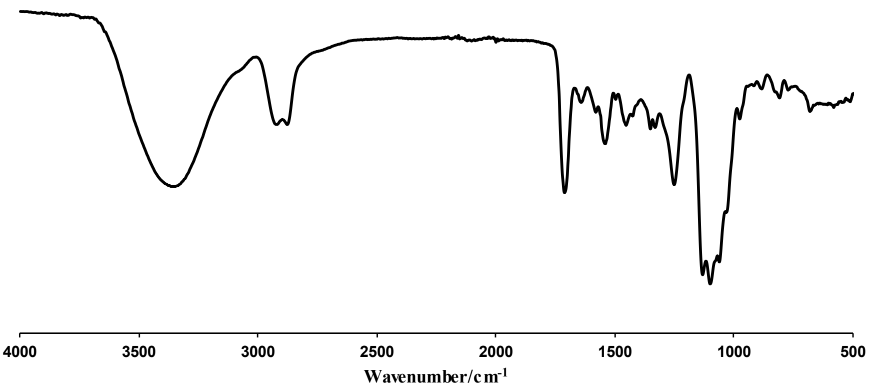

IR spectrum of 3[G4]-Man

3[G4]-Glc. In a Schlenk flask, Alk-Glc (135 mg, 177 μmol) was added to a solution of 3[G3]-N_3 (26 mg, 1.1 μmol) in *t*-BuOH/H_2O 1:1 (178 μL). The reaction was stirred at 120 °C for 14 h and then was purified by ultrafiltration (4 x 30 mL H_2O, Amicon YM3) and lyophilized to afford 3[G4]-Glc as a beige foam (85 mg, 92%). ^1H NMR (500 MHz, D_2O) δ: 7.18 (br s, 78H), 6.23 (br s, 3 H), 5.40-5.13 (m, 324H), 4.72-4.58 (m, 162H), 4.52-4.43 (m, 162H), 4.26-3.99 (m, 402H), 3.98-3.36 (m, 3144H), 3.35-3.21 (m, 324H). ^{13}C NMR (125 MHz, D_2O) δ: 168.9, 157.9, 157.4, 152.4, 142.4, 140.3, 133.2, 129.7, 106.7, 102.7, 76.4, 76.2, 73.6, 70.4, 70.1, 69.9, 69.8, 69.6, 69.4, 69.0, 61.3, 57.5, 54.3, 49.1, 40.6. IR (KBr): 3354, 2878, 1710, 1251, 1079 cm^{-1}.

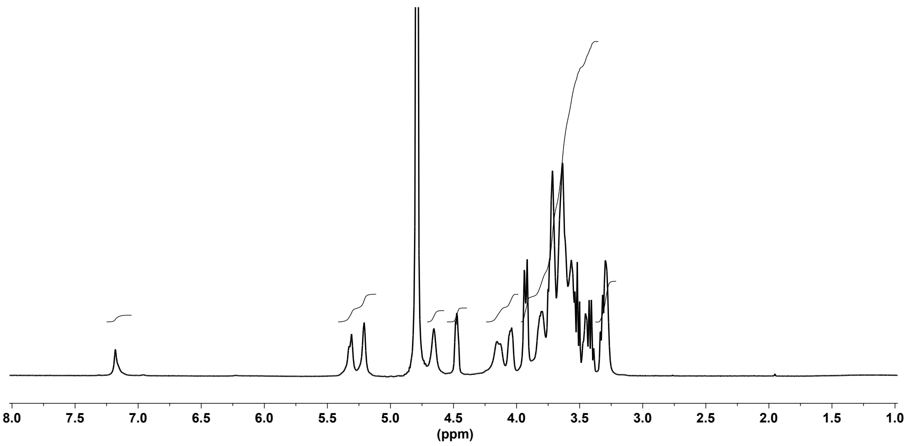

^{1}H NMR spectrum of 3[G4]-Glc

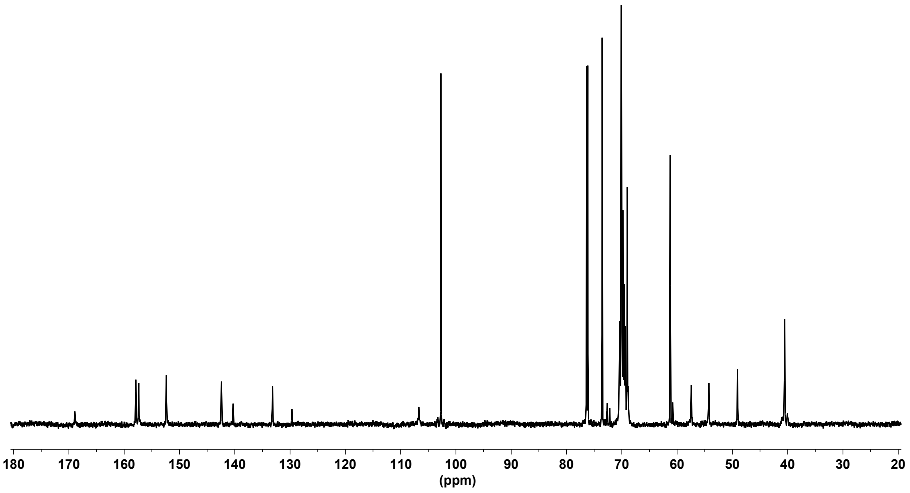

^{13}C NMR spectrum of 3[G4]-Glc

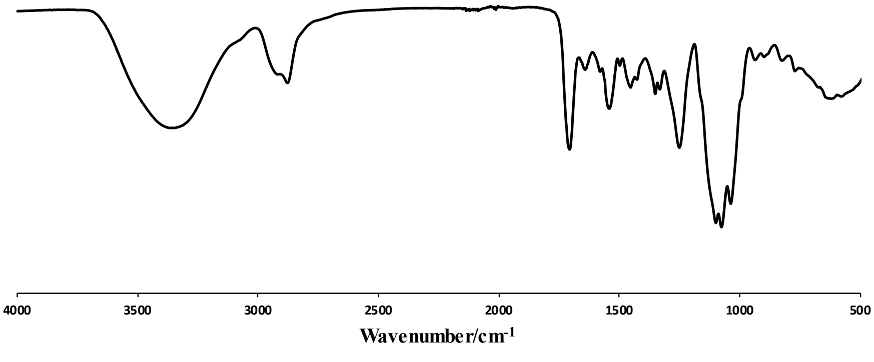

IR spectrum of 3[G4]-Glc

3[G4]-TEG-NH$_2$·HCl. In a Schlenk flask, Alk-TEG-NH$_2$·HCl (173 mg, 339 μmol) was added to a solution of 3[G3]-N$_3$ (50 mg, 2.10 μmol) in *t*-BuOH/H$_2$O 1:1 (170 μL). The reaction was stirred at 120 °C for 8 h and then was purified by ultrafiltration (4 x 30 mL H$_2$O, Amicon YM3) and lyophilized to afford 3[G4]-TEG-NH$_2$·HCl as a white foam (125 mg, 92%). ^{1}H NMR (500 MHz, D$_2$O) δ: 7.16 (br s, 78H), 5.40-5.12 (m, 324H), 4.71-4.55 (m, 162H), 4.29-4.02 (m, 240H), 4.00-3.46 (m, 2334H), 3.34-3.17 (m, 648H). ^{13}C NMR (125 MHz, D$_2$O) δ: 168.7, 157.6, 157.0, 151.9, 141.9, 139.7, 132.7, 129.2, 106.2, 72.0, 69.8, 69.7, 69.4, 69.0, 68.7, 68.3, 66.2, 56.9, 53.7, 48.5, 39.8, 38.9. IR (KBr): 3383, 2971, 1677, 1201, 1130 cm^{-1}.

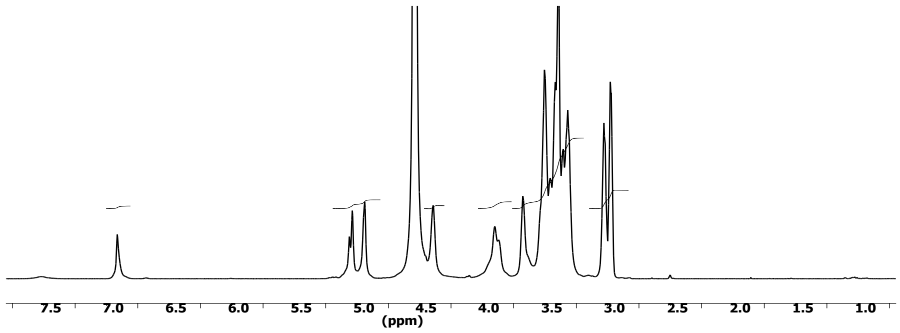

^{1}H NMR spectrum of 3[G4]-TEG-NH$_2$·HCl

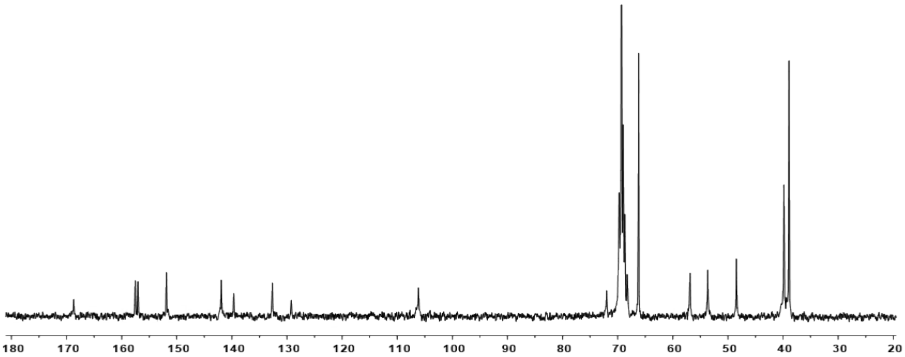

^{13}C NMR spectrum of 3[G4]-TEG-NH$_2$·HCl

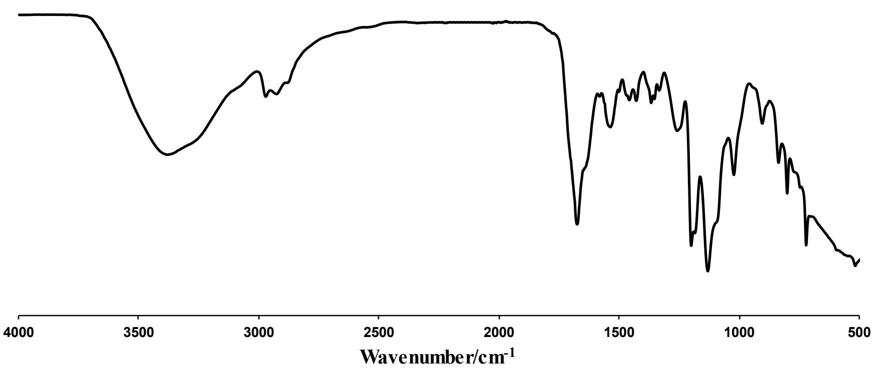

IR spectrum of 3[G4]-TEG-NH_2·HCl

3[G4]-DOTA. In a Schlenk flask, p-NCS-Bz-DOTA-GA (96 mg, 173 μmol) was added to a solution of Alk-TEG-NH_2·HCl (25 mg, 57.6 μmol) and Et_3N (48 μL, 346 μmol) in dry DMSO (30 μL) under Ar. After 24 h of stirring at rt, 3[G3]-N_3 (4.4 mg, 0.18 μmol) was added. The reaction mixture was stirred at 120 °C for 32 h and then was purified by ultrafiltration (4 x 30 mL 50 mM PB pH 7.4 and 3 x 30 mL H_2O, Amicon YM3) and lyophilized to afford 3[G4]-DOTA as a beige foam (26.0 mg, 90%). ^{1}H NMR (500 MHz, diffusion filter 100 ms, D_2O) δ: 7.46-7.07 (m, 726H), 5.40-5.08 (m, 324H), 4.68-4.55 (m, 162H), 4.50-4.25 (m, 324H), 4.03-2.67 (m, ~7356H), 2.56-2.32 (m, 324H), 2.10-1.80 (m, 324H). IR (KBr): 3319, 2872, 1712, 1631, 1540, 1094 cm^{-1}.

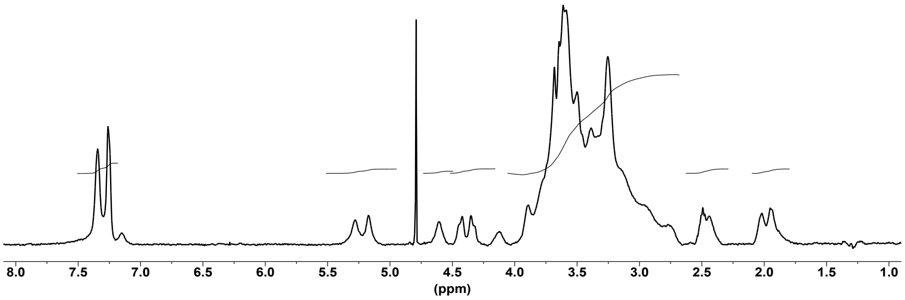

^{1}H NMR spectrum of 3[G4]-DOTA

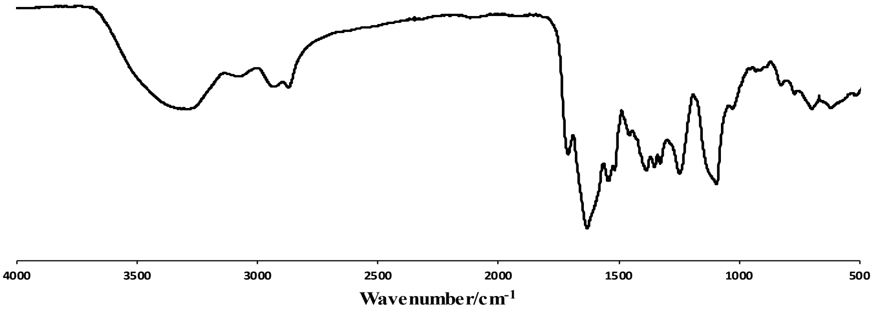

IR spectrum of 3[G4]-DOTA

3[G4]-Man$_{120}$/Bio$_{32}$/FITC$_{10}$. In a Schlenk flask, Alk-Man (38 mg, 50 μmol), Alk-Bio (12 mg, 13.4 μmol), and Alk-FITC (5.1 mg, 4.2 μmol) were added to a solution of 3[G3]-N_3 (10 mg, 0.42 μmol) in *t*-BuOH/H_2O 1:1 (68 μL). The reaction was stirred at 120 ºC for 14 h and then was purified by ultrafiltration (5 x 30 mL H_2O, Amicon YM3) and lyophilized to afford 3[G4]-Man$_{120}$/Bio$_{32}$/FITC$_{10}$ as a pale red foam (36 mg, 96%). ^{1}H NMR (500 MHz, D_2O) δ: 7.38-7.05 (m, 104H), 6.71-6.63 (m, 10H), 5.45-5.09 (m, 324H), 4.90 (s, 120H), 4.74-4.55 (m, 162H), 4.54-4.45 (m, 32H), 4.34-4.03 (m, 272H), 4.02-3.42 (m, 4798H), 3.41-3.09 (m, 340H), 2.93-2.64 (m, 64H), 2.30-2.12 (m, 64H), 1.71-1.17 (m, 192H). ^{13}C NMR (125 MHz, D_2O) δ: 176.2, 168.5, 165.1, 157.5, 156.9, 151.9, 141.9, 140.9, 132.7, 129.2, 106.1, 100.1, 72.6, 72.1, 71.6, 70.4, 69.9, 69.5, 69.4, 69.1, 68.8, 68.3, 66.6, 66.2, 62.4, 61.4, 60.8, 60.7, 57.5, 55.8, 54.3, 49.1, 48.8, 40.6, 40.2, 39.9, 39.3, 35.9, 28.5, 28.2, 25.7. IR (KBr): 3326, 2928, 1581, 1025 cm^{-1}.

An average incorporation of 10 FITC per dendrimer was determined by absorbance at 494 nm (using an extinction coefficient of 73000 M^{-1} cm^{-1}, DMSO), while degrees of substitution of 120 for mannose and 32 for biotin were determined by integration of characteristic signals in the ^{1}H NMR spectrum (singlet at 4.90 ppm for mannose, multiplets at 2.93-2.64 and 2.30-2.12 ppm for biotin).

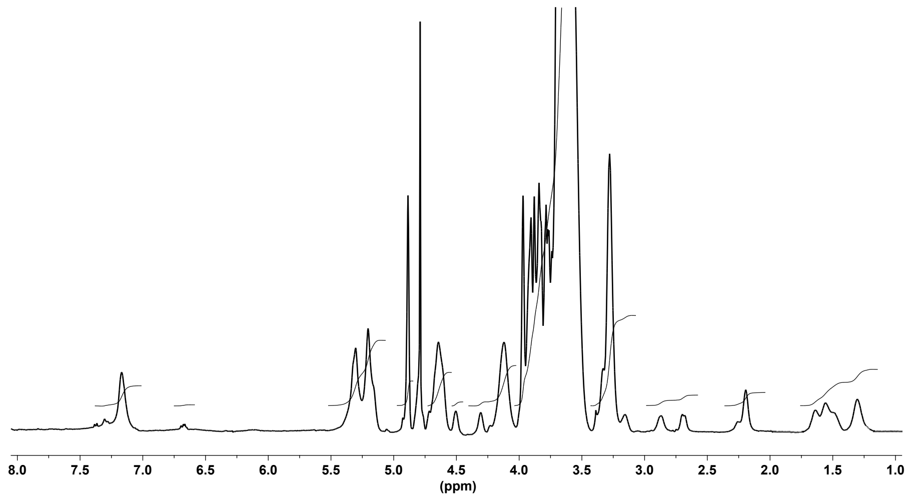

^{1}H NMR spectrum of 3[G4]-Man_{120}/Bio_{32}/$FITC_{10}$

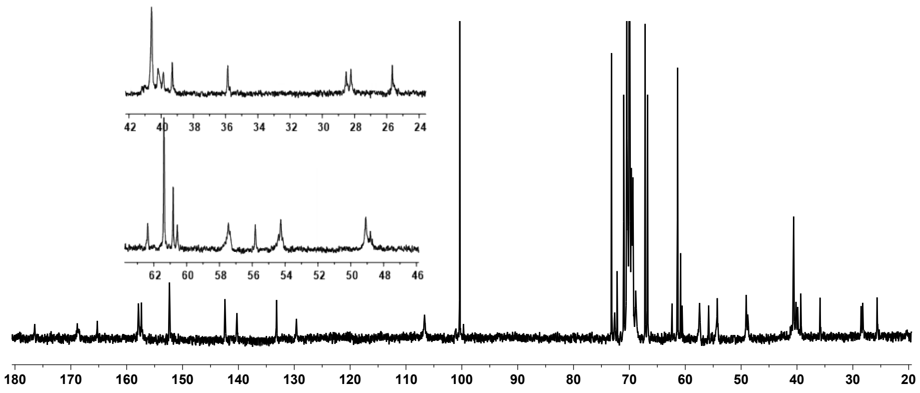

^{13}C NMR spectrum of 3[G4]-Man_{120}/Bio_{32}/$FITC_{10}$

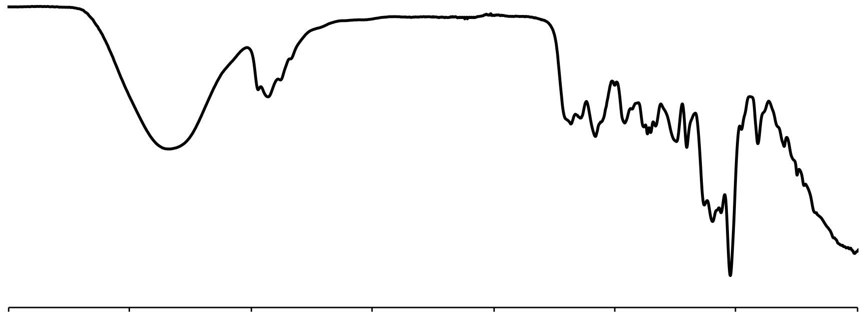

IR spectrum of 3[G4]-Man_{120}/Bio_{32}/$FITC_{10}$

3[G5]-Man. In a Schlenk flask, Alk-Man (123 mg, 162 μmol) was added to a solution of 3[G4]-N_3 (25 mg, 0.346 μmol) in *t*-BuOH/H_2O 5:1 (168 μL). The reaction was stirred at 120 ºC for 12 h and then was purified by ultrafiltration (4 x 30 mL H_2O, Amicon YM5) and lyophilized to afford 3[G5]-Man as a beige foam (84 mg, 94%). ^1H NMR (500 MHz, D_2O) δ: 7.17 (br s, 240H), 5.40-5.13 (m, 972H), 4.89 (s, 486H), 4.75-4.57 (m, 486H), 4.27-4.05 (m, 762H), 4.02-3.45 (m, 10920H), 3.35-3.20 (m, 972H). ^{13}C NMR (125 MHz, D_2O) δ: 168.3, 157.4, 156.8, 151.9, 141.9, 139.8, 132.6, 129.1, 106.2, 99.9, 72.7, 72.2, 71.7, 70.5, 70.0, 69.9, 69.7, 69.6, 69.4, 69.1, 68.9, 68.4, 66.7, 66.2, 60.9, 60.3, 56.9, 53.7, 48.6, 40.1. IR (KBr): 3409, 2927, 1712, 1251, 1095 cm^{-1}.

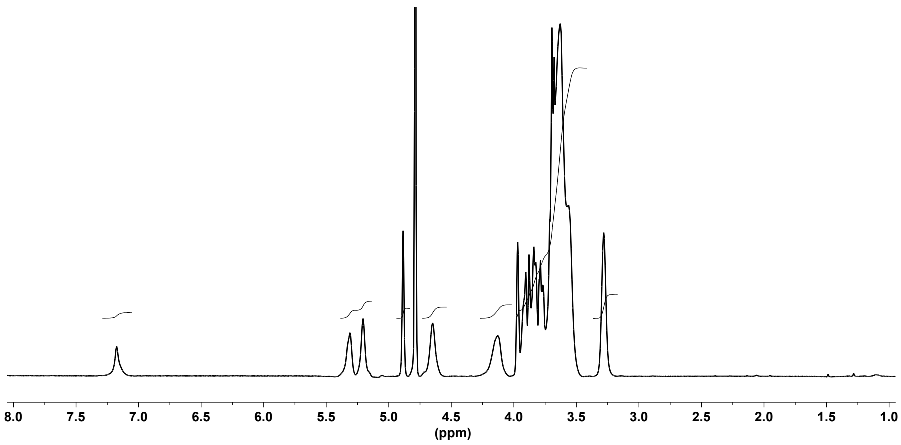

^1H NMR spectrum of 3[G5]-Man

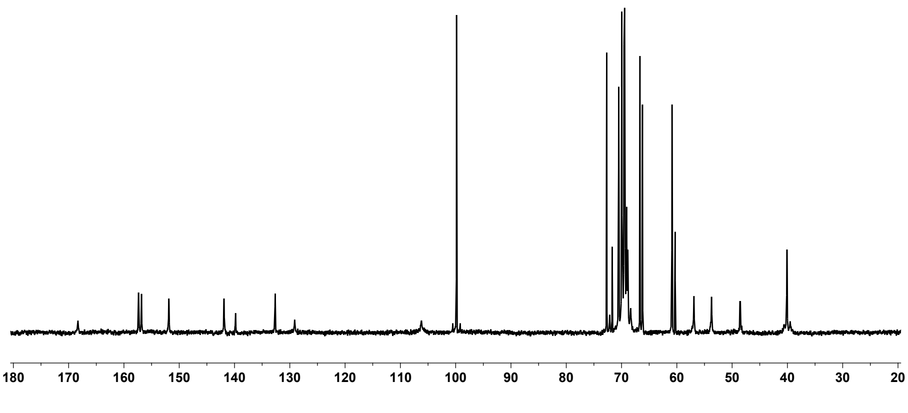

^{13}C NMR spectrum of 3[G5]-Man

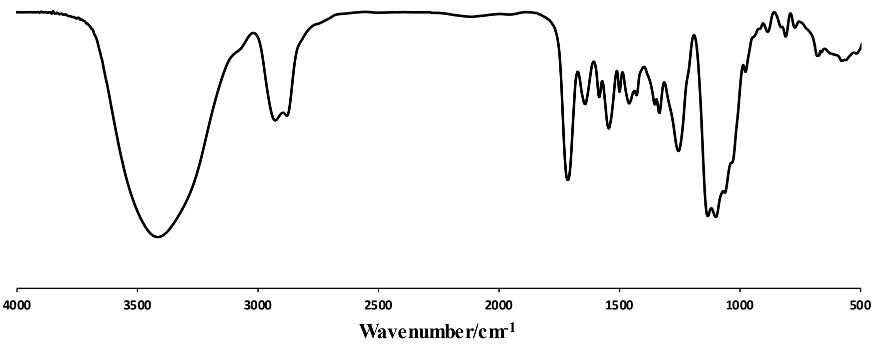

IR spectrum of 3[G5]-Man

PEG-[G3]-Cat. In a Schlenk flask, Alk-Cat (10.0 mg, 23.5 μmol) was added to a solution of PEG-[G2]-N_3 (10.0 mg, 1.31 μmol) in *t*-BuOH/H_2O 1:1 (24 μL). The reaction was stirred at 120 ºC for 12 h and then was purified by ultrafiltration (4 x 30 mL acetone/H_2O 3:1 and H_2O 2 x 30 mL, Amicon YM3) and lyophilized to afford PEG-[G3]-Cat as a beige foam (13.9 mg, 92%). ^{1}H NMR (500 MHz, DMSO-*d*$_6$) δ: 8.78-8.39 (m, 36H), 7.38-7.25 (m, 18H), 7.24-7.12 (m, 24H), 6.66-6.31 (m, 54H), 5.26-5.01 (m, 36H), 4.59-4.46 (m, 18H), 4.20-3.95 (m, 24H), 3.86-2.98 (m, 634H). IR (KBr): 3318, 2877, 1702, 1361, 1221, 1091 cm^{-1}.

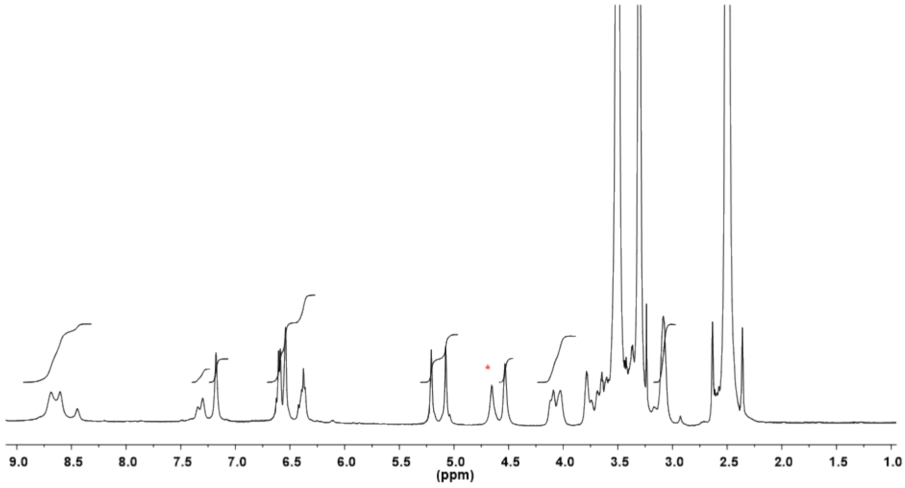

^{1}H NMR spectrum of PEG-[G3]-Cat

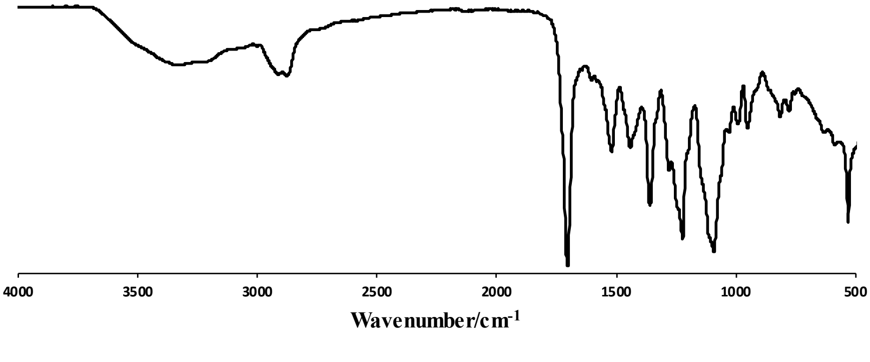

IR spectrum of PEG-[G3]-Cat

PEG-PGA-[G1]-OH. In a Schlenk flask, Alk-OH (12.0 mg, 46 μmol) was added to a solution of PEG-PGA-N_3 (10.0 mg, 0.9 μmol) in *t*-BuOH/H_2O 1:1 (46 μL). The reaction was stirred at 120 ºC for 12 h and then was purified by ultrafiltration (4 x 30 mL H_2O, Amicon YM3) and lyophilized to afford PEG-PGA-[G1]-OH as a pale yellow foam (14.7 mg, 92%). ^1H NMR (500 MHz, D_2O) δ: 5.41-5.10 (m, 92H), 4.56-4.24 (m, 69H), 3.93-3.51 (m, 568H), 3.32-3.05 (m, 138H), 2.46-2.24 (m, 46H), 2.23-1.89 (m, 96H). IR (KBr): 3295, 2878, 1703, 1649, 1535, 1249, 1076 cm^{-1}.

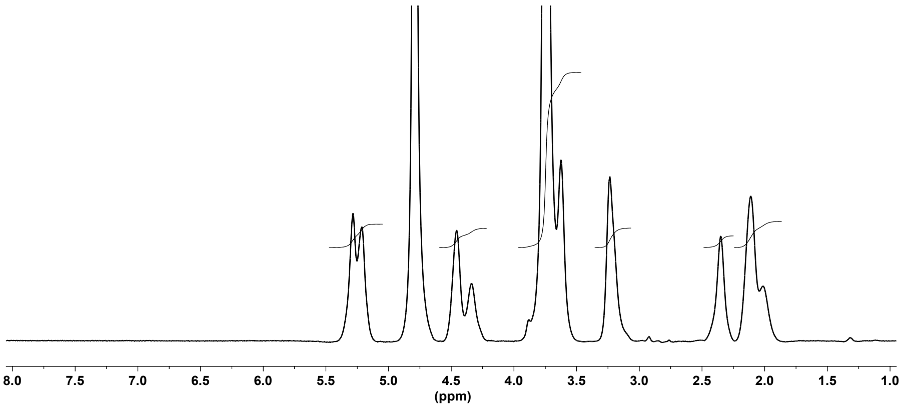

^1H NMR spectrum of PEG-PGA-[G1]-OH

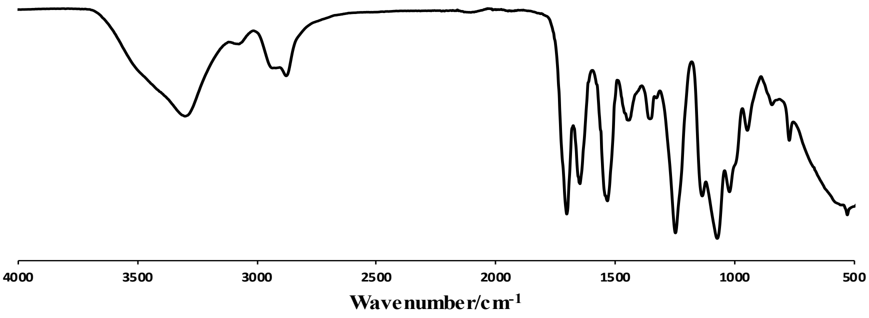

IR spectrum of PEG-PGA-[G1]-OH

3. DLS Size Distributions

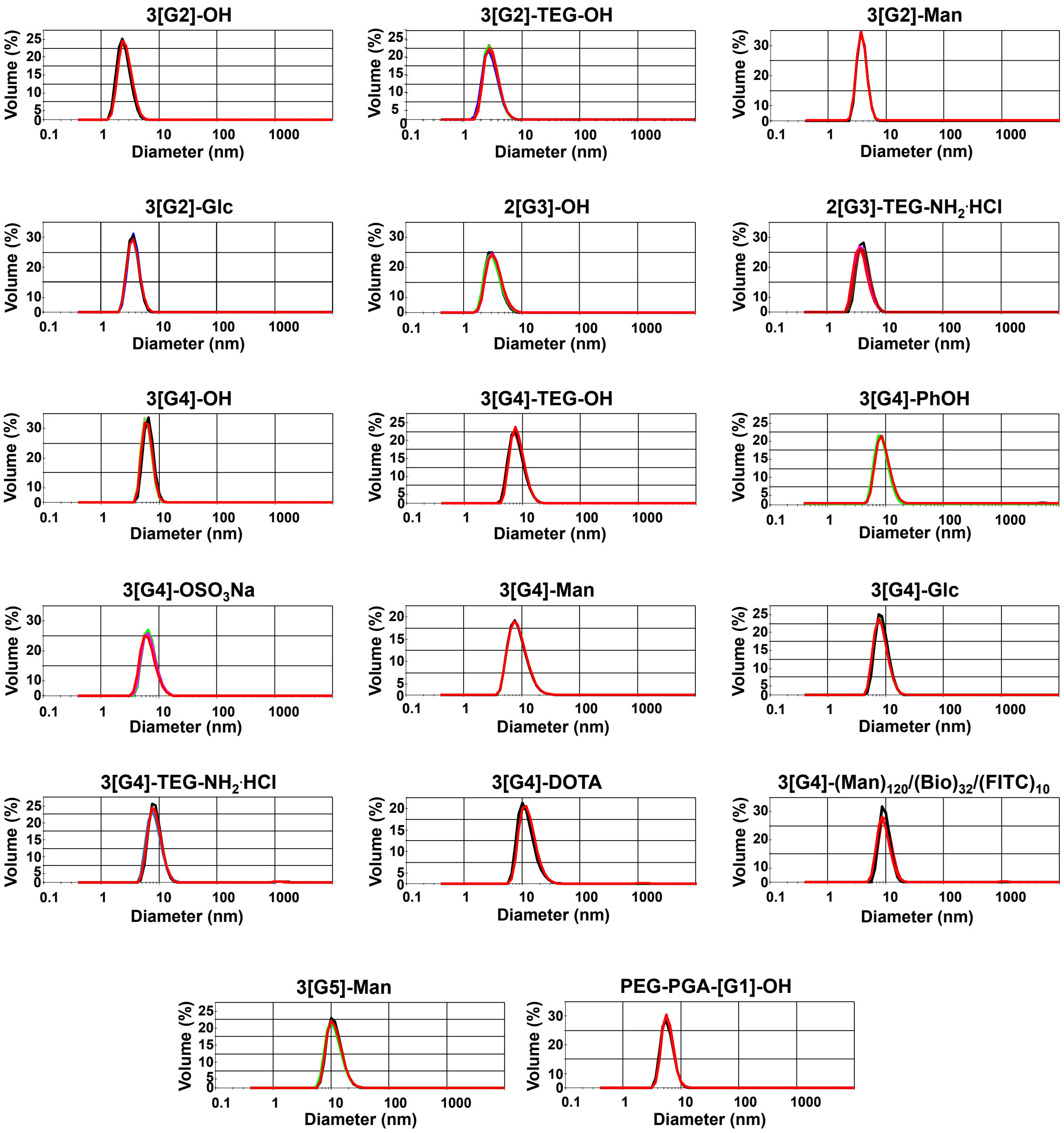

Figure S1. DLS size distributions of multivalently functionalized scaffolds (1 mg/mL, 10 mM PB pH 7.4, 150 mM LiCl, 25 ºC; 3[G4]-PhOH measured at 1 mg/mL, THF, 25 ˚C).

4. GPC Elugrams

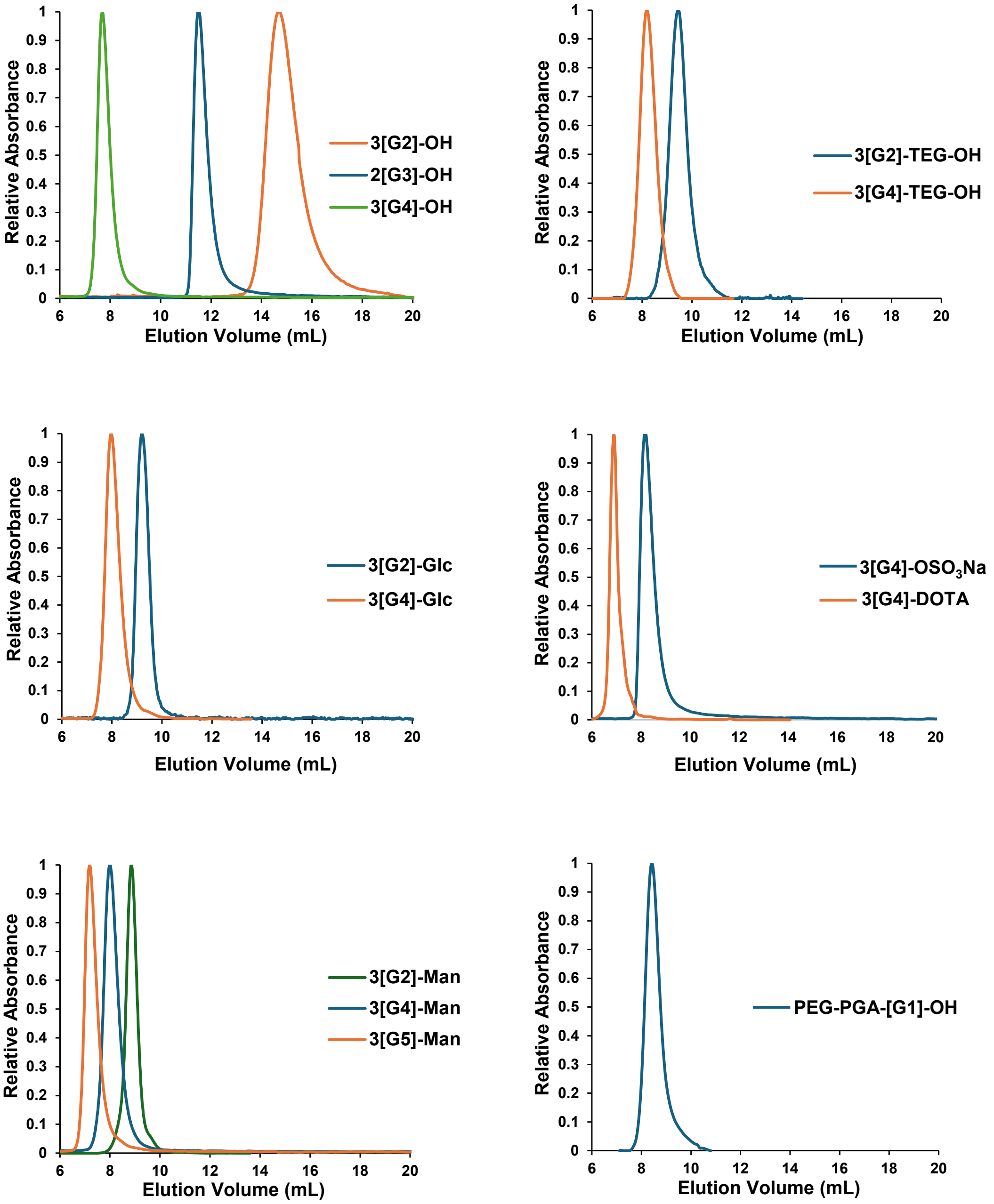

Figure S2. GPC elugrams of multivalently functionalized scaffolds (1 mg/mL, 10 mM PB pH 7.4, 150 mM LiCl, 25 ºC).

5. Preparation of PEG-[G3]-Cat Micelles and Encapsulation of DOX

PEG-[G3]-Cat was dissolved in 10 mM PB pH 7.4, 150 mM NaCl (1 mg/mL) and the solution was immediately filtered through 0.45 μm nylon filters. Formation of micelles was confirmed by DLS after 1 h of stirring at rt. DOX was dissolved in 150 mM NaCl (1 mg/mL) and filtered through 0.45 μm nylon filters.

DOX-loaded micelles were prepared by adding a solution of PEG-[G3]-Cat micelles over a solution of DOX (volume ratio 2:1). The resulting solution was left in the dark under stirring at 37 ºC for 48 h. Unloaded DOX was removed by dialysis (Spectra/Por® 6, MWCO 1 kDa) against 250 mL of 10 mM PB pH 7.4, 150 mM NaCl for 12 h in the dark at 37 ºC. To determine the EE and DL of DOX, an aliquot of the buffer solution was taken at the end of the dialysis (150 μL) and placed in a 96-well microplate (done in triplicate). Then, 500 mM acetate pH 4 buffer (50 μL) was added to each well and the fluorescence of the samples was measured in a microplate reader (exc. 485±20 nm, em. 535±20 nm; Tecan Infinite F200 PRO). The concentration of unloaded DOX in solution was determined by comparison with a standard calibration curve made from the fluorescence emission of fresh solutions of DOX of known concentration prepared under identical conditions. An EE of 87% and DL of 29% were obtained.

6. Determination of the Bioactivity of Biotin on 3[G4]-Man_{120}/Bio_{32}/$FITC_{10}$ by Staining of Agarose Beads Functionalized with Streptavidin (SA)

A suspension of agarose beads functionalized with streptavidin (SA–agarose; 25 μL, 10.1 mg/mL in 10 mM PB pH 7.4, 150 mM NaCl) was added to a solution of 3[G4]-Man_{120}/Bio_{32}/$FITC_{10}$ (1.0 mL, 1.0 mg/mL in 10 mM PB pH 6.5, 150 mM NaCl). The

resulting suspension was orbitally shaken in the dark for 2 h at 37 ºC. Then, the beads were centrifuged (1000 g, 30 s), washed (10 mM PB pH 6.5, 150 mM NaCl), and centrifuged again. After three additional washing/centrifugation cycles, beads were finally suspended in 10 mM PB pH 7.4, 150 mM NaCl and analyzed by fluorescence microscopy on an Olympus BX-51 microscope equipped with an Olympus DP-71 camera. Excitation of SA–agarose beads was carried out in the green channel (blue excitation U-MWB2: excitation filter 460-490 nm, emission filter 520 nm, dichromatic mirror 500 nm). Unfunctionalized agarose beads (no streptavidin) treated with 3[G4]-Man_{120}/Bio_{32}/$FITC_{10}$ under identical conditions were used as control.

7. Determination of the Bioactivity of Mannose on 3[G4]-Man_{120}/Bio_{32}/$FITC_{10}$ by Aggregation with Concanavalin A (Con A)

A solution of Con A (17 µM in 20 mM Tris-HCl, 250 mM NaCl, 1 mM $CaCl_2$, 1 mM $MnCl_2$, pH 6.2) was added in 10 µL portions to a solution of 3[G4]-Man_{120}/Bio_{32}/$FITC_{10}$ (300 µL, 1.67 mM of mannose in 20 mM Tris-HCl, 250 mM NaCl, 1 mM $CaCl_2$, 1 mM $MnCl_2$, pH 6.2). After each addition, the mixture was allowed to stand for 5 min before the absorbance at 700 nm was recorded and a new Con A portion was added. The process was repeated till absorbance reached a constant value. At this point, addition of a saturated solution of α-methyl-D-mannopyranoside (10 µL) recovered the original absorbance value. When the same experiment was done with a 17 µM solution of lysozyme (a protein not recognizing mannose taken as control) in the same buffer, no variation in absorbance was observed.

8. Cell Cultures and Cell Studies

Human adenocarcinoma alveolar basal epithelial (A549) cells, obtained from the European Collection of Authenticated Cell Cultures (ECACC), were cultured at 37 °C in a 5% CO_2 atmosphere in Dulbecco's modified Eagle's medium (DMEM) with high glucose, containing 10% fetal bovine serum (FBS) and supplemented with 50 U/mL penicillin and 50 U/mL streptomycin. All cell experiments were performed with this modified DMEM, referred in the text as "medium".

Cellular Uptake of DOX-loaded PEG-[G3]-Cat Micelles. A549 cells were seeded on a 4-chamber glass bottom 35 mm dish with 20 mm bottom well from In Vitro Scientific in 0.5 mL of medium (density of 150000 cells/mL) and incubated overnight at 37 °C in 5% CO_2 atmosphere. Then, medium was replaced by a solution of 30 μL of DOX-loaded PEG-[G3]-Cat micelles in 470 μL of medium, and incubation was continued at 37 ºC for 30 min. Afterwards, medium was removed, and fresh medium was added to the cell culture plates. Cells were left incubating at 37 ºC for 1 h. Half an hour before analysis by confocal microscopy, acidic organelles were stained by adding 10 μL of 10 μM LysoTracker Green and nuclei were stained by adding 10 μL of 200 μM Hoechst 33258. Before observation, medium was replaced with fresh medium.

The intracellular distribution of DOX was studied in a Laser Scanning Confocal Inverted Microscopy Leica Stellaris 8 FALCON (Leica Microsystems, Wetzlar, Germany) equipped with Leica Application Suite X (LAS X) package. Excitation/emission wavelengths were 405 nm/415-482 nm for Hoechst 33258; 490 nm/500-540 nm for LysoTracker Green; and 440 nm/580-650 nm for DOX. Hybrid detectors were used for detecting fluorescence signal and a PMT for capturing brightfield images. Images were acquired applying a sequential method with a 1296 x 1296 pixels resolution (82 μm × 82

μm). The glycerol immersion employed objective was HC PL APO CS2 63x/1.30 Gly. Finally, resolution was optimized by applying Leica Lightning adaptative deconvolution.

Cellular Uptake of 3[G4]-Man_{120}/Bio_{32}/$FITC_{10}$. A solution of Bio-Cy5 [2.1 μL, 0.1 mg/mL in phosphate buffered saline (PBS), 0.20 nmol] was added to a solution of SA (11 μL, 1 mg/ml in PBS, 0.20 nmol). Then, a solution of 3[G4]-Man_{120}/Bio_{32}/$FITC_{10}$ (45 μL, 0.1 mg/mL in PBS, 0.05 nmol) was added and the resulting solution was left at 21 ºC for 1 h under orbital stirring (300 rpm) in an Eppendorf Thermomixer C to afford the dendrimer–SA–Bio-Cy5 complex. Control samples for cell internalization studies were prepared following the same experimental procedure replacing the dendrimer or SA with PBS.

A549 cells were seeded on a 4-chamber glass bottom 35 mm dish with 20 mm bottom well from In Vitro Scientific in 0.5 mL of medium (density of 150000 cells/mL) and incubated overnight at 37 °C in a 5% CO_2 atmosphere. Then, the medium was replaced by a solution of 30 μL of the dendrimer–SA–Bio-Cy5 complex in 470 μL of medium, and incubation was continued at 37 ºC for 2 h. Before analysis by confocal microscopy, acidic organelles were stained by adding 10 μL of 10 μM LysoTracker Red, and nuclei were stained with Hoechst 33342 following the manufacturer's protocol. Before observation, medium was replaced with fresh medium.

Then, confocal images were obtained on an Andor Dragonfly spinning disk confocal system mounted on a Nikon TiE microscope equipped with a Zyla 4.2 PLUS sCMOS digital camera (Andor, Oxford Instruments) and an OKO-lab incubator to maintain cells at 37 °C. Samples were excited with four different lasers (405, 488, 561, and 637 nm) and the emitted fluorescence was collected by the filter wheel (450/50 nm, 525/50 nm, 620/50 nm, and 725/40 nm) with appropriate combinations of them.

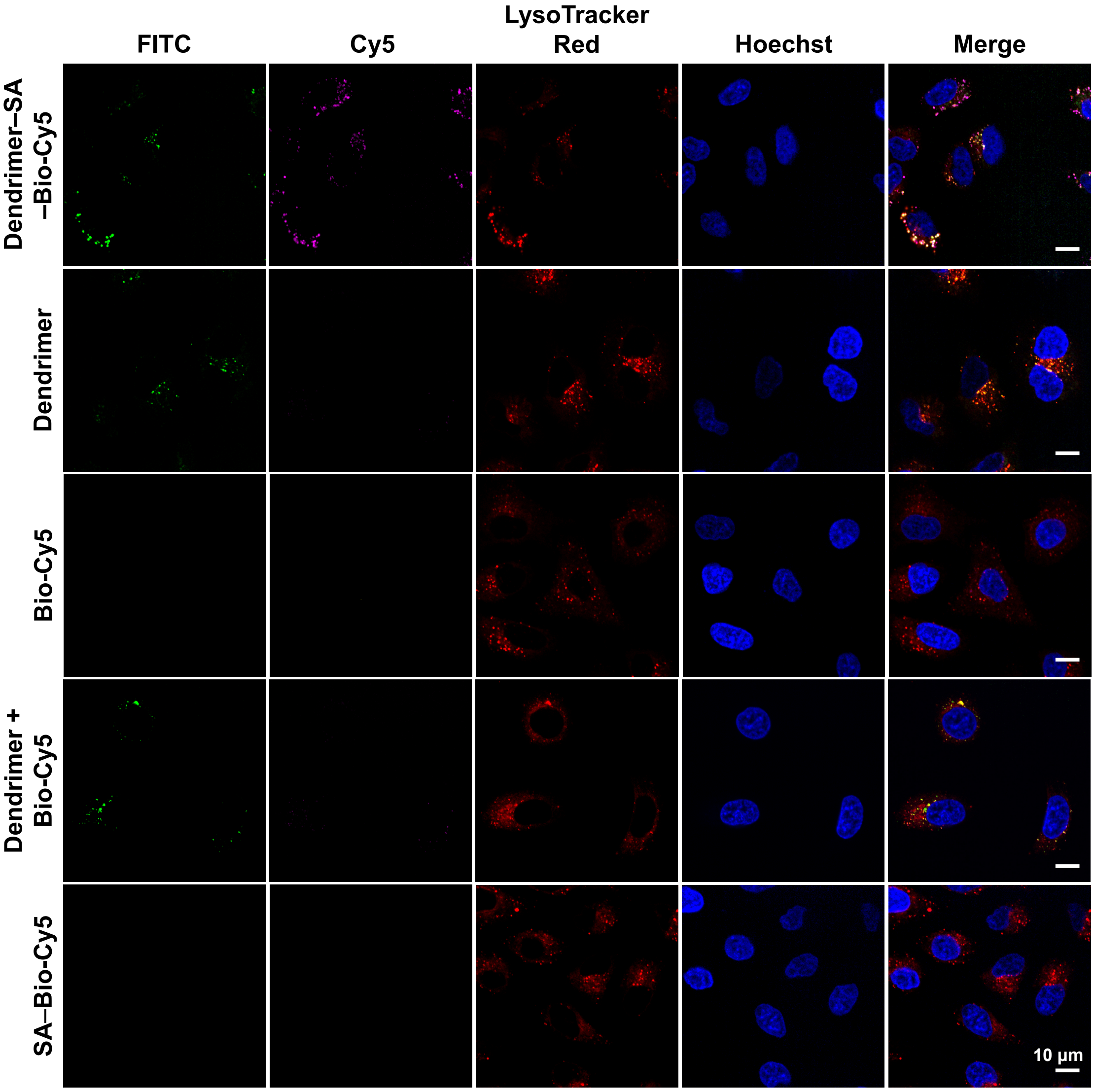

Figure S3. Assessment of 3[G4]-Man$_{120}$/Bio$_{32}$/FITC$_{10}$ as a nanocarrier for cell internalization. LSCM images of A549 cells incubated for 2 h with the dendrimer–SA–Bio-Cy5 complex, dendrimer, Bio-Cy5, a mixture of the dendrimer and Bio-Cy5, and the SA–Bio-Cy5 complex. Dendrimer stained in green (FITC), Bio-Cy5 stained in magenta (Cy5), endosome/lysosome stained in red (LysoTracker Red), nuclei stained in blue (Hoechst).

9. References

1. Amaral, S. P.; Fernandez-Villamarin, M.; Correa, J.; Riguera, R.; Fernandez-Megia, E. Efficient Multigram Synthesis of the Repeating Unit of Gallic Acid-Triethylene Glycol Dendrimers. *Org. Lett.* **2011**, *13*, 4522-4525.

2. Bräse, S.; Gil, C.; Knepper, K.; Zimmermann, V. Organic Azides: An Exploding Diversity of a Unique Class of Compounds. *Angew. Chem., Int. Ed.* **2005**, *44*, 5188-5240. and references therein.

3. Kolb, H. C.; Finn, M. G.; Sharpless, K. B. Click Chemistry: Diverse Chemical Function from a Few Good Reactions. *Angew. Chem., Int. Ed.* **2001**, *40*, 2004-2021. and references therein.
